# Novel reference genes in colorectal cancer identify a distinct subset of high stage tumors and their associated histologically normal colonic tissues

**DOI:** 10.1186/s12881-019-0867-y

**Published:** 2019-08-13

**Authors:** Lai Xu, Helen Luo, Rong Wang, Wells W. Wu, Je-Nie Phue, Rong-Fong Shen, Hartmut Juhl, Leihong Wu, Wei-lun Alterovitz, Vahan Simonyan, Lorraine Pelosof, Amy S. Rosenberg

**Affiliations:** 1 OBP/DBRR-III, CDER, FDA, Silver Spring, MD 20993 USA; 2 Facility for Biotechnology Resources CBER, FDA, Silver Spring, MD 20993 USA; 3 Indivumed GMBH, 20251 Hamburg, Germany; 4 OCS/NCTR/DBB, FDA, 3900 NCTR Road, Jefferson, AR 72079 USA; 5 OBE/HIVE CBER, FDA, Silver Spring, MD 20993 USA; 6 Office of Hematology and Oncology Products CDER, FDA, Silver Spring, MD 20993 USA; 7 Silver Spring, USA

**Keywords:** Colorectal reference genes, High stage tumors, And molecular abnormalities in tumor adjacent tissues

## Abstract

**Background:**

Reference genes are often interchangeably called housekeeping genes due to 1) the essential cellular functions their proteins provide and 2) their constitutive expression across a range of normal and pathophysiological conditions. However, given the proliferative drive of malignant cells, many reference genes such as beta-actin (*ACTB*) and glyceraldehyde-3-phosphate-dehydrogenase (*GAPDH*) which play critical roles in cell membrane organization and glycolysis, may be dysregulated in tumors versus their corresponding normal controls

**Methods:**

Because Next Generation Sequencing (NGS) technology has several advantages over hybridization-based technologies, such as independent detection and quantitation of transcription levels, greater sensitivity, and increased dynamic range, we evaluated colorectal cancers (CRC) and their histologically normal tissue counterparts by NGS to evaluate the expression of 21 “classical” reference genes used as normalization standards for PCR based methods. Seventy-nine paired tissue samples of CRC and their patient matched healthy colonic tissues were subjected to NGS analysis of their mRNAs.

**Results:**

We affirmed that 17 out of 21 classical reference genes had upregulated expression in tumors compared to normal colonic epithelial tissue and dramatically so in some cases. Indeed, tumors were distinguished from normal controls in both unsupervised hierarchical clustering analyses (HCA) and principal component analyses (PCA). We then identified 42 novel potential reference genes with minimal coefficients of variation (CV) across 79 CRC tumor pairs. Though largely consistently expressed across tumors and normal control tissues, a subset of high stage tumors (HSTs) as well as some normal tissue samples (HSNs) located adjacent to these HSTs demonstrated dysregulated expression, thus identifying a subset of tumors with a potentially distinct and aggressive biological profile.

**Conclusion:**

While classical CRC reference genes were found to be differentially expressed between tumors and normal controls, novel reference genes, identified via NGS, were more consistently expressed across malignant and normal colonic tissues. Nonetheless, a subset of HST had profound dysregulation of such genes as did many of the histologically normal tissues adjacent to such HSTs, indicating that the HSTs so distinguished may have unique biological properties and that their histologically normal tissues likely harbor a small population of microscopically undetected but metabolically active tumors.

**Electronic supplementary material:**

The online version of this article (10.1186/s12881-019-0867-y) contains supplementary material, which is available to authorized users.

## Background

Basic cellular functions are supported by guaranteed expression of genes encoding proteins mediating important proteins for cellular integrity. Such genes have been referred to as “housekeeping” genes or, for purposes of comparison of gene expression levels across different cell populations, as “reference” genes. While all cells require the functions of proteins encoded by such genes, the uniformity of expression levels in distinct cells and tissues is not confirmed, as diverse physiological conditions and disease states impose different metabolic and structural requirements [1–4]. Often utilized or “classical” reference genes have been identified with roles in essential biological processes including molecular transport, RNA metabolism, oxidative phosphorylation, proteolysis, protein translation, regulation of protein metabolism and cell cycle control [5]. Although various tools like Genorm, NormFinder, or BestKeeper have each defined a suitable set of classical reference genes for specific qPCR studies, recent cancer studies found that normalization of gene expression levels using classical *ACTB* and *GAPDH* introduced artifacts in qPCR results because of non-uniformity of reference gene expression in mouse fibroblasts [6] and in human cancer lines [7]. Since NGS is quantitated directly as fragments per kilobase per of transcript per million mapped reads (FPKM), without the need for normalization by reference genes, we used NGS to examine both relative and absolute gene expression levels of 21 classical reference genes in CRC and their respective normal tissues. We also inquired into the presence of no novel reference genes, better suited for quantitative purposes in PCR based assays, based on limited CV across 79 CRC tumor pairs.

## Methods

### Original CRC cohort

Seventy-nine paired-tissues (79 tumor and 79 normal controls, Additional file 1: Table S1) of pretreatment CRCs were collected from 38 male and 41 female patients by Indivumed GmbH (Germany) for mRNA sequencing. The purchase of these samples was approved by U. S Food & Drug Administration Institutional Review Boards and Research Involving Human Subjects Committee. To evaluate tumor content, hematoxylin and eosin stained microscopic slices were examined by pathologists to determine the tumor cell and normal cell areas, respectively. Histologically, tumor samples had 50–70% content of cancer cells while normal samples had 0% content of cancer cells. Normal tissues were collected from a site at a minimum of 5 cm from the tumor margin. Ischemia time was 6–11 min. This short cold ischemia reduces postsurgical tissue processing artifacts [8]. According to the medical pathology report, tumors were classified as well, moderately, and poorly differentiated tumors following international guideline UICC TNM-classification [9]. For the convenience of analysis, 26 stage I and II tumors were considered as low stage tumors (LSTs), while 53 stage III and IV tumors were considered as HSTs. In this study, a normal control adjacent to a low stage tumor is referred as LSN. The ratio of high stage tumors vs. low stage tumors is 2 to 1. Among 26 low stage tumors, there were 2 either lymph node (LN) or lymphatic vessel (LV) positive tumors while among 53 high stage tumors, there were 28 either LN/LV positive tumors. For tumor grades, there were 17 well (low grade) differentiated, 36 moderately (medium grade) differentiated, and 26 poorly (high grade) differentiated tumors. Clinical and histopathological characteristics of the patients as well as tumor location are summarized in Additional file 1: Table S1. Among these 80 tumor pairs, 79 pairs were sequenced except the T7/N7 pair [10–12].

### TCGA CRC validation cohort

Because, we studied reference genes across both tumor and normal samples, we only selected the patient matched 50 CRC pairs (100 samples) available from TCGA38 from OncoLand (TCGA38 contains 50 paired CRCs and 589 unpaired CRCs). In respect of reference genes as potential biomarkers for HST/HSN, we specifically compiled tumor stage information for 50 CRCs. Due to the fact that single data banks, such as CBioPortal, do not contain all of the relevant CRC information, we had to extract tumor staging information for 50 CRCs from three different data banks (the Human Protein Atlas, the Stanford Cancer Genome Atlas Analysis of colorectal cancer and cBbioPortal) (https://www.proteinatlas.org/news/tag/tcga, http://genomeportal.stanford.edu/tcga-crc/get_feature_samples?filename=Y_COADREAD_2013-01-16_CancerGenes_Integrative_ClinicalStage.txt and https://www.cbioportal.org). As result, this 50 CRC cohort contains 32 low stage (I/II) CRC pairs (64 LST/LSNs) and 18 high stage (III/IV) CRC pairs (36 HST/HSNs) (Additional file 1: Table S2). To validate results obtained from our 79 paired samples, gene expression (FPKM) information related to 6 CRC hallmark genes, 21 classical reference genes, 42 novel reference genes and 8 reference gene coexperssed genes of 50 CRC pairs were downloaded.

### mRNA sequencing

RNA quality was assessed using the Agilent 2100 Bioanalyzer, with cellular RNA analyzed using the RNA 6000 Nano Kit (Agilent). Samples with an RNA Integrity Number (RIN) of 7 or higher were processed to generate libraries for mRNA sequencing following the Illumina® TruSeq Stranded mRNA Sample Preparation Guide. In this method, poly-A mRNAs were purified from 0.5 μg total RNA, fragmented and reverse-transcribed into cDNAs. Double strand cDNAs were adenylated at the 3′ ends and ligated to indexed sequencing adaptors, followed with briefly amplification for 15 cycles. One femtomole of the sequencing libraries (median size ~ 260 nt) were denatured and loaded onto a flow cell for cluster generation using the Illumina cBot. Every six samples were loaded onto each lane of a rapid run flow cell. Paired-end sequencing was carried out on HiSeq 2500 sequencer (Illumina, San Diego, CA, USA) for 100 × 2 cycles. For each sample, we obtained ~ 50 million 100-bp reads that passed preset filtering parameters [10–12].

### Sequencing data analysis

For mRNA sequencing, Tophat V.2.0.11 was used to align reads in fastq files to the UCSC human hg19 reference genome. Cufflinks V.2.2.1 was used to assemble the transcriptome based on the hg19 reference annotation, and Cuffquan/Cuffnorm (part of Cufflinks) were used in calculating relative abundance of each transcript reported as FPKM. ANOVA test was conducted (on Partek genomics suite) to identify mRNAs with differential expression between tumors and matched normal adjacent tissues using the threshold False Discovery Rate (FDR) ≤ 0.05. The unsupervised hierarchical clustering analysis (HCA) and principal component analysis (PCA) were used to explore the gene expression profiles on ArrayTrack (the National Center for Toxicological Research, U.S. Food and Drug Administration). The FPKMs from samples were log_2_ transformed and then z-score transformed for HCA and PCA plot. We determined tumor and normal sample outliers in PCA results as in our previous study [11, 12]. In brief, we manually picked a center point and used L2 distance to determine whether one node is inside or outside a boundary marked by a dashed circle. Then, CHITEST (excel 2016) was used to determine the differential location between HST/HSNs and LST/LSNs in PCA. The Student’s t-test (excel 2016) was used to detect differential CV between low and high stage tumors while Pearson correlation analysis was to detect the correlation between NGS and qPCR. The reference gene co-expression analyses were carried by Partek NGS & microarray data analysis software. Correlations were transformed to Fisher’s z-score using online tool (http://onlinestatbook.com/calculators/fisher_z.html) before averaging and retransforming with an inverse Fisher-Z. Gene ontology (GO) Integrated Discovery (DAVID) v6.7 (https://david.ncifcrf.gov/*)*, NIAID/NIH. False Discovery Rate (FDR) ≤ 0.05 was used as the criteria for GO category enrichment.

### NGS gene expression landscape of CRC

A total of 25,761 genes were detected. Since genes with higher FPKM values may generally confer more biological impacts, we focused on genes with FPKM > 1 [12]. There were 10,255 genes (40% of total genes) with average FPKM > 1 and differential expression between tumors and normal controls (False Discovery Rate (FDR) < 0.05 in ANOVA). A total of 3893 genes (15% of total genes) with average FPKM > 1 show no differential expression between tumor and normal controls with FDR (ANOVA) > 0.05 [10–12].

### TaqMan quantitative PCR (qPCR) quantification

cDNAs from T16 to T35 pairs (20 tumor pairs) were synthesized from total RNA (0.5 μg) using random primers and High Capacity cDNA Reverse Transcription Kit (ABI Part#4368813). qPCR was performed using an Applied Biosystems 7300 Sequence Detection system. The 10 μl PCR reaction included 0.67 μl cDNA, 1 μl 1× TaqMan Universal PCR master mix, 1 μl primers, and probe mix of the TaqMan Assay protocol (PE Applied Biosystems). The reactions were incubated in a 96-well optical plate at 95 °C for 10 min, followed by 40 cycles of 95 °C for 15 s and 60° for 10 min. The threshold cycle (Ct) is defined as the fractional cycle number at which the fluorescence passes the fixed threshold. The Ct data were determined using default threshold settings. The average Ct values were 32 for *DKC1*, 25 for *RRP1B*, 32 for *BOP1*, 29 for *C1orf43*, 27 for *RAB7A*, 32 for *HEBP4* and 26 for *ACTB*. Individual data points represent mean ± SD of “three biological replicates” in at least separate two experiments. The expression levels of *BOP1* (block of proliferation 1)*, DKC1* (dyskerin pseudouridine synthase 1), and *RRP1B* (ribosomal RNA processing 1B) were examined with genes *ACTB* (beta-actin)*, RAB7A* (ras-related protein Rab-7a), *HEBP2* (heme binding protein 2), and *C1orf43* (chromosome 1 open reading frame 43) as reference genes. ABI PCR primers for qPCR are listed below: *BOP1*: Hs00374884_m1; *DKC1*: Hs00154737_m1; *RRP1B*: Hs00380154_m1; *ACTB*: Hs01060665_g1; *RAB7A*: Hs01115139_m1; *HEBP2*: Hs00204872_m1; and *C1orf43*: Hs00367486_m1. All of these 7 primers were used in qPCR assays from other studies ( [13–18]). The relative expression levels of target genes in tumor samples over normal controls were estimated using 2^-ΔΔCt^ calculation ( [19, 20]).

## Results

### Confirmation of NGS data accuracy with tumor landmark genes

**Fig. 1:**
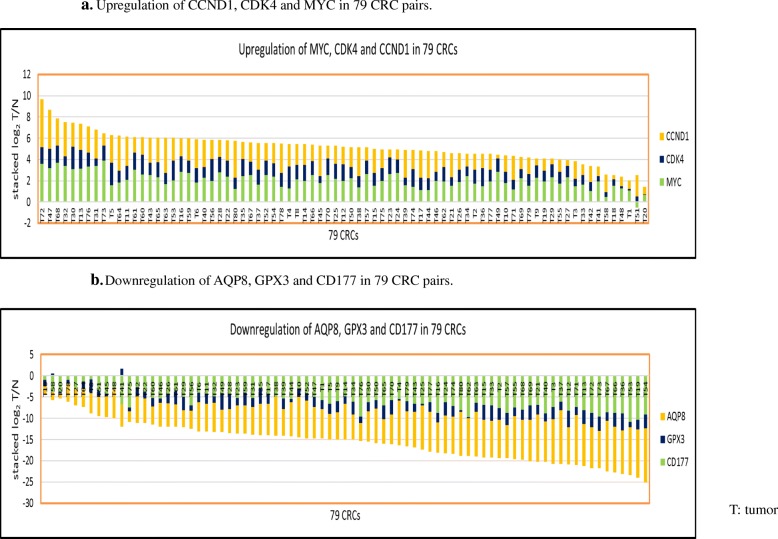
NGS analysis of 6 cancer hallmark gene expression in 79 CRC pairs. **a**. Upregulation of CCND1, CDK4 and MYC in 79 CRCs. **b**. Downregulation of AQP8, GPX3 and CD177 in 79 CRCs

Before investigating the expression of classical reference genes by NGS, we initially examined our 79 CRC cohort for expression levels of known CRC landmark genes including *MYC*, cyclin dependent kinase 4 (*CDK4*) and Cyclin D1 (*CCND1*), as these genes have been shown to be uniformly overexpressed in CRC [21–23]. *MYC* was upregulated in 78 out of 79 CRCs while *CDK4* and *CCND1* were upregulated in all 79 CRCs (log_2_T/*N* > 0) (Fig. 1a). *MYC*, a master regulator of transcription, activates Ras/ERK proliferative pathways [21, 22] while *CDK4* and *CCDN1* contribute to tumor advancement by promoting of G1 phase of cell cycle in CRC [23]. Furthermore, we found that 13 out of 15 genes involved in ribosome biogenesis, a noted hallmark of cancer biology [24], were upregulated in all 79 CRCs (log_2_T/*N* > 0) (Additional file 1: Figure S1a), with tumors clearly separated from normal tissues in HCA as well as PCA (Additional file 1: Figure S1b, 1c). We then examined 3 genes (polycythemia rubra vera protein 1 (*CD177),* aquaporin 8 (*AQP8)* [25] and glutathione peroxidase 3 (*GPX3*)) whose expression is characteristic of normal enterocytes and which are downregulated in CRC as shown in meta-analyses of microarray and immunochemistry studies [5, 6]. *CD177* and *AQP8* were downregulated in all 79 CRCs while *GPX3,* a “tumor suppressor” [26, 27], was downregulated in 75 out of 79 CRCs (log_2_T/*N* < 0) (Fig. 1b). Collectively, these data indicate that our CRC cohort has the expected genetic characteristics of CRC as previously defined and as now characterized by NGS.

### Confirmation of dysregulation among classical reference genes in CRCs

**Fig. 2:**
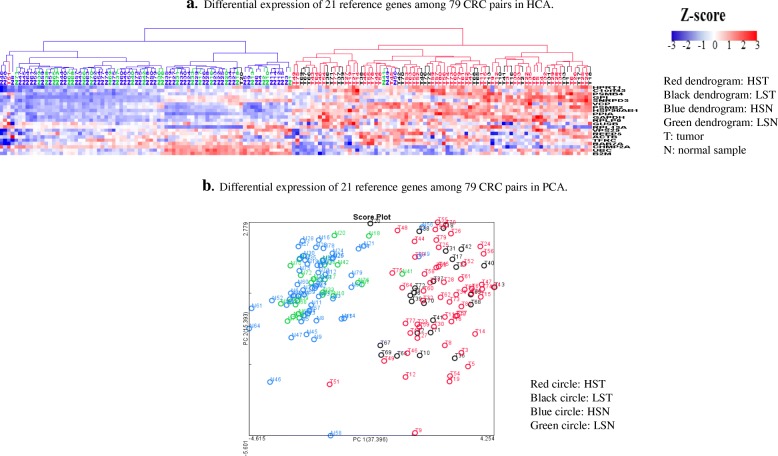
HCA and PCA analysis of 21 classical reference genes in 79 CRC pairs. **a**. Differential expression of 21 reference genes among 79 CRC pairs in HCA. **b**. Differential expression of 21 reference genes among 79 CRC pairs in PCA

As NGS captured the key genetic features of CRC, we then examined whether 21 classical reference genes (Additional file 1: Table S3, S4) used extensively for normalization in qPCR assays, are expressed to a similar degree in CRC and in normal intestinal epithelium. The HCA revealed the clear separation of 77 tumors from 77 normal tissues (Fig. 2a). Furthermore, the stacked log_2_ T/N ratio gene fingerprints showed that these 21 reference genes were upregulated in nearly all CRCs (Additional file 1: Figure S2a). Based on their average log_2_T/N ratio across 79 CRCs, there were 17 upregulated reference genes (ratio range: 0.013 to 1.29), and 4 downregulated reference genes (ratio range: − 0.064 to − 0.65). The PCA profile revealed clear separation of normal samples from tumors (Fig. 2b). Among common reference genes used in PCR based gene expression CRC studies [28, 29], *GAPDH* was more frequently upregulated (log_2_ T/N > 0) in both HST and LST (Additional file 1: Figure S2b) while expression of *ACTB* was either up or downregulated (log_2_ T/N > 0 or < 0) in some LST and HST (Additional file 1: Figure S2c). Importantly, *B2M* (*Beta-2-Microglobulin), essential for expression of MHC class I and thus immunologic targeting of tumor cells by CD8*^*+*^ *T cells, was downregulated* (log_2_ T/N < 0) *in both LST and HST (*Additional file 1: Figure S2d) [30]*.* Of the 21 classical reference genes, only 3 genes (ras-related protein 7a (*RAB7A*), vesicle protein sorting 29 (*VPS29*) and glucuronidase beta *(GUSB*)) showed similar expression levels between tumor and normal tissues (CV < 30% and FDR > 0.05) (Additional file 1: Table S4) and thus may potentially be considered as true reference gene candidates. These results suggest that most classical reference genes are not qualified to serve as expression reference genes in quantitative assays.

### Identification of novel colorectal reference genes

The differential expression of 18 classical reference genes in CRC vs. healthy intestinal tissue led us to explore whether there were reference genes, in addition to the three already identified, that may be more consistently expressed among diverse colonic tissues and could potentially be used for normalization purposes. The candidates are genes with high expression and low variance among tumor and normal samples. By the criteria of FDR > 0.05, CV < 30%, and average FPKM > 100, we found 42 potential colorectal reference genes (Additional file 1: Table S5) that are more consistently expressed among tumor and normal samples (ie, CV across combined 79 tumors and 79 normal tissues were between 15 and 29%). In addition, these 42 newly identified reference genes have smaller variance (average STDEV = 35, CV = 23%) than the 21 classical reference genes (average STDEV = 494, CV = 36%) (Additional file 1: Table S4) in the 79 CRC cohort. The functions of these 42 reference genes include cellular cargo transportation (20 genes in Additional file 1: Table S6), cellular structure (13 genes in Additional file 1: Table S7) and activity in various metabolic pathways (9 genes in Additional file 1: Table S8).

### Subtyping of CRC by novel colorectal reference genes and their coexpressed genes

**Fig. 3:**
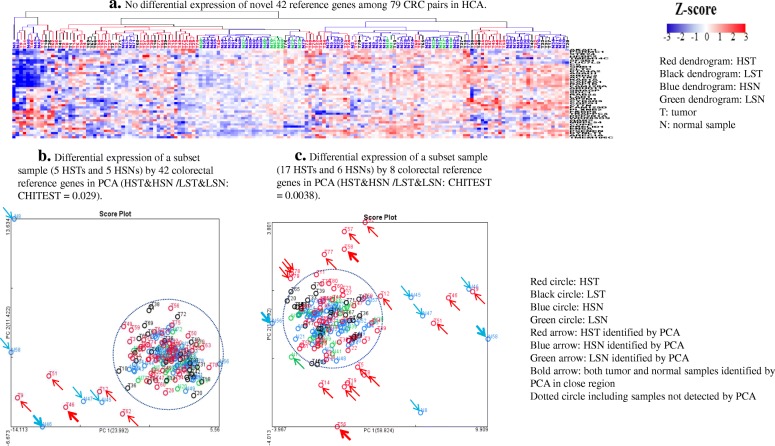
HCA and PCA analysis of colorectal 42 reference genes in 79 CRC pairs. **a**. No differential expression of novel 42 reference genes among 79 CRC pairs in HCA. **b**. Differential expression of a subset sample (5 HSTs and 5 HSNs) by 42 colorectal reference genes in PCA (HST&HSN /LST&LSN: CHITEST = 0.029). **c**. Separation of a subset of 17 HSTs, 6 HSNs and 1 LSN by 8 colorectal reference genes (HST&N /LST&N: Chi square test = 0.0038)

Unlike either the 15 ribosome biogenesis genes (Additional file 1: Figure S1a) or the 18 classical reference genes previously discussed (Additional file 1: Figure S2a), the 42 gene HCA did not separate tumors from normal controls (Fig. 3a). Since this 79 CRC cohort contained 106 HST/HSNs and 52 LST/LSNs, the 42 gene PCA very specifically and clearly separated 5 HSTs as well as 5 HSNs, 9% (10 out of 106) HST/HSN, from the rest of the samples in the PCA (CHITEST: *P* = 0.029) (Fig. 3b). To identify the critical genes which could effectively delineate this distinct set of HST/HSN, among the 42 CRC reference genes, 8 reference genes (*CLTC, SDC1, FAM120A, ARPC5, HEBP2, RAB1A, RAB1B, ACTR2*) with relatively greater CV values among HST were selected (Additional file 1: Figure S3a). This is based on previous findings that the distinct subsets of HST/HSN, which locate at peripheral regions of PCAs, have larger gene expression CV values [11, 12]. The PCA of these 8 reference genes distinguished 17 HSTs and 6 HSNs, 21% (23 out of 106) HST/HSN, as well as 1 LSN, (2% (1 out of 52) LST/LSN, from the rest of samples in PCA (CHITEST: *P* = 0.0038) (Fig. 3c). Ten of these 17 HSTs had metastases detected in either the local lymph node or lymphatic vessel (LN/LV), and 4 out of 6 of the adjacent “normal” tissues were associated with such LN/LV metastatic positive tumors (Additional file 1 Table S1). Interestingly, this 8 gene PCA did not distinguish 2 LSTs with LN/LV metastasis from 24 LSTs without LN/LV metastasis (Additional file 1: Table S1). Compared to 26 LSNs, these 8 reference genes were mainly downregulated by more than 4-fold in HSTs and HSNs (Additional file 1: Figure S3b, S3c).

**Fig. 4:**
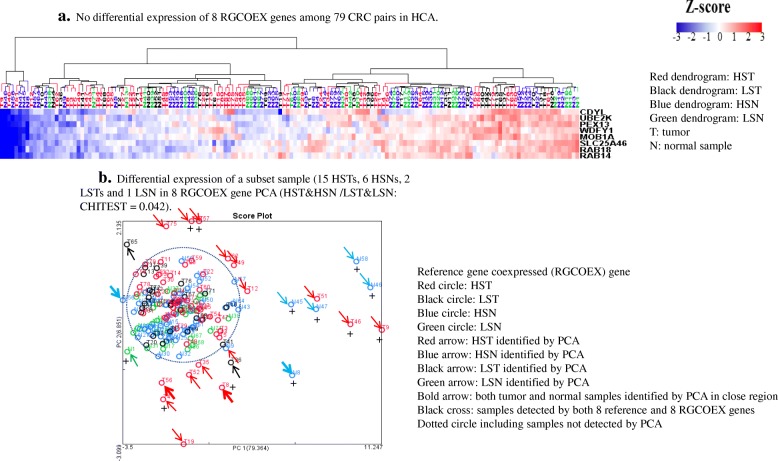
HCA and PCA analysis of 8 RGCOEX genes in 79 CRC pairs. **a**. No differential expression of 8 RGCOEX genes among 79 CRC pairs in HCA. **b**. Differential expression of a subset sample (15 HSTs, 6 HSNs, 2 LSTs and 1 LSN in 8 RGCOEX gene PCA (HST&HSN /LST&LSN: CHITEST = 0.042)

Because co-expressed genes are often involved in the same biological pathways and processes [20, 21], we investigated the functions of these 8 reference genes, the biological pathways in which they participate, and their coexpressed genes. Pearson’s correlation analysis identified a total of 1930 coexpressed genes with 6 of 8 of these reference genes (absolute correlation coefficient cc > 0.6) while 2 reference genes lacked any coexpressed genes. David Bioinformatic analysis showed that these reference gene coexpressed (RGCOEX) genes related to intracellular transport (Additional file 1: Table S9, S10, S11), consistent with published results (Additional file 1: Table S6). Furthermore, since these 8 CRC reference genes detected HST/HSN in PCA, the coexpressed genes may also detect these HST/HSNs as well. Thus, we selected the top 8 RGCOEX genes (*CDYL, MOB1A, PEX13, WDFY1, SLC25A46, UBE2K, RAB14, RAB18*) (cc > 0.70) for HCA and PCA. As with the 8 reference genes, the 8 RGCOEX genes did not separate most tumors form normal tissues in HCA (Fig. 4a) but identified a set of HST/HSN (15 HSTs and 6 HSNs), 20% (21 out of 106) HST/HSN, as well as 2 LSTs and 1 LSN, 6% (3 out of 52) LST/LSN from rest of samples in PCA (CHITEST: *P* = 0.042) (Fig. 4b). Importantly, there was concordant detection of 13 tumor samples between PCAs of the 8 reference gene panel (Fig. 3c) and 8 RGCOEX genes (Fig. 4b). Thus, the distinguishing PCA signature of the 8 reference gene panel for HSTs and HSNs is validated significantly by the PCA of their RGCOEX genes. As was the case for the 8 reference gene panelists, these 8 RGCOEX genes also had relatively narrow CVs (CV range; 25 to 37%) (Additional file 1: Table S12) and were principally downregulated by 4 fold in the HSTs and HSNs compared to 26 LSNs (Additional file 1: Figure S4a, S4b).

### Molecular indicators of tumor in ‘histological normal’ tissues detected by the novel reference gene panel

Since the PCA signatures of the 8 novel reference genes (Fig. 3b, c) clustered 1 LSN (N1) and 6 HSNs (N8, N45, N46, N47, N56, N58) along with a subset of HST, it was important to verify whether these so-called normal samples may contain undetected tumor, as revealed by similar gene expression profiles as their respective tumors. We thus evaluated and compared the expression levels of the 8 reference genes as well as the RGCOEX genes in these histologically normal samples and their respective tumor samples. Interestingly, 5 HSNs (N45, N46, N47, N58, N8) manifest a similar downregulation of gene expression in genes marking normal enterocytes, but to an even greater extent, than their respective tumors (T45, T46, T47, T58, T8) while N1, and N56 manifest distinctly different patterns from their associated tumors (T1, T56) (Additional file 1: Figure S5a-f). The reason for the greater extent of gene downregulation in the 5 normal samples compared to their paired tumors is intriguing and needs further evaluation. To further our evaluation of the potential presence of tumor within these 7 HSNs, we additionally examined the expression of genes characteristic of tumor microenvironments including the following: desmoplasia (dense fibrosis around a neoplasm) genes, including those pertaining to the collagen (*COL6A1, COL6A2, COL1A2, COL1A1)*; neutrophil/*myeloid-derived suppressor cell* infiltration (*CXCR1, CXCR2*); cell proliferation (*MYC, CDK4*) and tumor invasion (*MMP2, MMP9, MM14*), (Additional file 1: Figure S6a-d). Strikingly among 7 HSNs, N8 (adjacent to a stage 4 poorly differentiated tumor with LN metastasis/R0) had the highest expression of *MMP2, MMP14, COL6A1, COL6A2, COL1A2, COL1A1, CXCR1* and *CXCR2* across all 79 normal samples while N58 (adjacent to a stage 3 well differentiated tumor with LN metastasis/R2) had the highest expression *MYC* and *CDK4* across all 79 normal samples. These data indicate that histologically normal tissues, may contain undetected tumor, profoundly altering patient prognosis and strongly indicating that rapid evaluation of tumor margins by more advanced technologies may improve surgical resection of malignant tissue and confer improved patient survival.

### Computing normalization with 6 typical reference genes in NGS data

As most classical reference genes were found to be differentially expressed in CRC versus adjacent normal tissues (Additional file 1: Table S3, Fig. 2a, b), we investigated whether the CV values of reference genes impacted their ability to serve as reference genes for normalization of gene expression. Considering that the genes related to ribosome biogenesis are uniformly and significantly elevated in the CRC cohort (Additional file 1: Figure S1a, 1b), we simulated normalization of ribosome biogenesis related genes, with CRC reference genes of differing CVs. In doing so, we normalized the FPKM values of 15 ribosome biogenesis related genes using the FPKM values of 6 reference genes (*C1orf43, RAB7A, HEBP2* (Heme Binding Protein 2)*, ACTB, TFRC* (Transferrin Receptor) and *HSP90AB1* (Heat Shock 90kD Protein 1, Beta)) whose CVs range from 16 to 75% (Additional file 1: Table S3, S4) and compared the separation of tumors and normal samples in PCAs (Additional file 1: Figure S7). The analysis revealed that normalization with a “hypothetical” reference gene with 0% CV across all 79 tumor and 79 normal samples (FPKM = 100, CV = 0%) maintained the separation of tumors from normal samples as did normalization with *RAB7A* (CV = 17%) and *C1orf43* (CV = 16%). However, normalization using *HSP90AB1* (CV = 75%) completely abrogated the separation of tumors and normal samples including high stage tumors, while normalization using genes of intermediate CV, *TFRC* (CV = 52%), *ACTB* (CV = 30) and *HEBP2* (CV = 21%), separated tumors from normal samples to a variable extent. Collectively, these observations indicate that reference genes with lowest CVs, such as *RAB7A* and *C1orf43,* could serve as better reference genes for gene expression normalization in PCR based assays.

### Experimental normalization with 4 typical reference genes in qPCR

To further test the fitness of the above reference genes for use as normalization values in qPCR based assays, we examined the expression of three ribosome biogenesis related genes (*BOP, DKC1,* and *RRP1B*) by a TaqMan qPCR assay using selected reference genes *ACTB* (CV = 34%), *HEBP2* (CV = 21%), *RAB7A* (CV = 17%), and *C1orf43* (CV = 16%) for gene expression normalization in 20 CRC pairs (T16 to T35). Compared to the NGS data in which all 20 tumors displayed upregulated *BOP, DKC1* and *RRP1B*, the qPCR assays (Additional file 1: Figure S8a1–3) revealed upregulated *BOP1, DKC1,* and *RRP1B* in only 13 of 20 tumor samples, regardless of which individual reference gene or combinations of 4 reference genes were used for normalization. The expression correlation between the NGS experiments and qPCR assays (Additional file 1: Figure S8b1–3) was weak (Pearson’s correlation coefficients (cc) < 0.4), with the average Pearson’s correlation coefficient values for expression normalized by *ACTB, HEBP4, RAB7A,* and *C1orf43* of 0.064, 0.167, 0.357 and 0.327, respectively. The data demonstrate that experimental normalization of actual qPCR data using reference genes with smaller CVs (*C1orf43* and *RAB7A*) is comparable to normalization using those with larger CVs (*ACTB* and *HEBP2*) and discordant with NGS findings. Thus, we found inconsistent normalization results derived from the same reference genes in different assays (NGS and qPCR).

### Validation of both classical and CRC reference gene sets in The Cancer Genome Atlas (TCGA)

**Fig. 5:**
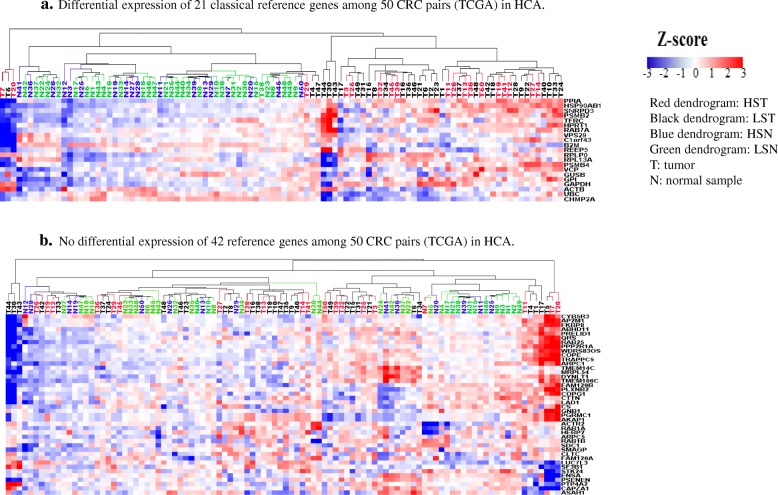
HCA and PCA analysis of reference genes in 50 CRC pairs (TCGA). **a**. Differential expression of 21 classical reference genes among 50 CRC pairs (TCGA) in HCA. **b**. No differential expression of 42 reference genes among 50 CRC pairs (TCGA) in HCA

**Fig. 6:**
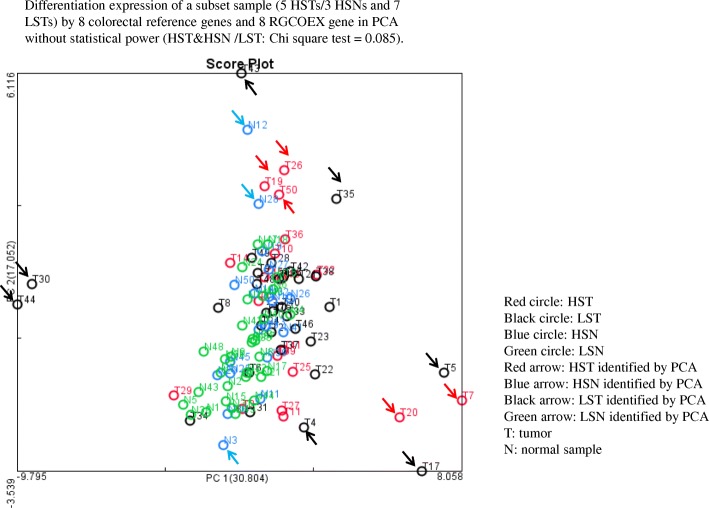
PCA analysis of combination with 8 reference genes and 8 RGCOEX genes in 50 CRC pairs (TCGA). Differentiation expression of a subset sample (5 HSTs/3 HSNs and 7 LSTs) by 8 colorectal reference genes and 8 RGCOEX gene in PCA without statistical power (HST&HSN /LST: Chi square test = 0.085)

Finally, to confirm differential expression of the 21 classical reference genes as well as the general lack of differential expression of our newly identified 42 colorectal reference genes, we examined these two sets of genes in 50 CRC pairs (TCGA_B38) which contains 32 low stage (I/II) CRC pairs (64 LST/LSN) and 18 high stage (III/IV) CRC pairs (36 HST/HSN) (Additional file 1: Table S2). To establish the basis of validation, we first checked the upregulation of *MYC, CCDN1* and *CDK4* as well as the downregulation of *AQP8, CD177* and *GPX3* in this 50 CRC cohort. All 3 oncogenes in 50 CRCs, except for *MYC* in one tumor and *CDK4* in three tumors, were upregulated (Additional file 1: Figure S5a) and 3 normal colonic physiological genes were downregulated, except for *CD177* in one tumor, AQP8 in two tumors and *GPX3* in three tumors. These data suggest that the TCGA CRC cohort gene expression profile is comparable to our 79 CRC cohort (Fig. 1). We then examined the expression of 21 classical and 42 novel reference genes in the TCGA CRC cohort. As expected, the 21 classical reference genes were differentially expressed in HCA (Fig. 5a) while the 42 novel reference genes were not differentially expressed (Fig. 5b). In TCGA, the variation of 21 classical reference genes (average STDEV = 448, average CV = 46%, Additional file 1: Table S13) was larger than the variation observed in the 42 novel reference genes (average STDEV = 57, average CV = 36%, Additional file 1: Table S14). Thus, the TCGA data further support the 42 novel reference gene panel as better reference genes than the 21 classical reference genes. With respect to detection of a subset of CRC with potentially unique biological characteristics, the PCA of the 8 reference genes as well as 8 RGCOEX genes distinguished 5 HSTs/3 HSNs, 22% (8 out of 36) HST/HSN and 7 LSTs, 10% (7 out of 64) LST/LSN from rest of samples, but statistical significance was lacking (CHITEST: *P* = 0.085) (Fig. 6). Since this cohort contained less HST/HSN and more LST/LSN, the detection of HST/HSN needs to be further validated in a CRC paired cohort containing more HST/HSN in further studies.

## Discussion

The emergence of *NGS* enables absolute quantitative analyses of the transcriptome across different biological samples in a highly sensitive and precise manner, with consequent direct analysis and comparison of gene expression [12, 31]. Here we found that by NGS analysis, 21 classical reference genes (including *GAPDH, ACTB, RPLP0, PPIA* and *B2M*) pertaining to biological functions and processes including cell cycle, ribosome biogenesis, glycolysis, angiogenesis, apoptosis and inflammation and commonly used for the normalization of gene expression in qPCR studies, had differential expression in CRC tumors vs. normal tissues. We then identified 42 CRC reference gene candidates, distinct from the 21 reference gene panel, that had lower CVs and minimal differential expression in tumors vs histologically normal tissues. These 42 CRC reference genes have been frequently cited in published CRC studies or recommended by NormFinder for colon tissue studies [32–36]. The differential expression of 21 classical reference genes and minimal differential expression of the novel 42 CRC reference genes were further evaluated and validated in a TCGA cohort. Despite the more homogenous expression of the 42 CRC reference genes between tumor and normal tissue, PCA of 8 of these CRC reference genes identified a distinct subset of HST/HSN which may have distinct biological properties. This 8 reference gene subset was validated for detection of the distinct HST/HSN subset of tumors by PCA of highly coexpressed genes. Because this unique subset of colonic tissue reference genes mainly pertains to intracellular transport, the downregulation of these genes in HST/HSN likely indicates loss of brush border nutrient transport, a major physiological function of normal enterocytes. Furthermore, some additional reference genes may act as tumor suppressors since *CLTC* (vesicle traffic protein) (Additional file 1: Table S6), *ACTR2* (cytosolic transport related protein) (Additional file 1: Table S6) and *RAB1B* (ras-like shuttle protein) (Additional file 1: Table S6) were also positively (cc > 0.6) coexpressed (same trend) with 11 tumor suppressor genes mainly related to rho guanine nucleotide exchange factor, succinate dehydrogenase complex and TP53 and negatively (cc < − 0.6) correlated with expression of 4 oncogenes as well as 11 ribosome biogenesis genes (Additional file 1: Table S15). Thus, the downregulation of such reference genes could be translated as a shift from normal cellular functions and well-behaved growth inhibition to highly proliferative cells equipped for tumor metastases in this subset of HSTs. Building on our previous study which captured increased cell proliferation, glycolysis, inflammation, collagen catabolism, and decreased lipid metabolism, colonic cellular transportation and detoxification as indispensable hallmarks for CRC [12], the novel colorectal reference genes mainly related to intracellular/cytosolic transport, here identified as highly dysregulated in a subpopulation of HST/HSN, may have identified tumors with unique biological characteristics with clinical implications. Further study is clearly needed. Moreover, this study has important implications for defining “clear” tumor margins, as despite having histologically normal tumor margins, two HSNs highly likely contained significant tumor content as assessed by the downregulated reference genes and the upregulation of genes relating to cell proliferation, invasion, fibrosis and neutrophil infiltration highly characteristic of a tumor microenvironment. Interestingly, there was concomitant downregulation of 3 reference genes (*RAB1B, ACTR2* and *CLTC*) and 3 tumor suppressor genes (neurofibromatosis type 1 (*NF1*)*,* DEAD-Box helicase 5 (*DDX5)* and CAMP responsive element binding protein 1 (*CREB1)*) in 3 out of 6 HSNs detected by the 8 reference gene PCA (Additional file 1: Figure S10 and Additional file 1: Table S15). These 3 histologically normal tissues were adjacent to either poorly differentiated HST or HST with lymph node metastasis. Moreover, the FPKM patterns of the reference genes distinctly revealed clonal similarities between five “normal” tissues and their poorly differentiated or local lymph node infiltrated tumors. This phenomenon could be caused by comparable genetic or epigenetic changes pertaining to undetected tumor infiltration or modification of the tumor microenvironment. As the tumor margin impacts overall survival [36, 37], a reference gene evaluation of tumor adjacent tissues, rather than sole reliance on a histological determination, may better determine truly negative margins. Since molecular CRC subtyping could have potential in cancer management [38–42], the clinical outcomes of patients in which tumors expressed profound reference gene dysregulation require further study.

Regarding data normalization, although simulated normalization by reference genes with smaller CVs suggested that such genes may be better reference genes, in actual qPCR assays, the normalized profiles showed very weak correlation with the NGS profiles regardless of the magnitude of CV values of the reference genes. The main reason for this lack of correlation could be intrinsic differences between NGS and qPCR in aspects of sensitivity, specificity and variability [43, 44]. Another likely reason could pertain to differences in the species of mRNA evaluated by the respective assays. NGS gene expression detection is dependent on the poly-A tail “intactness” of mRNA since only pure poly-A mRNA was used as templates for cDNA synthesis, while qPCR gene expression detection is independent of poly-A tail since mRNAs with or without poly-A tail were used for cDNA synthesis by the random sequence primers and sizes of standard amplicons are very short (75–150 bp).

## Conclusions

In summary, we demonstrated the differential expression of 21 classical reference genes in CRC samples vs their histologically normal respective tissues and identified 42 novel reference genes with minimal variability between tumor and normal tissues. From these 42 reference genes, we further determined an 8 gene panel which distinguished a subset of HST/HSN with potentially unique biological properties. In comparing NGS with qPCR, we further demonstrated the clinical potential advantage of using NGS to capture the classical hallmarks of CRC, such as upregulated cell proliferation and downregulated cell differentiation, together with hallmarks of a subset of “high risk” CRC, such as downregulated vesicular transport to potentially improve patient outcome.

## Additional file

Additional file 1: **Table S1.** Clinical information of 79 CRC pairs. The MSI and MSS information were available for 8 tumors. **Table S2.** Tumor information of 50 CRC pairs from TCGA. **Table S3**. 21 classical reference genes and their annotated functions. **Table S4**. Expression profiles of 21 classical reference genes in 79 CRC cohort. **Table S5.** Expression profiles of 42 colorectal reference genes in 79 CRC cohort. **Table S6**. 23 reference genes with the annotated functions of cellular cargo transportation. **Table S7**. 13 reference genes with the annotated functions of structural proteins. **Table S8**. 9 reference genes with the annotated functions of enzymes. **Table S9.** Determination of possible function of 8 reference genes through correlation analysis. **Table S10.** Identification of 8 RGCOEX genes based on their correlation with 5 novel reference genes. **Table S11.** Genes coexpressed with 6 reference genes. **Table S12.** Expression profiles of 8 RGCOEX genes. **Table S13.** Expression profiles of classical 21 reference genes in 50 CRCs (TCGA). **Table S14.** Expression profiles of novel colorectal 42 reference genes in 50 CRC pairs (TCGA). **Table S15.** Oncogenes and tumor suppressors coexpressed with 3 reference genes (RAB1B, ACTR2 and CLTC). **Figure S1.** NGS analysis of 15 genes pertaining to ribosome biogenesis in 79 CRCs. **Figure S1a.** Upregulation of 15 genes pertaining to ribosome biogenesis in 79 CRCs**.** These 15ribosome biogenesis related genes were identified from 1223 upregulated genes (average T/*N* > 2 fold, FDR < 0.05 (ANOVA)) by DAVID Bioinformatics Resources 6.8 (https://david.ncifcrf.gov/*)* [11]. The 15 ribosome biogenesis genes are: D-Tyrosyl-TRNA Deacylase 1 (*DTD1),* Dyskerin Pseudouridine Synthase 1 *(DKC1*), GTP Binding Protein 4 (*GTPBP4),* Ribosomal RNA Processing 1B *(RRP1B)*, Block Of Proliferation 1 (*BOP1),* DDB1 and CUL4 Associated Factor 13 (*DCAF13),* Nucleolar Protein (*NOP2),* Ribosomal RNA processing protein 1 (*RRP1),* Nucleolar And Coiled-Body Phosphoprotein 1 (*NOLC1*), Nucleophosmin 1(*NPM1),* Biogenesis Of Ribosomes 1 (*BRIX1),* Nucleoplasmin 3 (*NPM3),* Ribonucleoprotein 58 (*NOP58),* Ribosomal RNA Processing 9 (*RRP9)* and Ribosome Biogenesis Regulator Homolog 1 (*RRS1*). **Figure S1b.** Differential expression of 15 genes pertaining to ribosome biogenesis among 79 CRC pairs in HCA. **Figure S1c.** Differential expression of 15 genes pertaining to ribosome biogenesis among 79 CRC pairs in PCA. **Figure S2.** NGS analysis of 21 classical reference genes in 79 CRC pairs. **Figure S2a.** Upregulation trend of 21 genes classical reference genes in 79 CRC pairs. **Figure S2b.** Upregulation of GAPDH in a subset of LST and HST. **Figure S2c.** Up and Down regulation of ACTB in a subset of LST and HST. **Figure S2d.** Up and Down regulation of B2M in a subset of LST and HST. **Figure S3.** Expression of 8 reference genes between HST and LST. **Figure S3a**.Selection of 8 reference gene CVs between 53 high stage HSTs and 26 LSTs (t-test: *P* = 0.0003). **Figure S3b.** Fourfold downregulation of 8 reference genes in 15 out 17 of HSTs detected by PCA. **Figure S3c.** Fourfold downregulation of 8 reference genes in 5 out 7 of H/LSNs detected by PCA. For stacked log_2_ ratio plots, FPKMs of 79 tumors and 79 normal samples were only normalized by the mean of FPKM of 26 LSNs for each gene since HSNs were more likely to have dysregulation. **Figure S4.** Downregulation of 8 RGCOEX genes in a subset of tumors and normal controls. **Figure S4a**. Four fold downregulation of 8 RGCOEX genes in 12 out of 16 H/LSTs detected by PCA**. Figure S4b**. Downregulation of 8 RGCOEX genes in 5 out 7 in H/LSNs detected by PCA**.** For stacked log_2_ ratio plots, FPKMs of 79 tumors and 79 normal samples were only normalized by the mean of FPKM of 26 LSNs for each gene since HSNs were more likely to have dysregulation. **Figure S5**. Downregulation of 8 reference genes, their 8 correlated genes and 10 tumor related genes in normal samples and tumors. **Figure S5a**. Downregulation of 8 reference genes in 79 normal samples. **Figure S5b**. Downregulation of 8 reference genes in 79 tumors. **Figure S5c.** Downregulation of 8 RGCOEX genes in a subset of normal samples. **Figure S5d.** Downregulation of 8 RGCOEX genes in a subset of tumors. **Figure S5e.** Downregulation of 8 reference genes in 5 out 7 CRC pairs. **Figure S5 f.** Downregulation of 8 RGCOEX genes in 5 out 7 CRC pairs**. Figure S6.** NGS analysis of tumor related genes in HSN. **Figure S6a**. Upregulation of COL6A1, COL6A2, COL1A2 and COL1A1 in N8. **Figure S6b.** Upregulation of CXCR1 and CXCR2 in N8. **Figure S6c**. Upregulation of MYC and CDK4 in N58. **Figure S6d.** Upregulation of MMP2 and MMP14 in N8. **Figure S7**. PCA Simulated normalization of 15 ribosome biogenesis genes by 7 reference genes in 79 CRCs. **Figure S7a**. Ribosome biogenesis without normalization. **Figure S7b**. Ribosome biogenesis normalized by the hypothetical reference gene (FPKM =100 for all 158 samples, (CV = 0%). **Figure S7c**. Ribosome biogenesis normalized by the *C1orf43* (CV = 16%). **Figure S7d**. Ribosome biogenesis normalized by the *RAB7A* (CV = 17%). **Figure S7e**. Ribosome biogenesis normalized by the *HEBP2* (CV = 21%). **Figure S7 f**. Ribosome biogenesis normalized by the *ACTB* (CV = 34%). **Figure S7 g.** Ribosome biogenesis normalized by the *TFRC* (CV = 52%). **Figure S7 h.** Ribosome biogenesis normalized by the *HSP90AB1* (CV = 75%). **Figure S8.** Weak agreement of 3 ribosome biogenesis gene expression profiles between NGS and qPCR in 20 CRC pairs. **Figure S8a1**. Comparison of NGS and qPCR of BOP1. **Figure S8a2**. Comparison of NGS and qPCR of DKC1. **Figure S8a3.** Comparison of NGS and qPCR of RRP1B. **Figure S8b1**. Correlation of NGS and qPCR of BOP1. **Figure S8b2**. Correlation of NGS and qPCR of DKC1. **Figure S8b3**. Correlation of NGS and qPCR of RRP1B. **Figure S9**. NGS analysis of 6 cancer hallmark genes expression in 50 CRCs (TCGA). **Figure S9a**. Upregulation of CCND1, CDK4 and MYC in 50 CRCs. **Figure S9b**. Downregulation of AQP8, GPX3 and CD177 in 50 CRCs. **Figure S10.** Co-downregulation of 3 reference genes with 3 tumor suppressors in 3 out of 6 H/SNs detected by PCA. (PPTX 3420 kb)

## Data Availability

The datasets used and/or analyzed during the current study available from the corresponding author on reasonable request.

## Abbreviations

CRC: Colorectal cancer
CV: Coefficients of variation
FPKM: Fragments per kilobase of transcript per million mapped reads
HCA: Hierarchical clustering analyses
HSN: Normal tissue adjacent to high stage tumor
HST: High stage tumor
LN: Lymph node
LSN: Normal tissue adjacent to low stage tumor
LST: Low stage tumor
LV: Lymphatic vessel
N: Normal sample
NGS: Next generation sequencing
PCA: Principal component analyses
qPCR: Quantitative PCR
RG: Reference gene
RGCOEX: Reference gene coexpressed gene
T: Tumor sample
