# Supplementary material for: Novel reference genes in colorectal cancer identify a distinct subset of high stage tumors and their associated histologically normal colonic tissues
Source: BMC Med Genet. 2019 Aug 13;20:138. doi: 10.1186/s12881-019-0867-y (PMC6693228; doi:10.1186/s12881-019-0867-y)
Supplement: Supplementary file 1 — Table S1. Clinical information of 79 CRC pairs. The MSI and MSS information were available for 8 tumors. Table S2. Tumor information of 50 CRC pairs from TCGA. Table S3. 21 classical reference genes and their annotated functions. Table S4. Expression profiles of 21 classical reference genes in 79 CRC cohort. Table S5. Expression profiles of 42 colorectal reference genes in 79 CRC cohort. Table S6. 23 reference genes with the annotated functions of cellular cargo transportation. Table S7. 13 reference genes with the annotated functions of structural proteins. Table S8. 9 reference genes with the annotated functions of enzymes. Table S9. Determination of possible function of 8 reference genes through correlation analysis. Table S10. Identification of 8 RGCOEX genes based on their correlation with 5 novel reference genes. Table S11. Genes coexpressed with 6 reference genes. Table S12. Expression profiles of 8 RGCOEX genes. Table S13. Expression profiles of classical 21 reference genes in 50 CRCs (TCGA). Table S14. Expression profiles of novel colorectal 42 reference genes in 50 CRC pairs (TCGA). Table S15. Oncogenes and tumor suppressors coexpressed with 3 reference genes (RAB1B, ACTR2 and CLTC). Figure S1. NGS analysis of 15 genes pertaining to ribosome biogenesis in 79 CRCs. Figure S1a. Upregulation of 15 genes pertaining to ribosome biogenesis in 79 CRCs. These 15ribosome biogenesis related genes were identified from 1223 upregulated genes (average T/N > 2 fold, FDR < 0.05 (ANOVA)) by DAVID Bioinformatics Resources 6.8 (https://david.ncifcrf.gov/) [11]. The 15 ribosome biogenesis genes are: D-Tyrosyl-TRNA Deacylase 1 (DTD1), Dyskerin Pseudouridine Synthase 1 (DKC1), GTP Binding Protein 4 (GTPBP4), Ribosomal RNA Processing 1B (RRP1B), Block Of Proliferation 1 (BOP1), DDB1 and CUL4 Associated Factor 13 (DCAF13), Nucleolar Protein (NOP2), Ribosomal RNA processing protein 1 (RRP1), Nucleolar And Coiled-Body Phosphoprotein 1 (NOLC1), Nucleophosmin 1(NPM1), Biogenesis Of [file 12881_2019_867_MOESM1_ESM.pptx]

## Slide 1
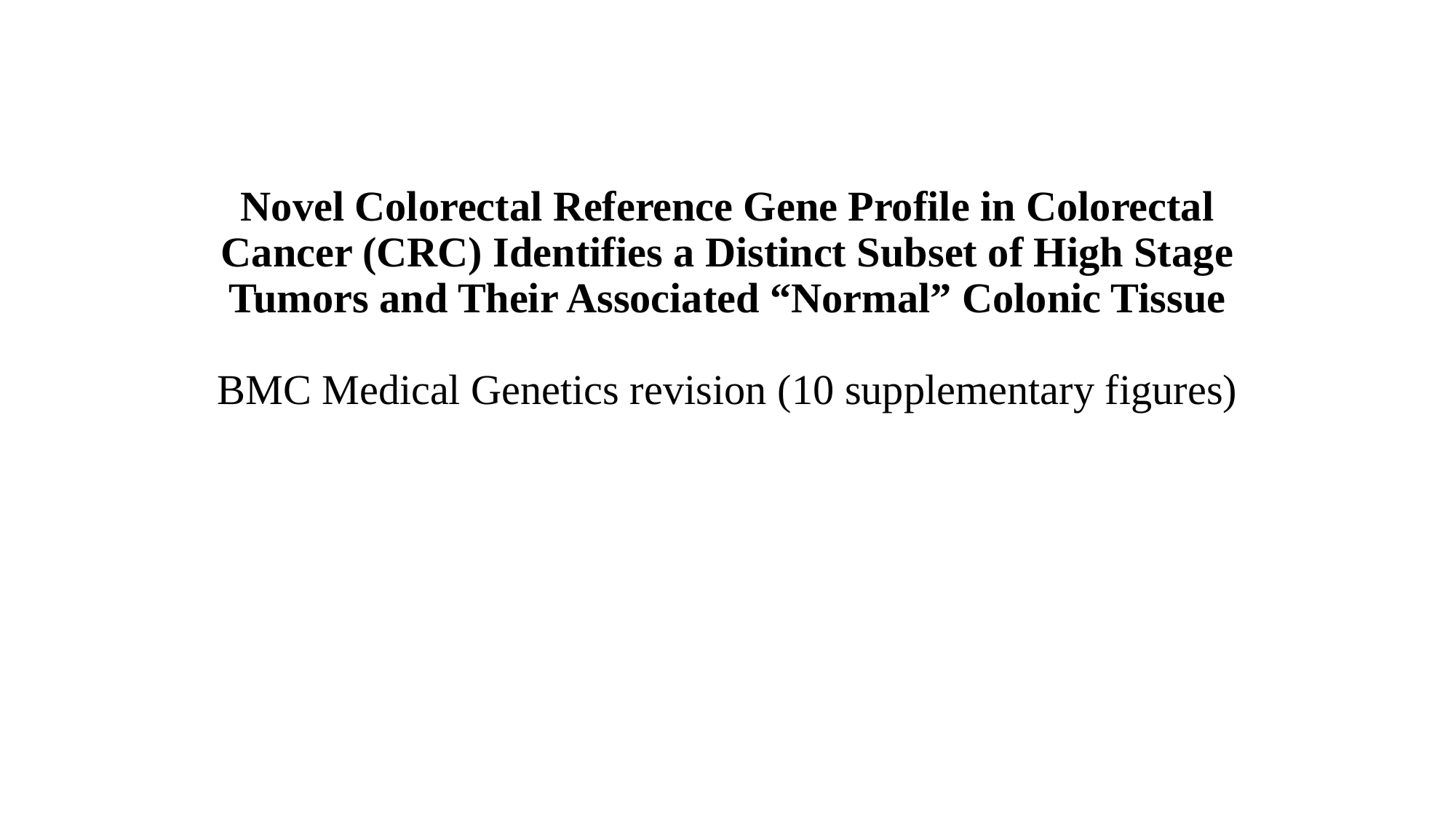

# Novel Colorectal Reference Gene Profile in Colorectal Cancer (CRC) Identifies a Distinct Subset of High Stage Tumors and Their Associated “Normal” Colonic Tissue BMC Medical Genetics revision (10 supplementary figures)

## Slide 2
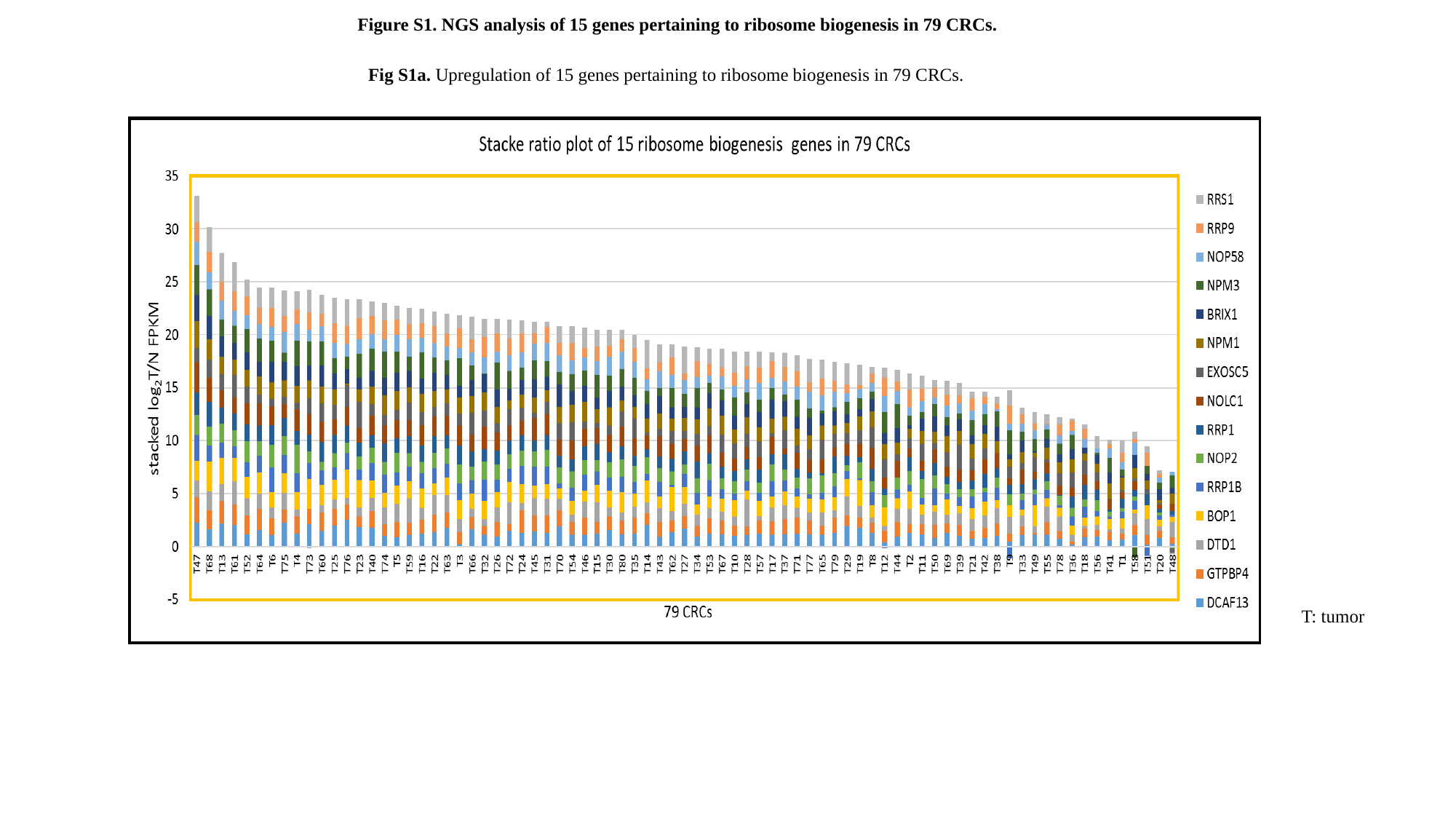

Figure S1. NGS analysis of 15 genes pertaining to ribosome biogenesis in 79 CRCs.
Fig S1a. Upregulation of 15 genes pertaining to ribosome biogenesis in 79 CRCs.
T: tumor

## Slide 3
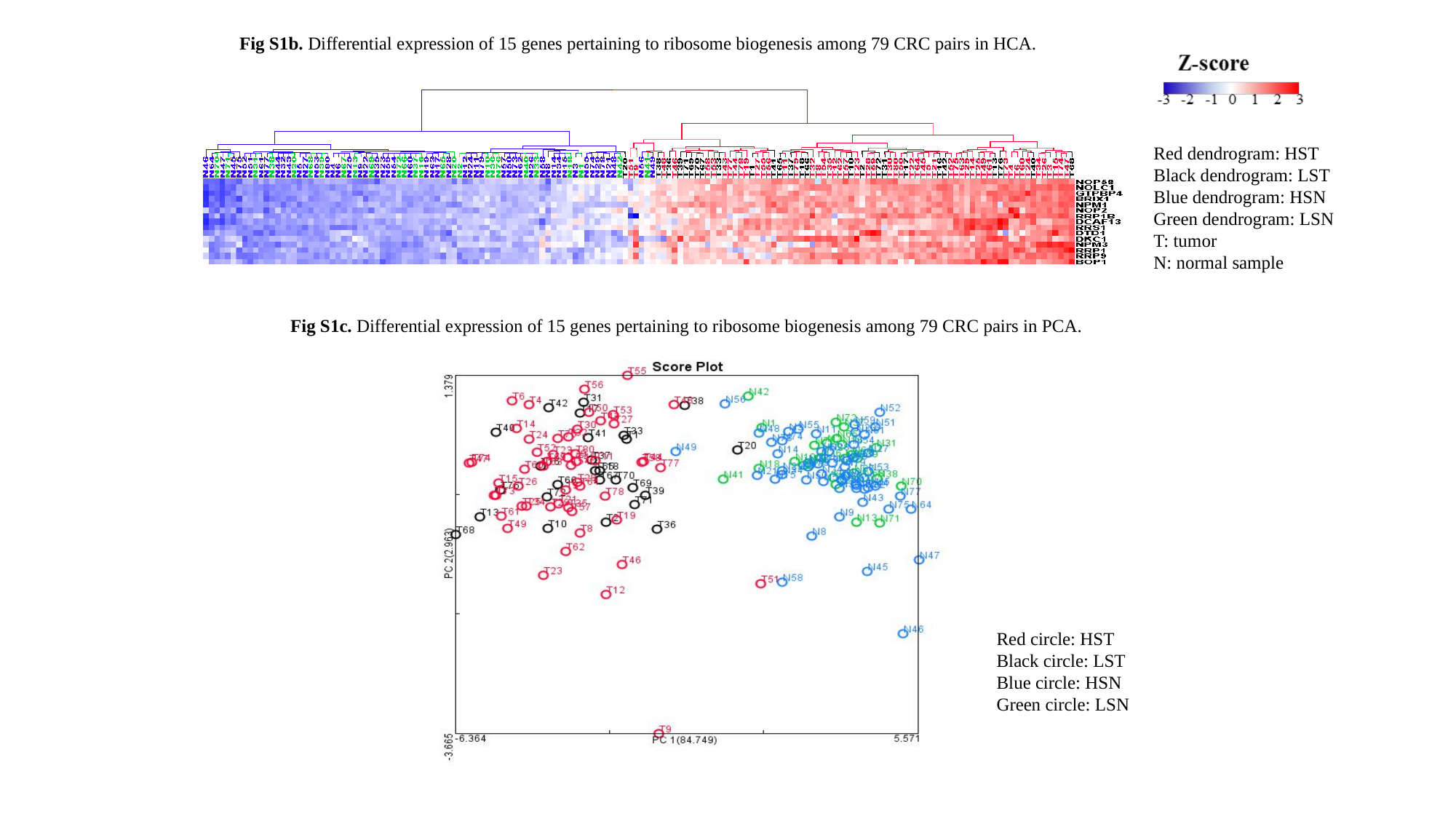

Fig S1b. Differential expression of 15 genes pertaining to ribosome biogenesis among 79 CRC pairs in HCA.
Red dendrogram: HST
Black dendrogram: LST
Blue dendrogram: HSN
Green dendrogram: LSN
T: tumor
N: normal sample
Fig S1c. Differential expression of 15 genes pertaining to ribosome biogenesis among 79 CRC pairs in PCA.
Red circle: HST
Black circle: LST
Blue circle: HSN
Green circle: LSN

## Slide 4
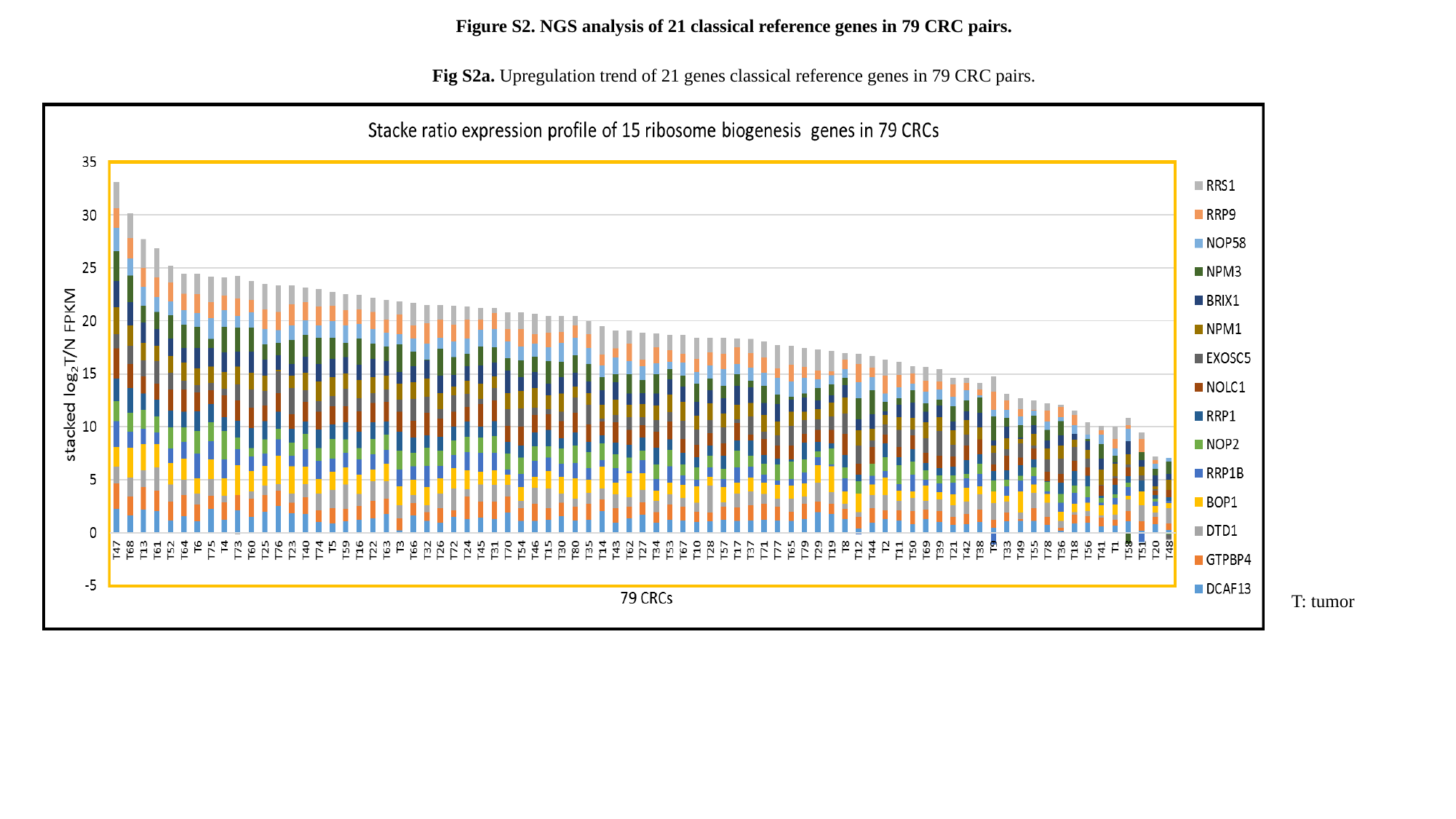

Figure S2. NGS analysis of 21 classical reference genes in 79 CRC pairs.
Fig S2a. Upregulation trend of 21 genes classical reference genes in 79 CRC pairs.
T: tumor

## Slide 5
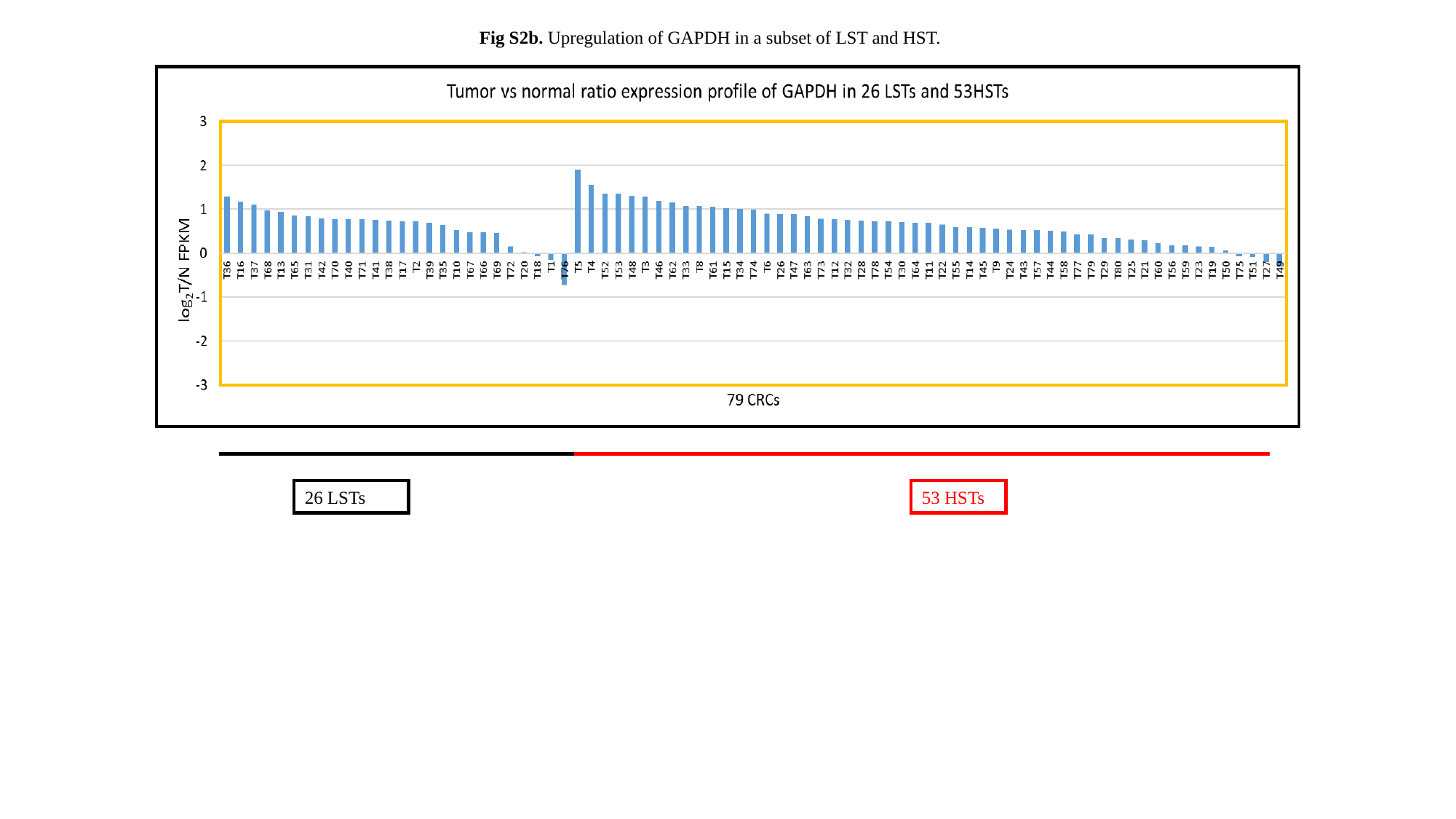

Fig S2b. Upregulation of GAPDH in a subset of LST and HST.
26 LSTs
53 HSTs

## Slide 6
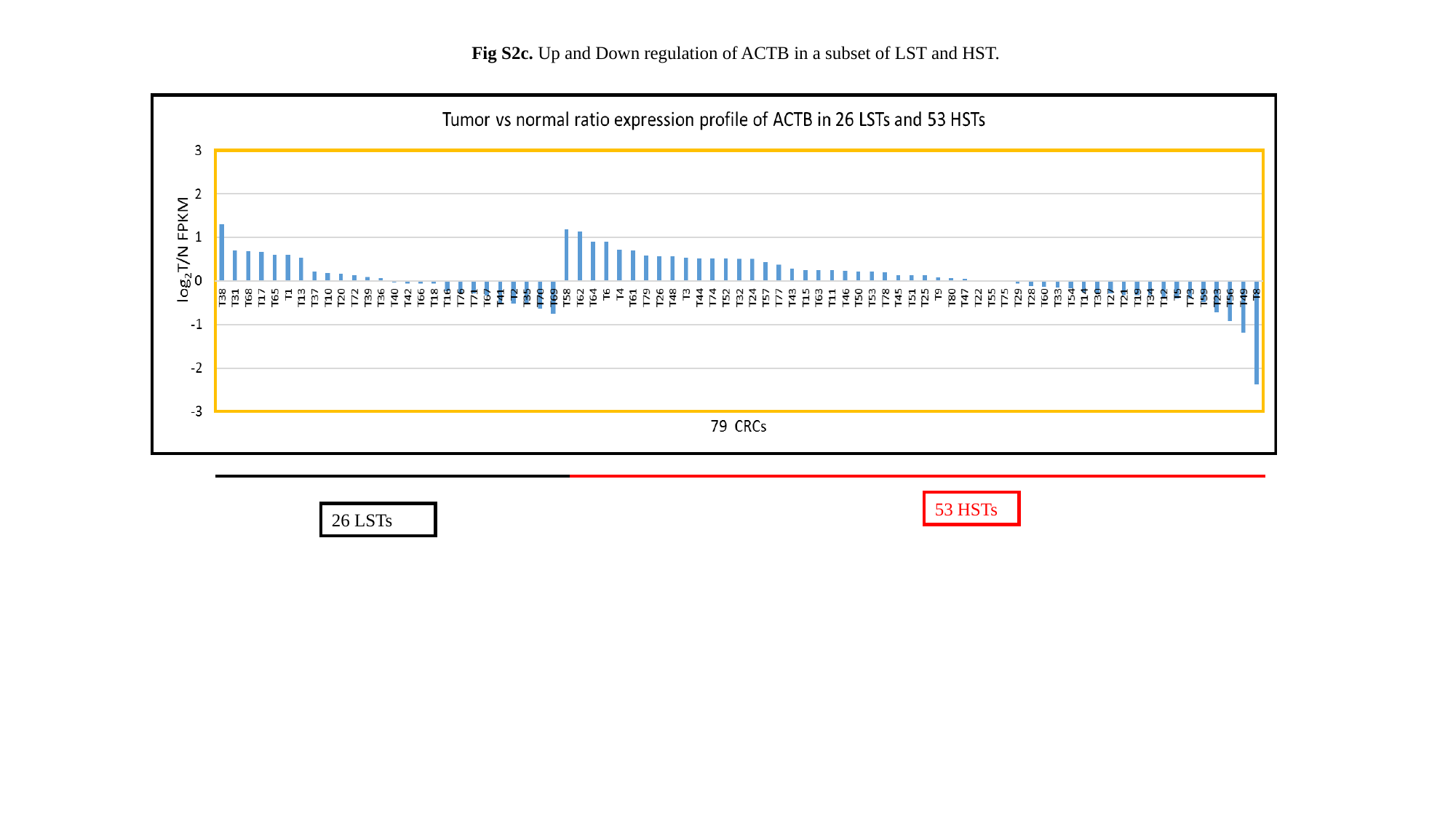

Fig S2c. Up and Down regulation of ACTB in a subset of LST and HST.
53 HSTs
26 LSTs

## Slide 7
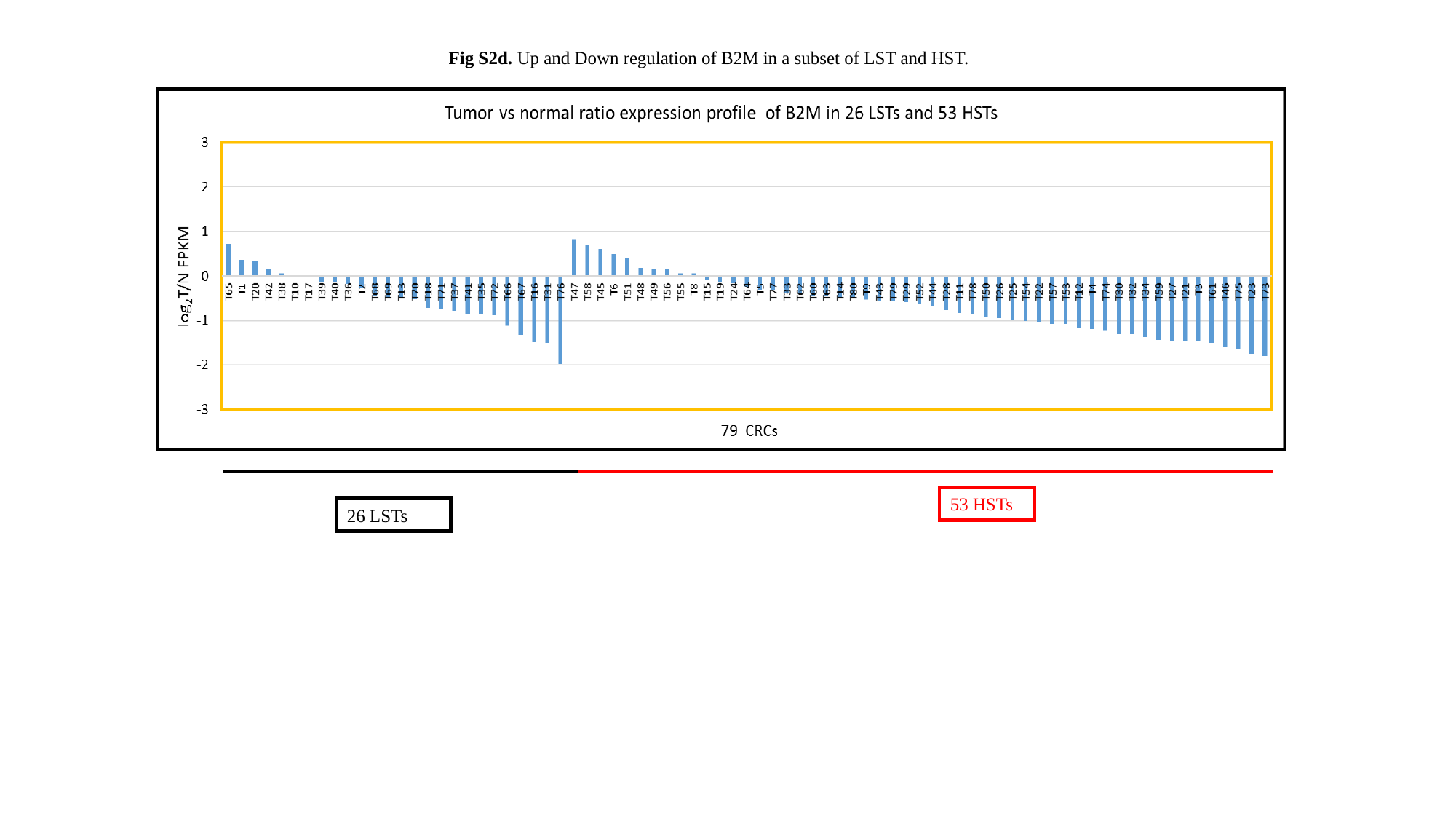

Fig S2d. Up and Down regulation of B2M in a subset of LST and HST.
53 HSTs
26 LSTs

## Slide 8
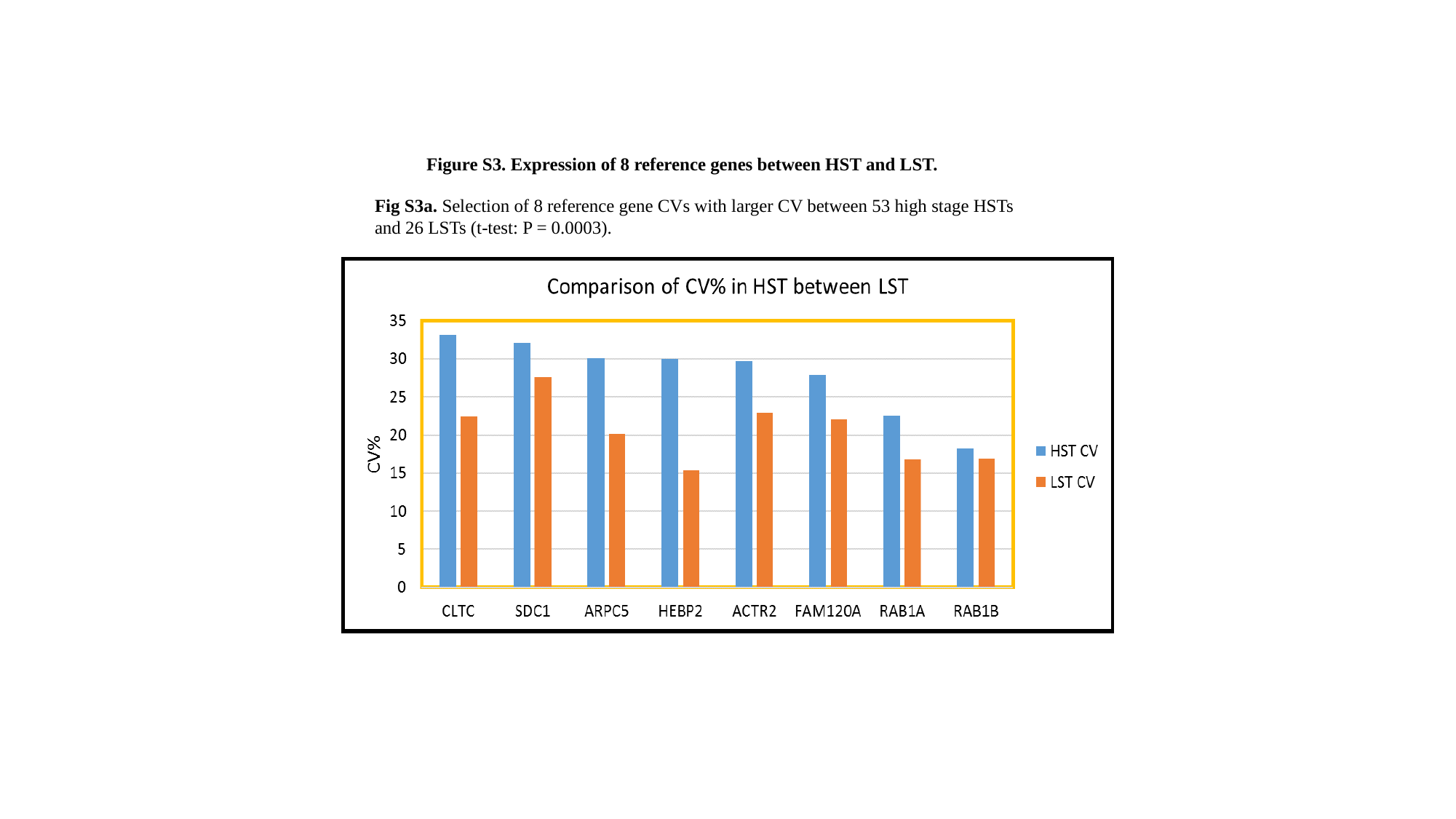

Figure S3. Expression of 8 reference genes between HST and LST.
Fig S3a. Selection of 8 reference gene CVs with larger CV between 53 high stage HSTs and 26 LSTs (t-test: P = 0.0003).

## Slide 9
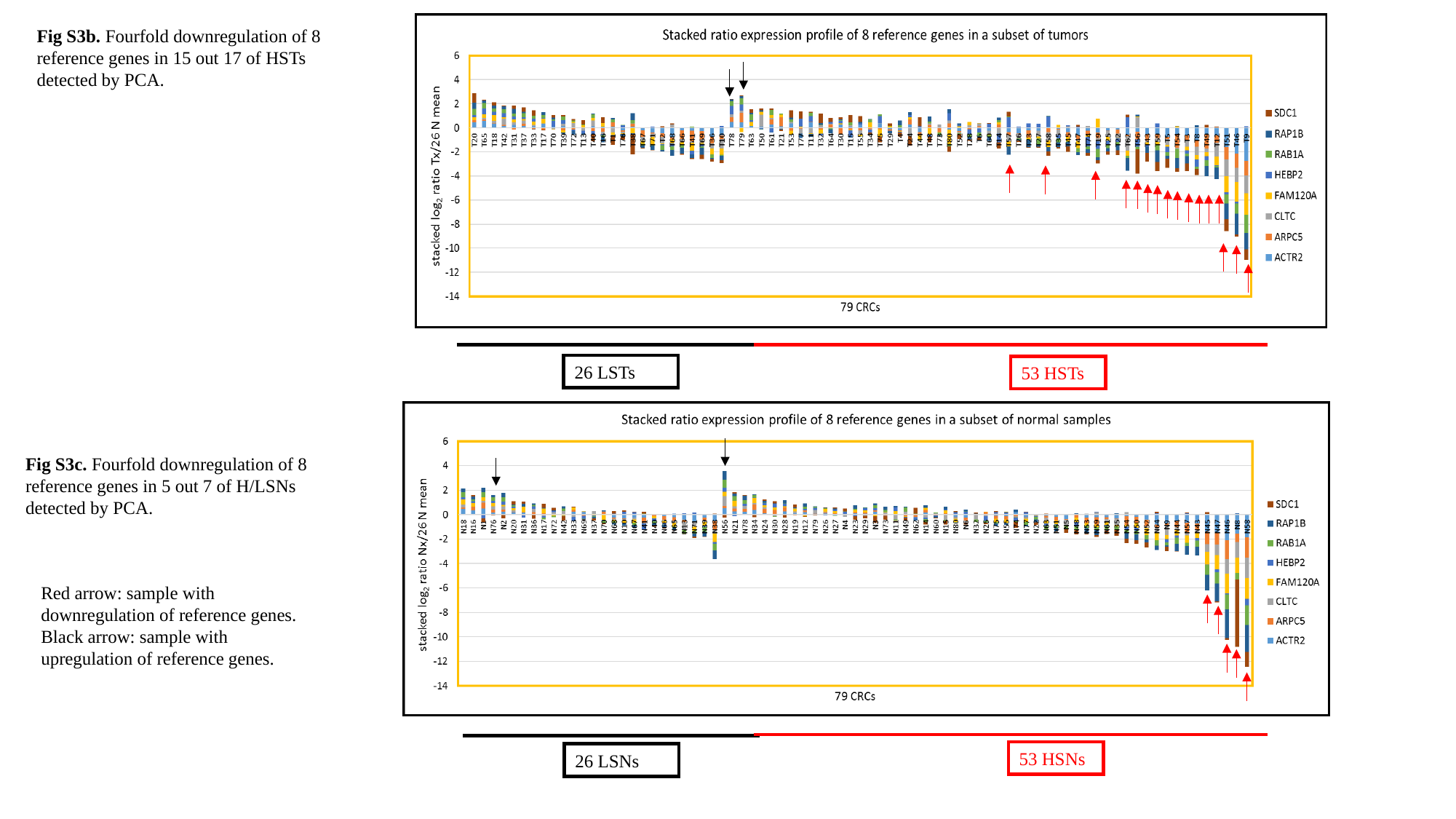

Fig S3b. Fourfold downregulation of 8 reference genes in 15 out 17 of HSTs detected by PCA.
26 LSTs
53 HSTs
Fig S3c. Fourfold downregulation of 8 reference genes in 5 out 7 of H/LSNs detected by PCA.
Red arrow: sample with downregulation of reference genes.
Black arrow: sample with upregulation of reference genes.
53 HSNs
26 LSNs

## Slide 10
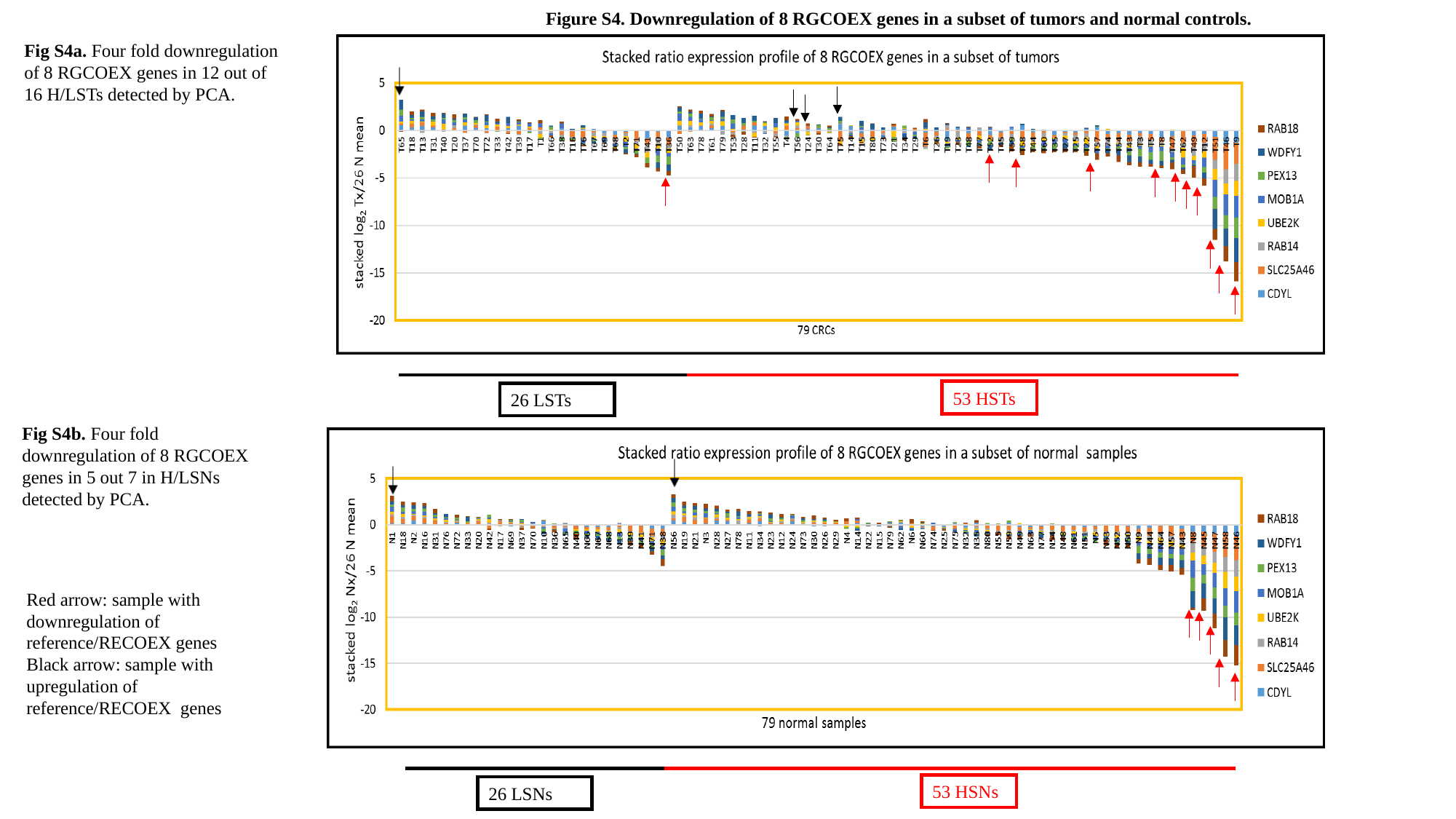

Figure S4. Downregulation of 8 RGCOEX genes in a subset of tumors and normal controls.
Fig S4a. Four fold downregulation of 8 RGCOEX genes in 12 out of 16 H/LSTs detected by PCA.
53 HSTs
26 LSTs
Fig S4b. Four fold downregulation of 8 RGCOEX genes in 5 out 7 in H/LSNs detected by PCA.
Red arrow: sample with downregulation of reference/RECOEX genes
Black arrow: sample with upregulation of reference/RECOEX genes
53 HSNs
26 LSNs

## Slide 11
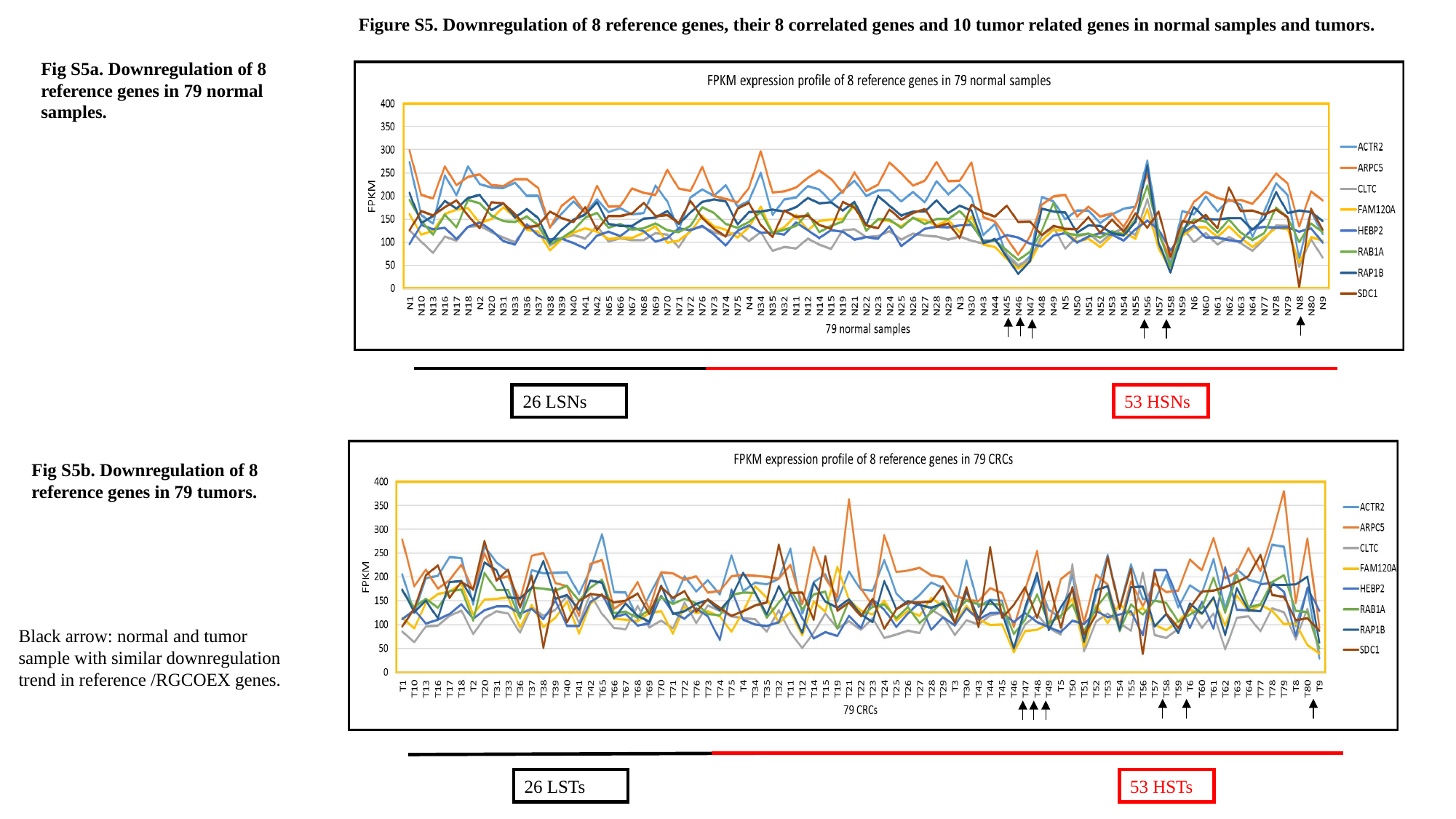

Figure S5. Downregulation of 8 reference genes, their 8 correlated genes and 10 tumor related genes in normal samples and tumors.
Fig S5a. Downregulation of 8 reference genes in 79 normal samples.
26 LSNs
53 HSNs
Fig S5b. Downregulation of 8 reference genes in 79 tumors.
Black arrow: normal and tumor sample with similar downregulation trend in reference /RGCOEX genes.
53 HSTs
26 LSTs

## Slide 12
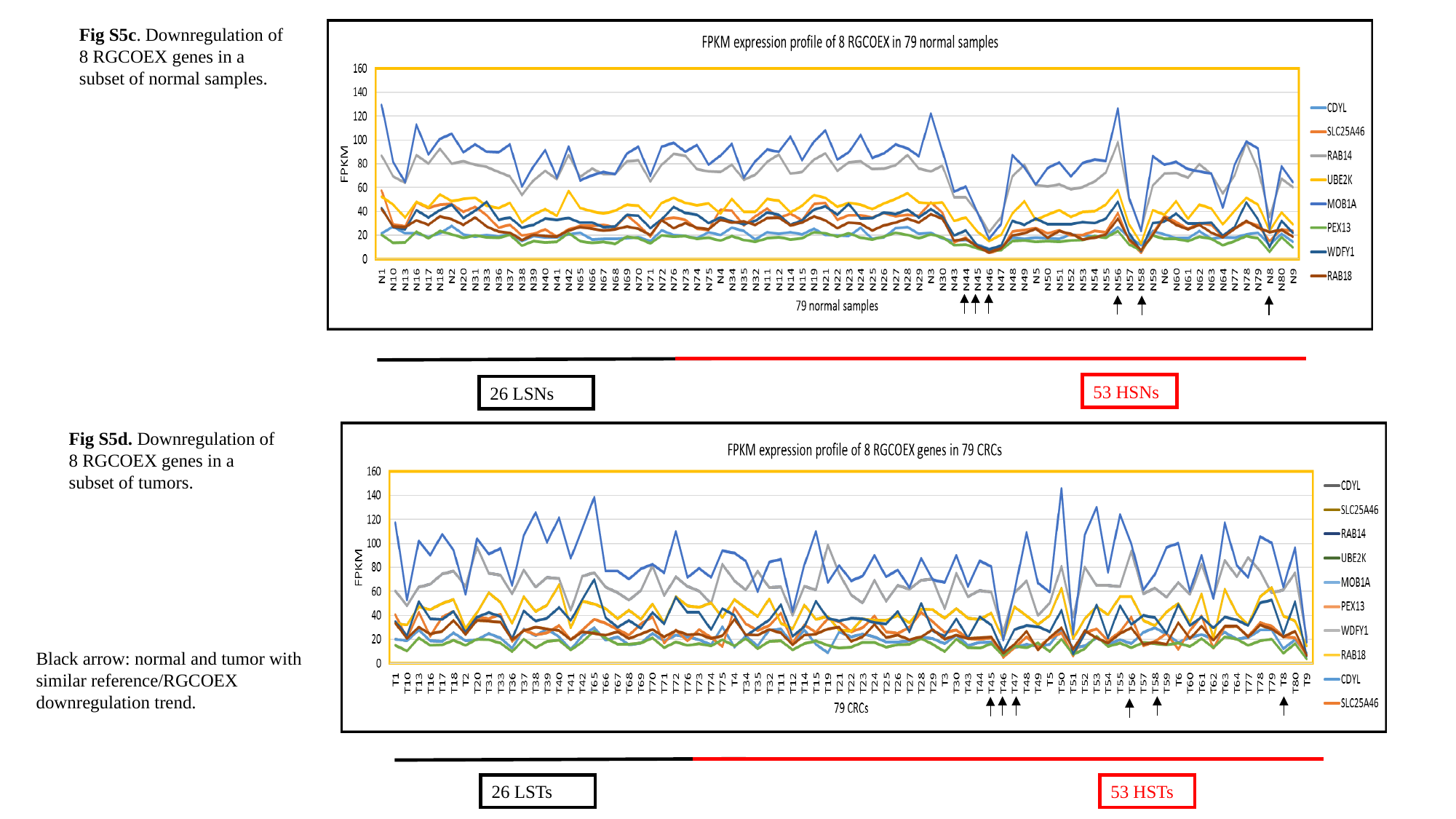

Fig S5c. Downregulation of 8 RGCOEX genes in a subset of normal samples.
53 HSNs
26 LSNs
Fig S5d. Downregulation of 8 RGCOEX genes in a subset of tumors.
Black arrow: normal and tumor with similar reference/RGCOEX downregulation trend.
26 LSTs
53 HSTs

## Slide 13
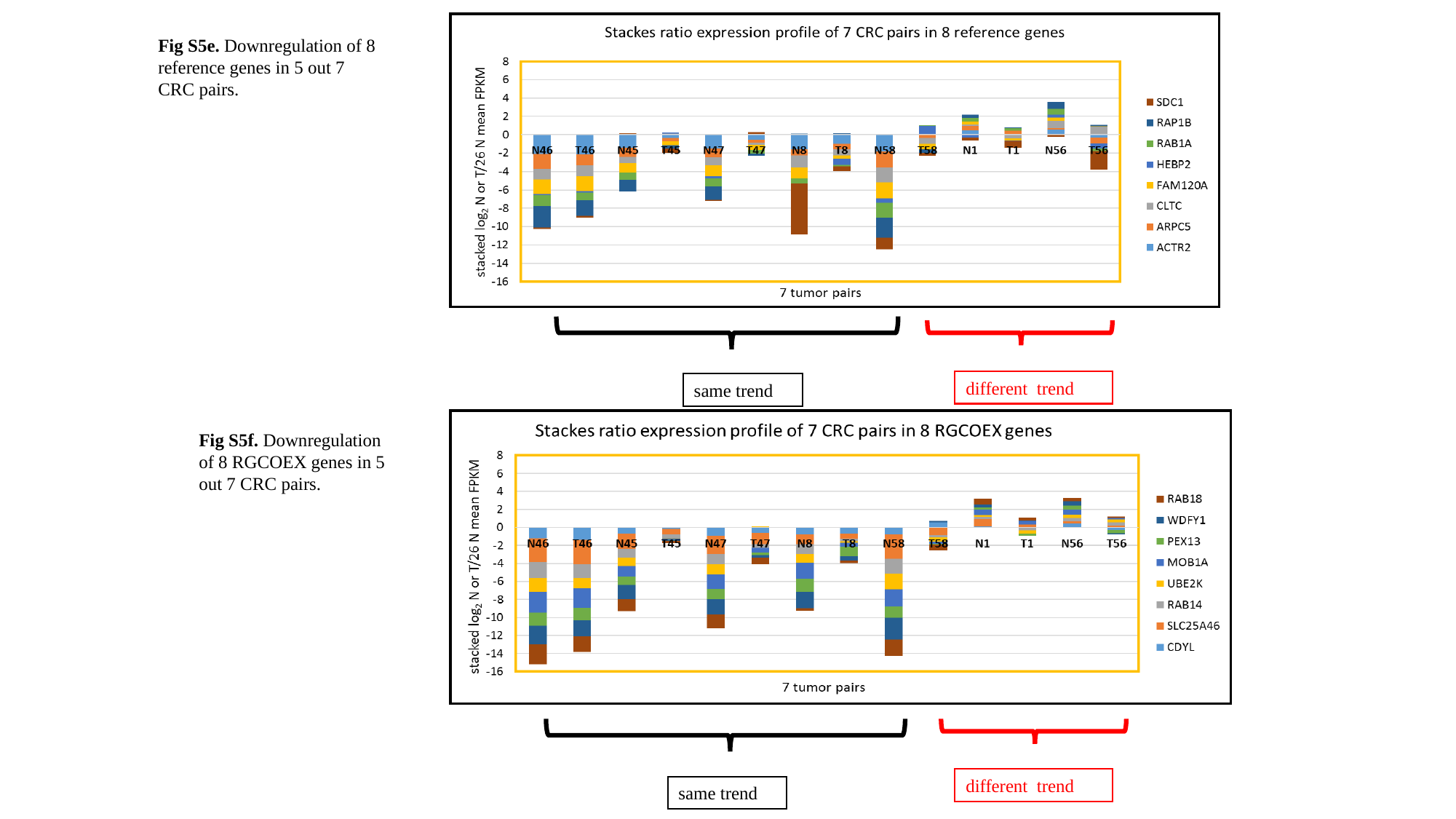

Fig S5e. Downregulation of 8 reference genes in 5 out 7 CRC pairs.
different trend
same trend
Fig S5f. Downregulation of 8 RGCOEX genes in 5 out 7 CRC pairs.
different trend
same trend

## Slide 14
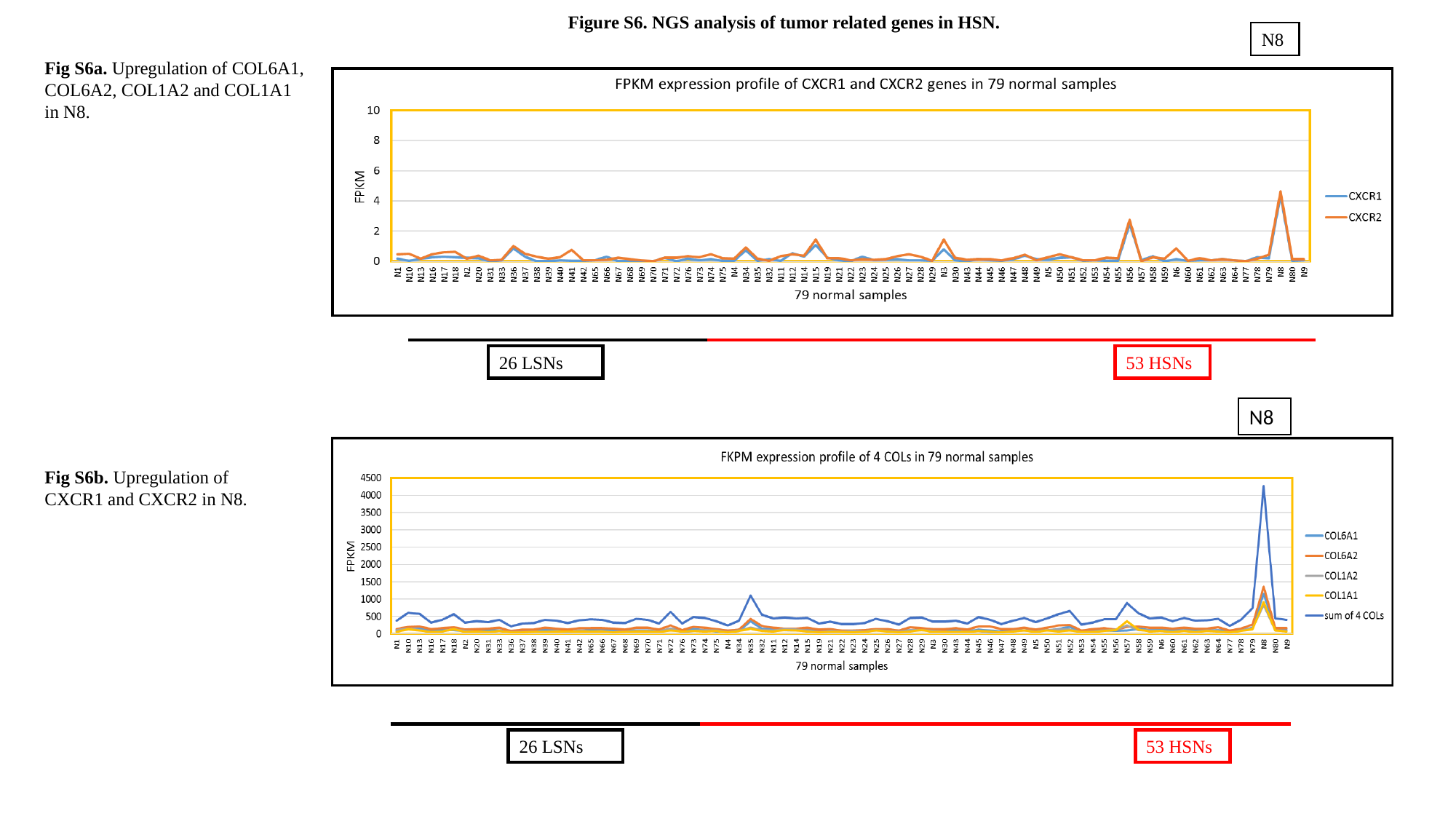

Figure S6. NGS analysis of tumor related genes in HSN.
N8
Fig S6a. Upregulation of COL6A1, COL6A2, COL1A2 and COL1A1 in N8.
26 LSNs
53 HSNs
N8
Fig S6b. Upregulation of CXCR1 and CXCR2 in N8.
26 LSNs
53 HSNs

## Slide 15
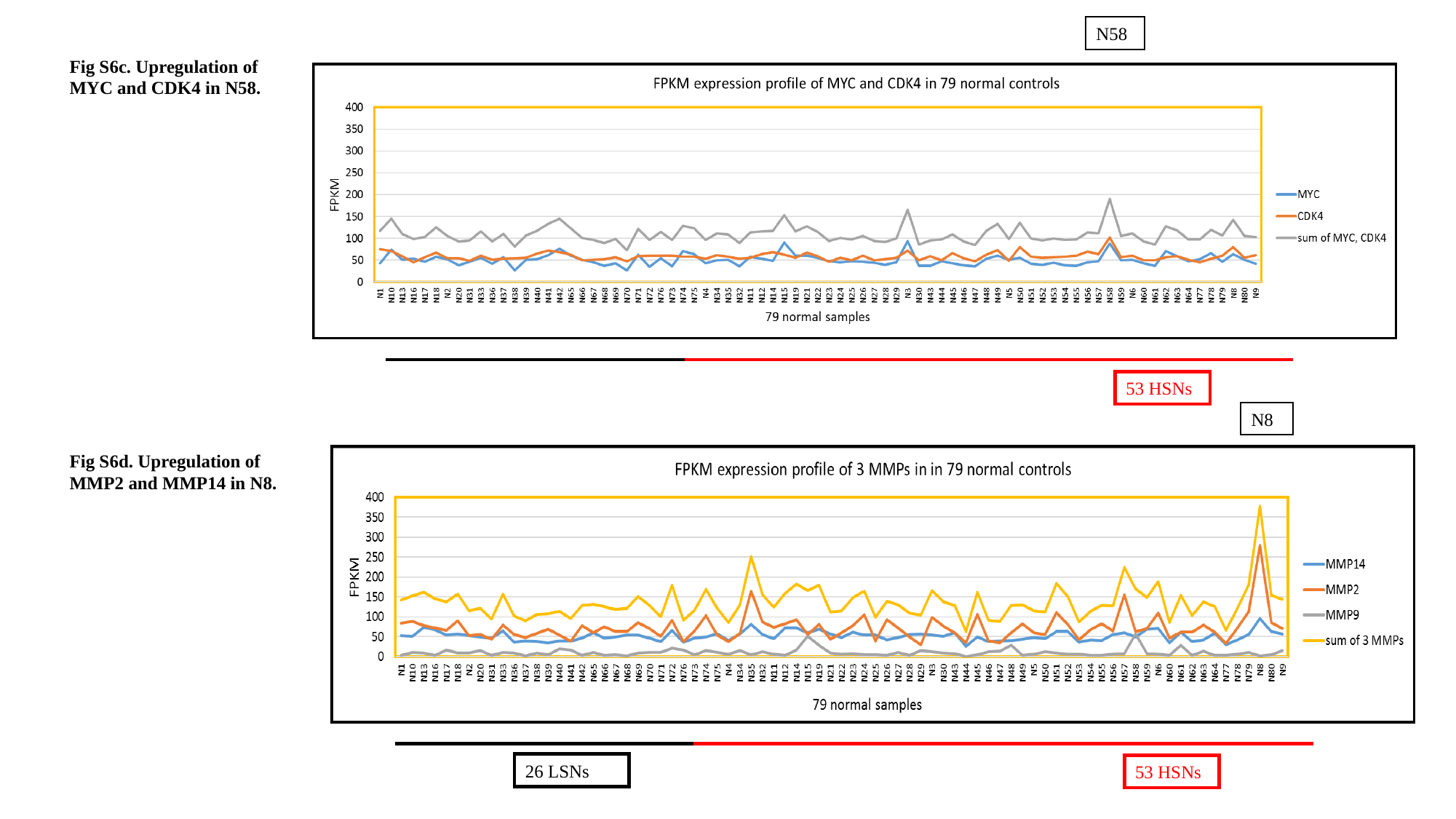

N58
Fig S6c. Upregulation of MYC and CDK4 in N58.
53 HSNs
N8
Fig S6d. Upregulation of MMP2 and MMP14 in N8.
26 LSNs
53 HSNs

## Slide 16
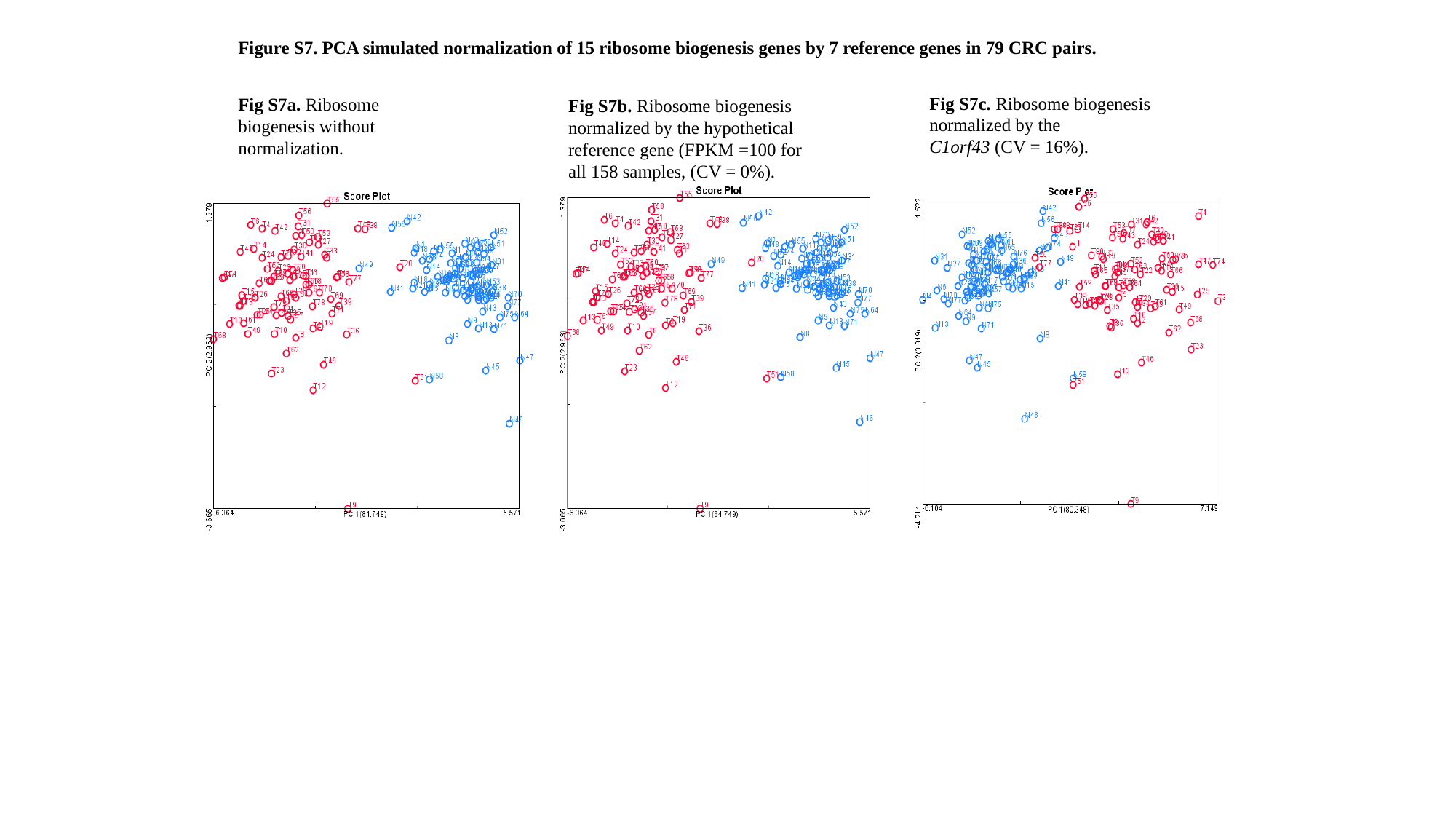

Figure S7. PCA simulated normalization of 15 ribosome biogenesis genes by 7 reference genes in 79 CRC pairs.
Fig S7c. Ribosome biogenesis normalized by the
C1orf43 (CV = 16%).
Fig S7a. Ribosome biogenesis without normalization.
Fig S7b. Ribosome biogenesis normalized by the hypothetical reference gene (FPKM =100 for all 158 samples, (CV = 0%).

## Slide 17
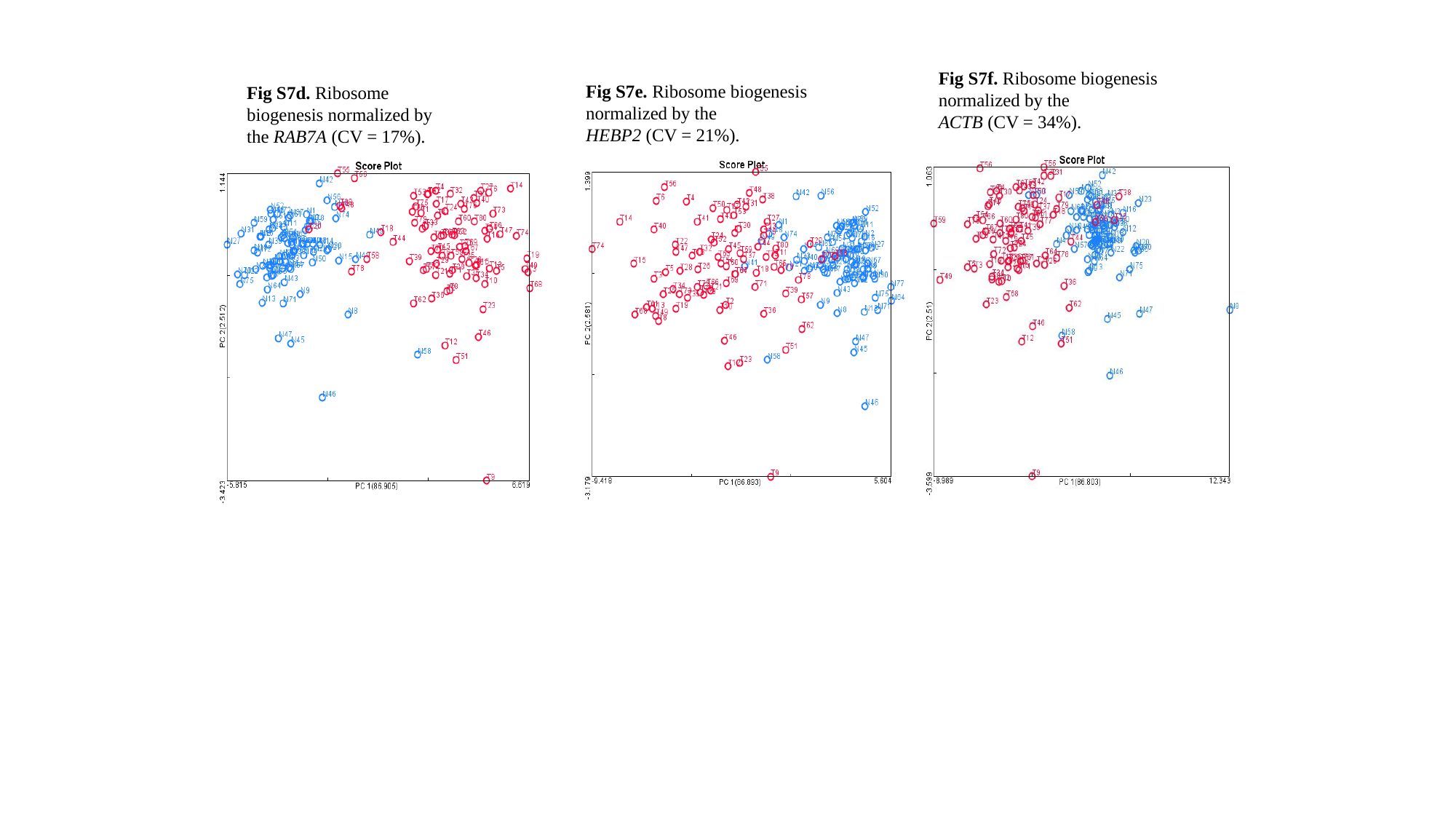

Fig S7f. Ribosome biogenesis normalized by the
ACTB (CV = 34%).
Fig S7e. Ribosome biogenesis normalized by the
HEBP2 (CV = 21%).
Fig S7d. Ribosome biogenesis normalized by the RAB7A (CV = 17%).

## Slide 18
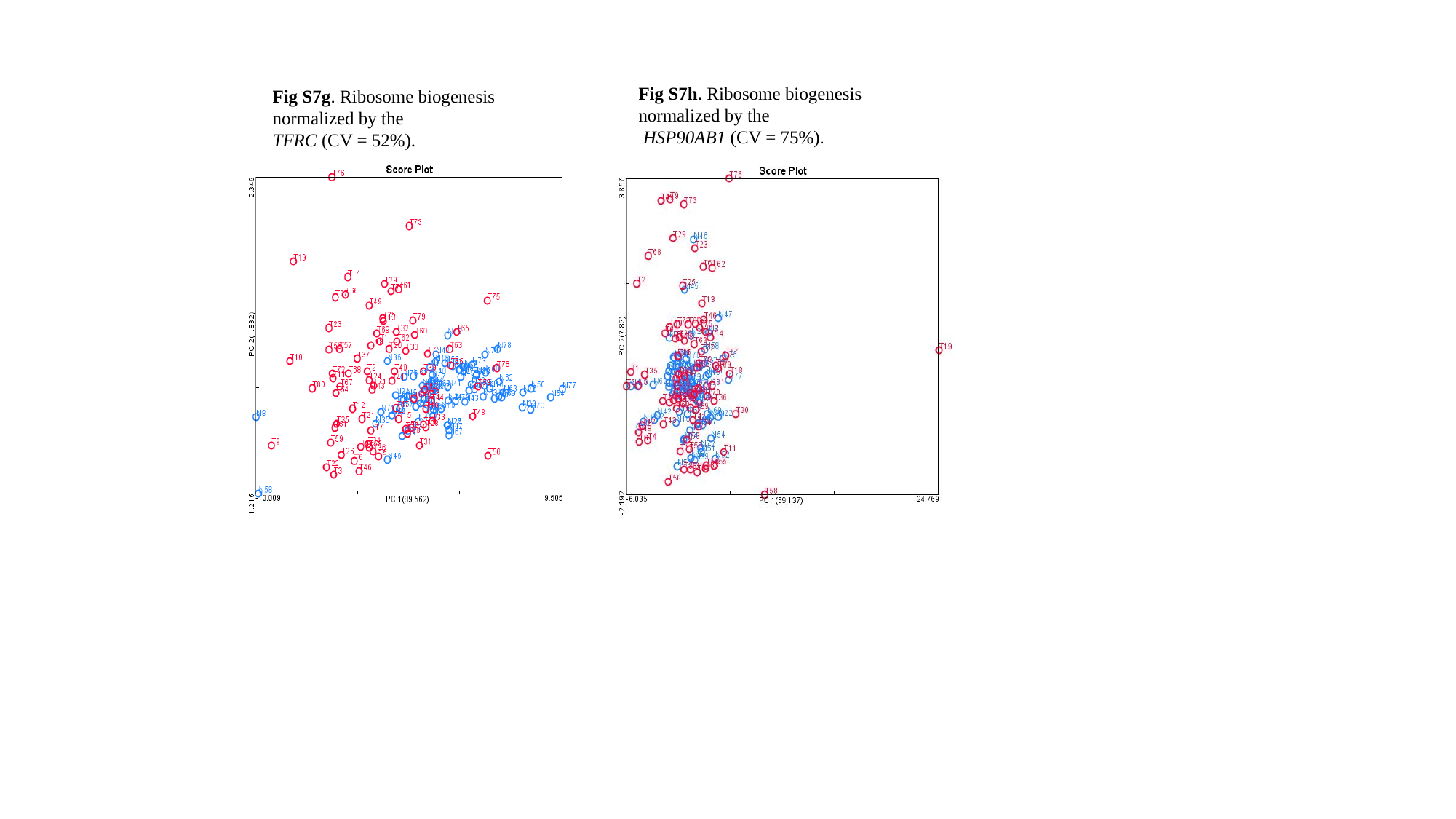

Fig S7h. Ribosome biogenesis normalized by the
 HSP90AB1 (CV = 75%).
Fig S7g. Ribosome biogenesis normalized by the
TFRC (CV = 52%).

## Slide 19
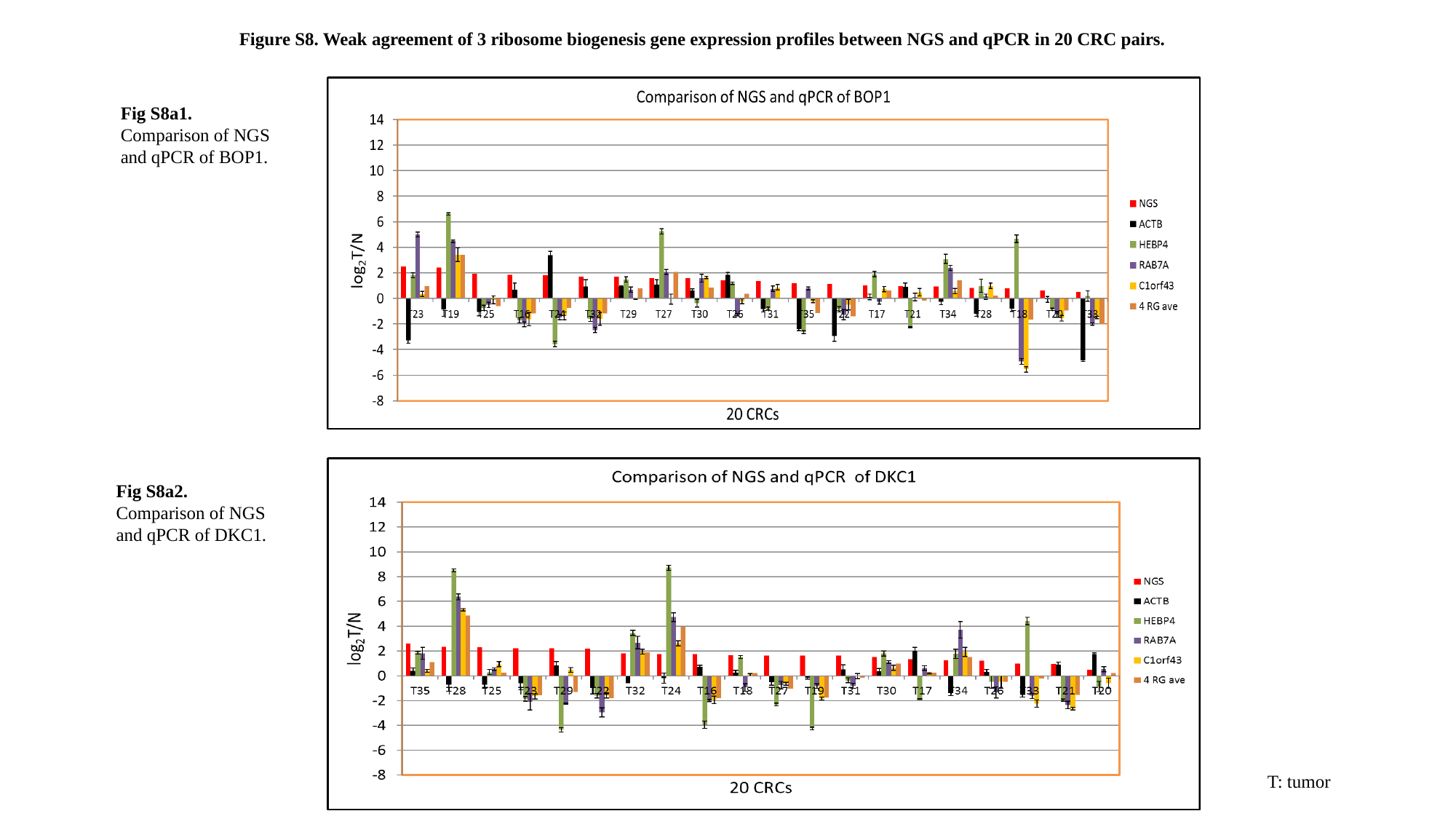

Figure S8. Weak agreement of 3 ribosome biogenesis gene expression profiles between NGS and qPCR in 20 CRC pairs.
Fig S8a1. Comparison of NGS and qPCR of BOP1.
Fig S8a2.
Comparison of NGS and qPCR of DKC1.
T: tumor

## Slide 20
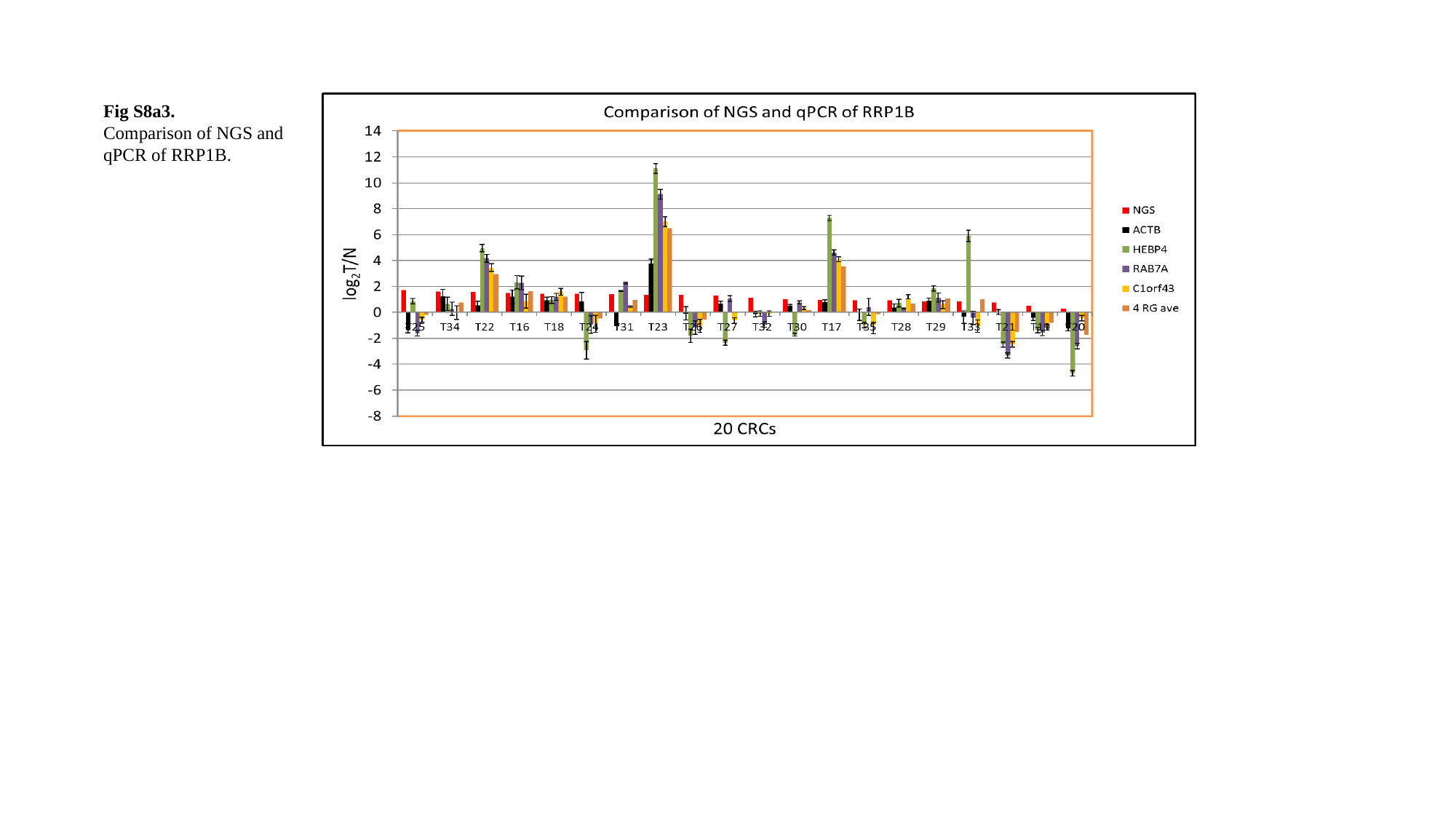

Fig S8a3.
Comparison of NGS and qPCR of RRP1B.

## Slide 21
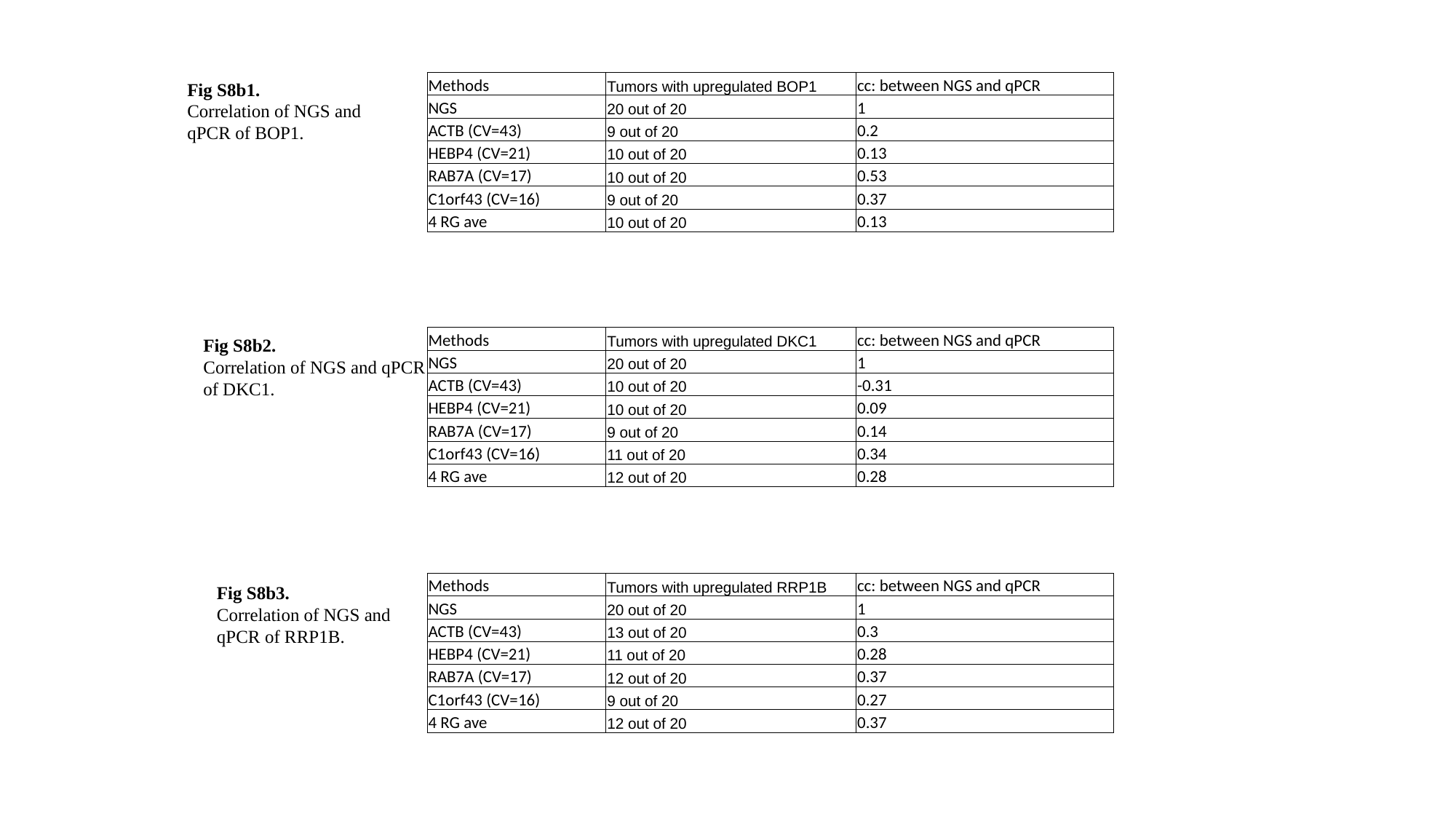

Fig S8b1.
Correlation of NGS and qPCR of BOP1.
| Methods | Tumors with upregulated BOP1 | cc: between NGS and qPCR |
| --- | --- | --- |
| NGS | 20 out of 20 | 1 |
| ACTB (CV=43) | 9 out of 20 | 0.2 |
| HEBP4 (CV=21) | 10 out of 20 | 0.13 |
| RAB7A (CV=17) | 10 out of 20 | 0.53 |
| C1orf43 (CV=16) | 9 out of 20 | 0.37 |
| 4 RG ave | 10 out of 20 | 0.13 |
| Methods | Tumors with upregulated DKC1 | cc: between NGS and qPCR |
| --- | --- | --- |
| NGS | 20 out of 20 | 1 |
| ACTB (CV=43) | 10 out of 20 | -0.31 |
| HEBP4 (CV=21) | 10 out of 20 | 0.09 |
| RAB7A (CV=17) | 9 out of 20 | 0.14 |
| C1orf43 (CV=16) | 11 out of 20 | 0.34 |
| 4 RG ave | 12 out of 20 | 0.28 |
Fig S8b2.
Correlation of NGS and qPCR of DKC1.
| Methods | Tumors with upregulated RRP1B | cc: between NGS and qPCR |
| --- | --- | --- |
| NGS | 20 out of 20 | 1 |
| ACTB (CV=43) | 13 out of 20 | 0.3 |
| HEBP4 (CV=21) | 11 out of 20 | 0.28 |
| RAB7A (CV=17) | 12 out of 20 | 0.37 |
| C1orf43 (CV=16) | 9 out of 20 | 0.27 |
| 4 RG ave | 12 out of 20 | 0.37 |
Fig S8b3.
Correlation of NGS and qPCR of RRP1B.

## Slide 22
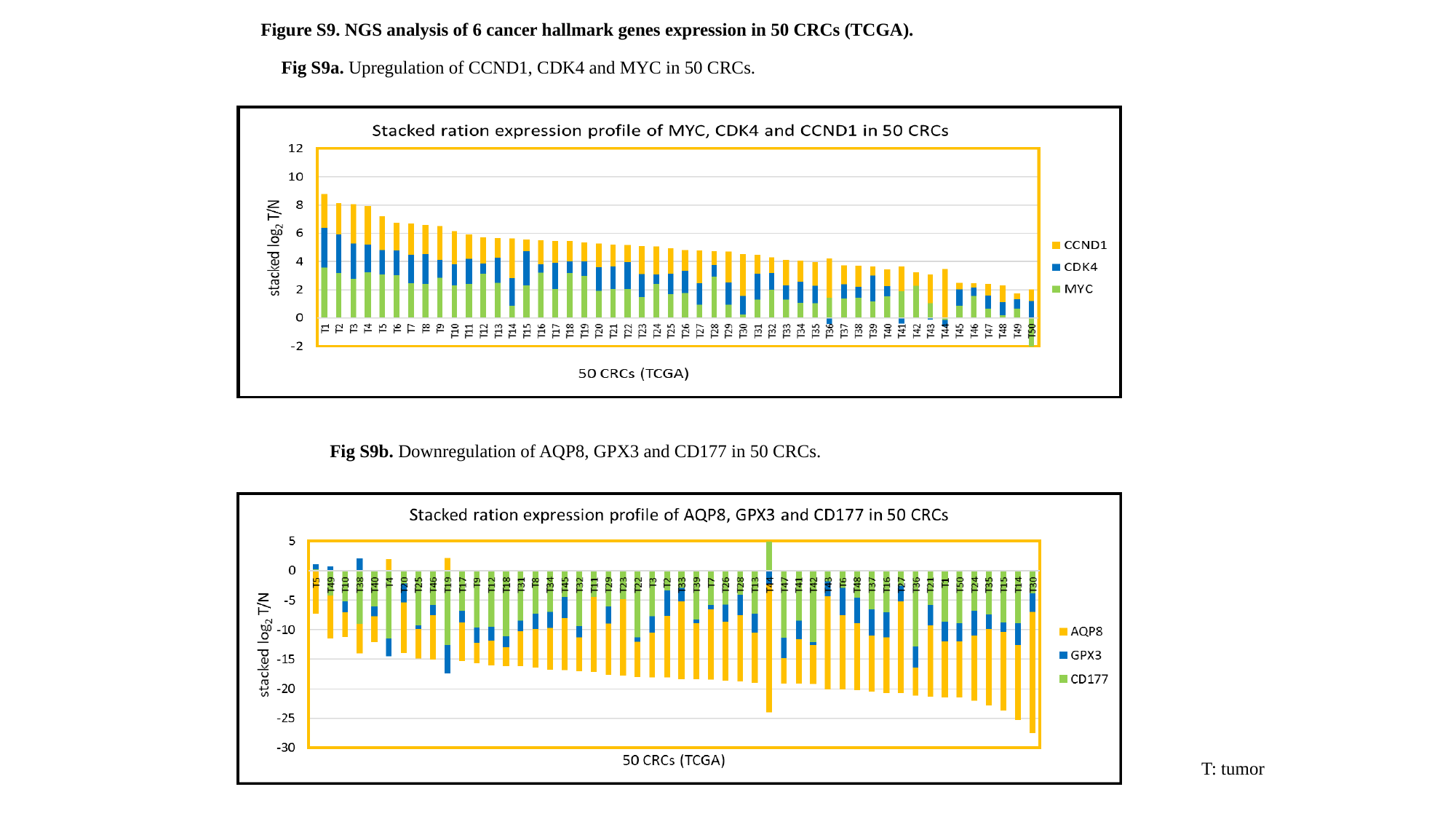

Figure S9. NGS analysis of 6 cancer hallmark genes expression in 50 CRCs (TCGA).
Fig S9a. Upregulation of CCND1, CDK4 and MYC in 50 CRCs.
Fig S9b. Downregulation of AQP8, GPX3 and CD177 in 50 CRCs.
T: tumor

## Slide 23
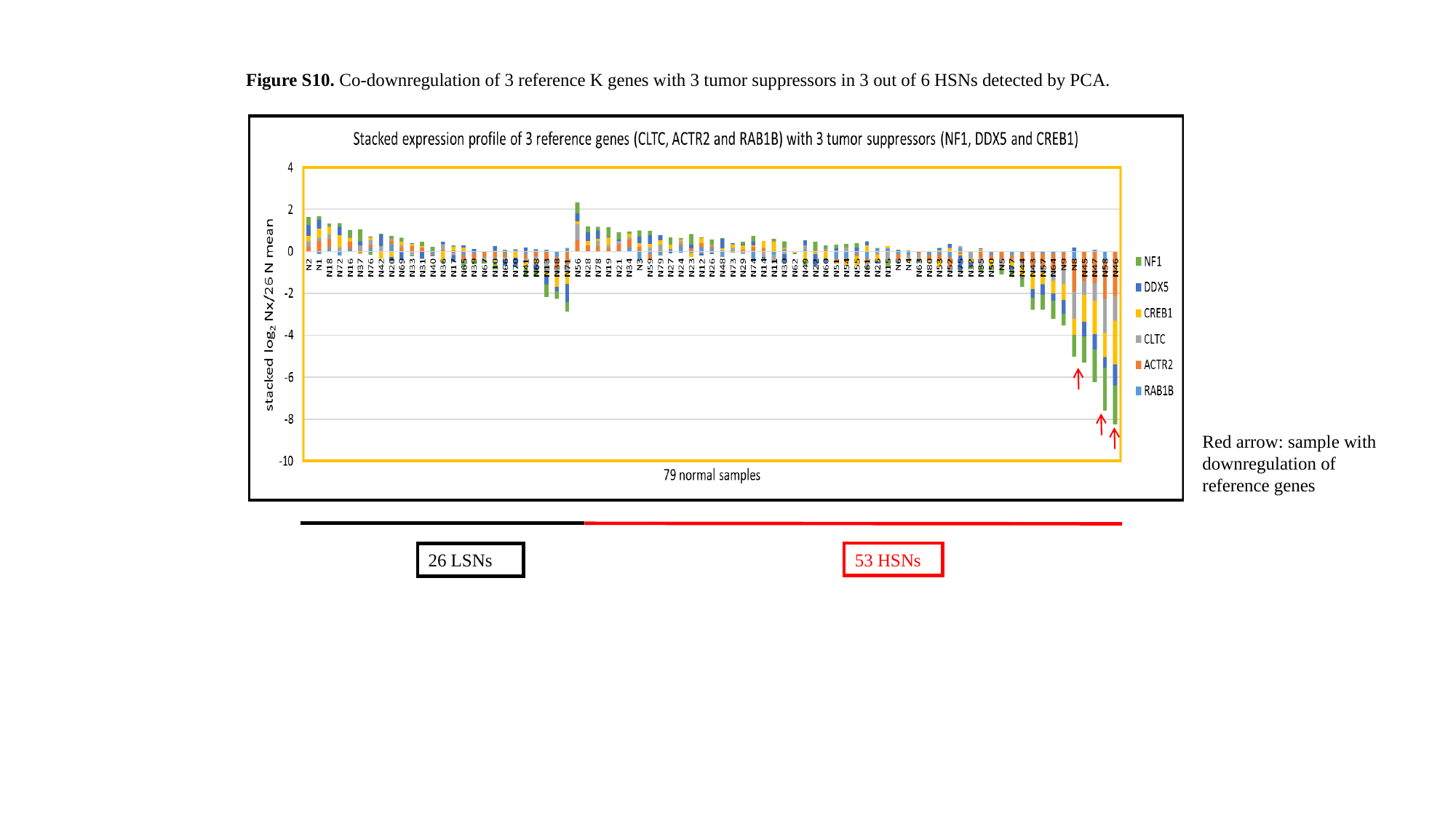

Figure S10. Co-downregulation of 3 reference K genes with 3 tumor suppressors in 3 out of 6 HSNs detected by PCA.
Red arrow: sample with downregulation of reference genes
53 HSNs
26 LSNs

## Slide 24
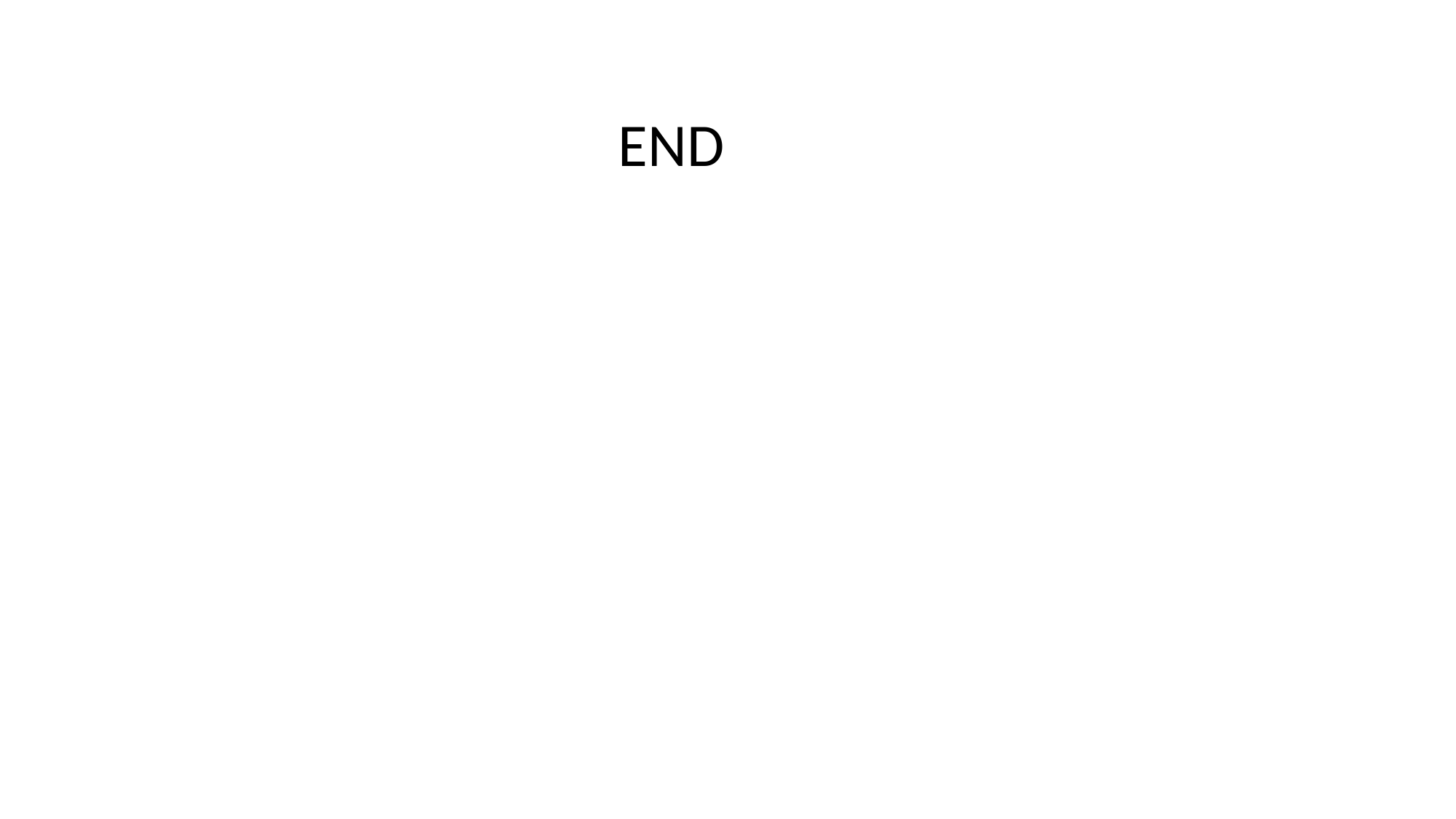

END

## Slide 25
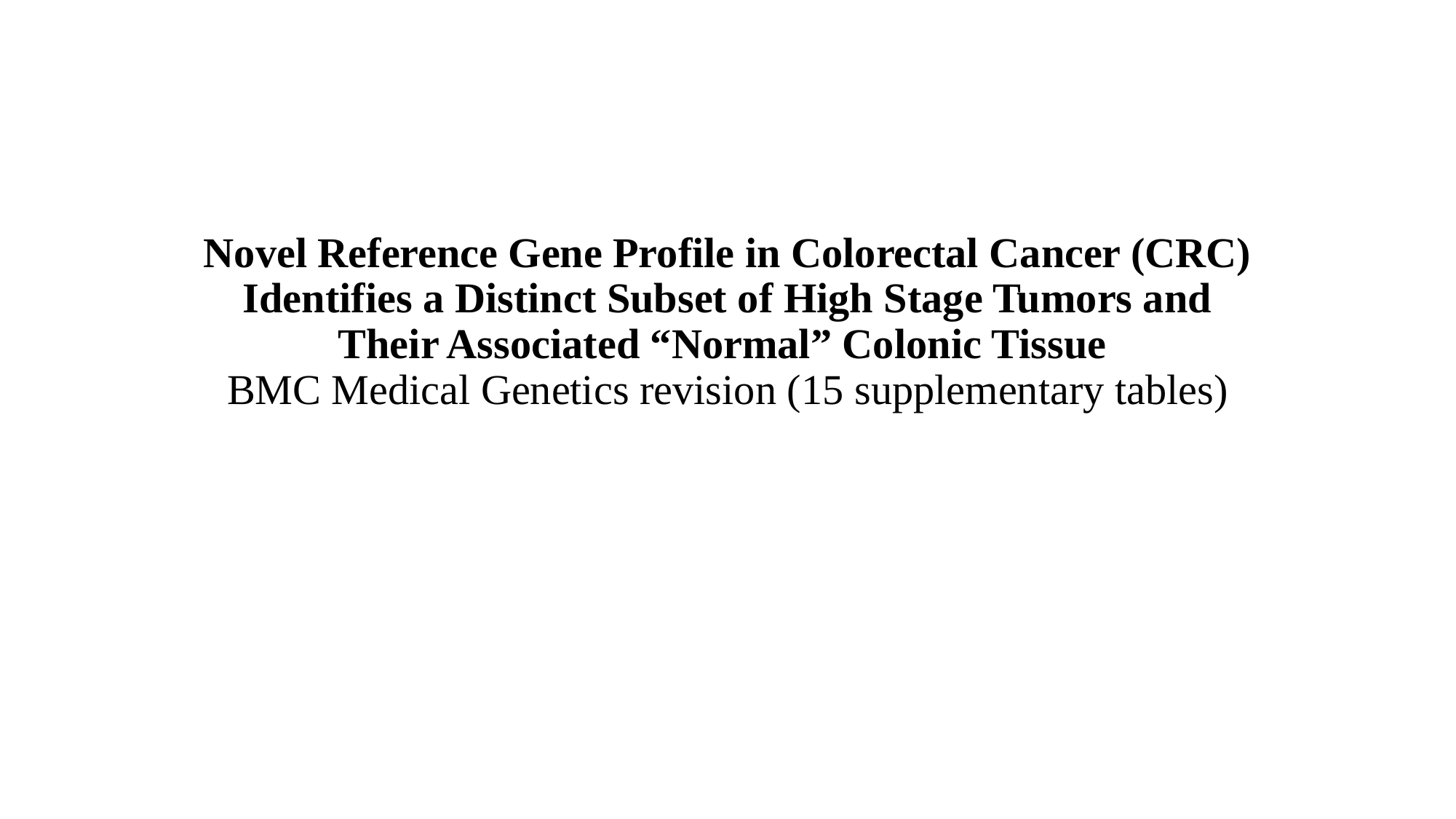

# Novel Reference Gene Profile in Colorectal Cancer (CRC) Identifies a Distinct Subset of High Stage Tumors and Their Associated “Normal” Colonic Tissue BMC Medical Genetics revision (15 supplementary tables)

## Slide 26
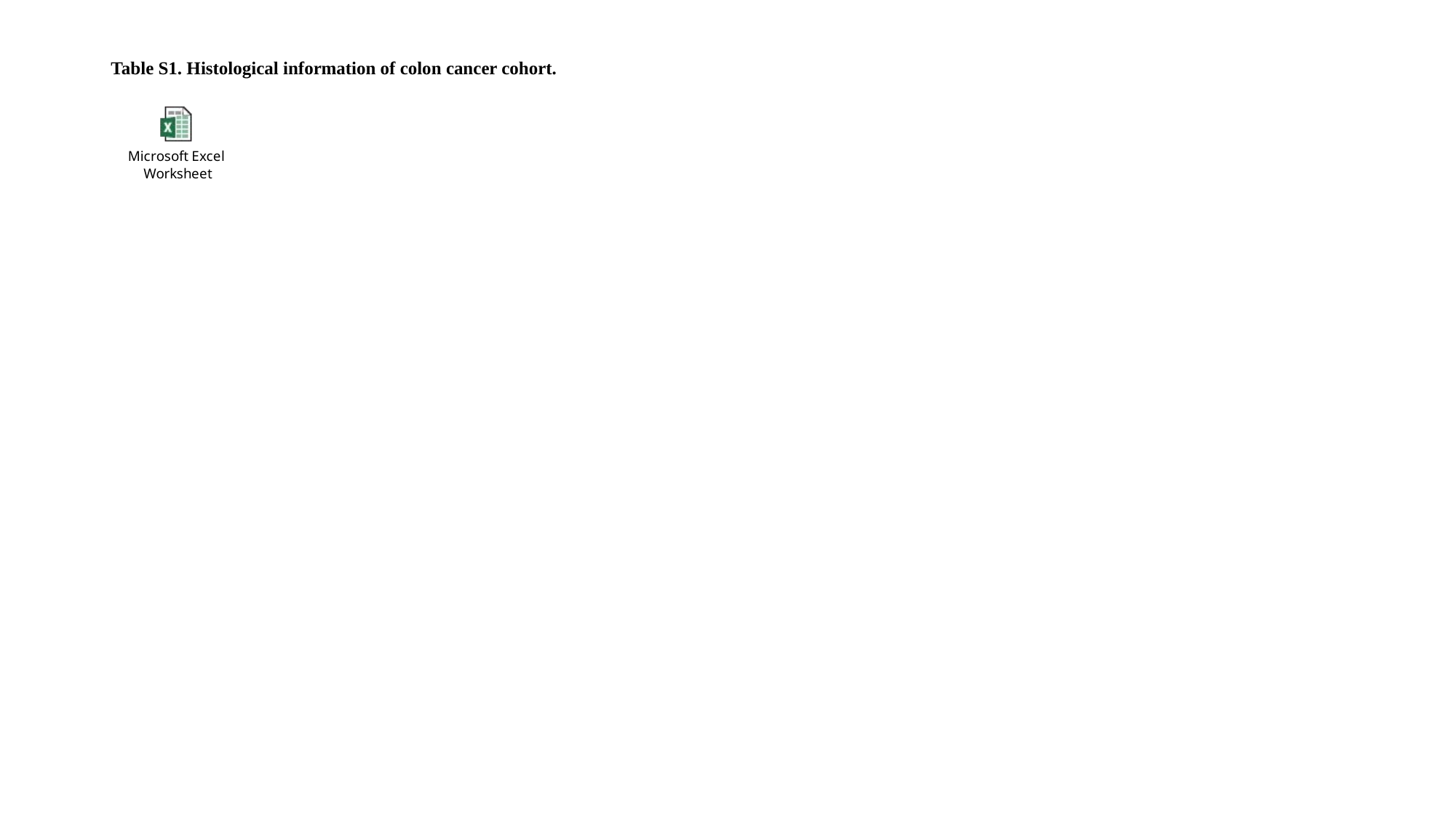

# Table S1. Histological information of colon cancer cohort.

## Slide 27
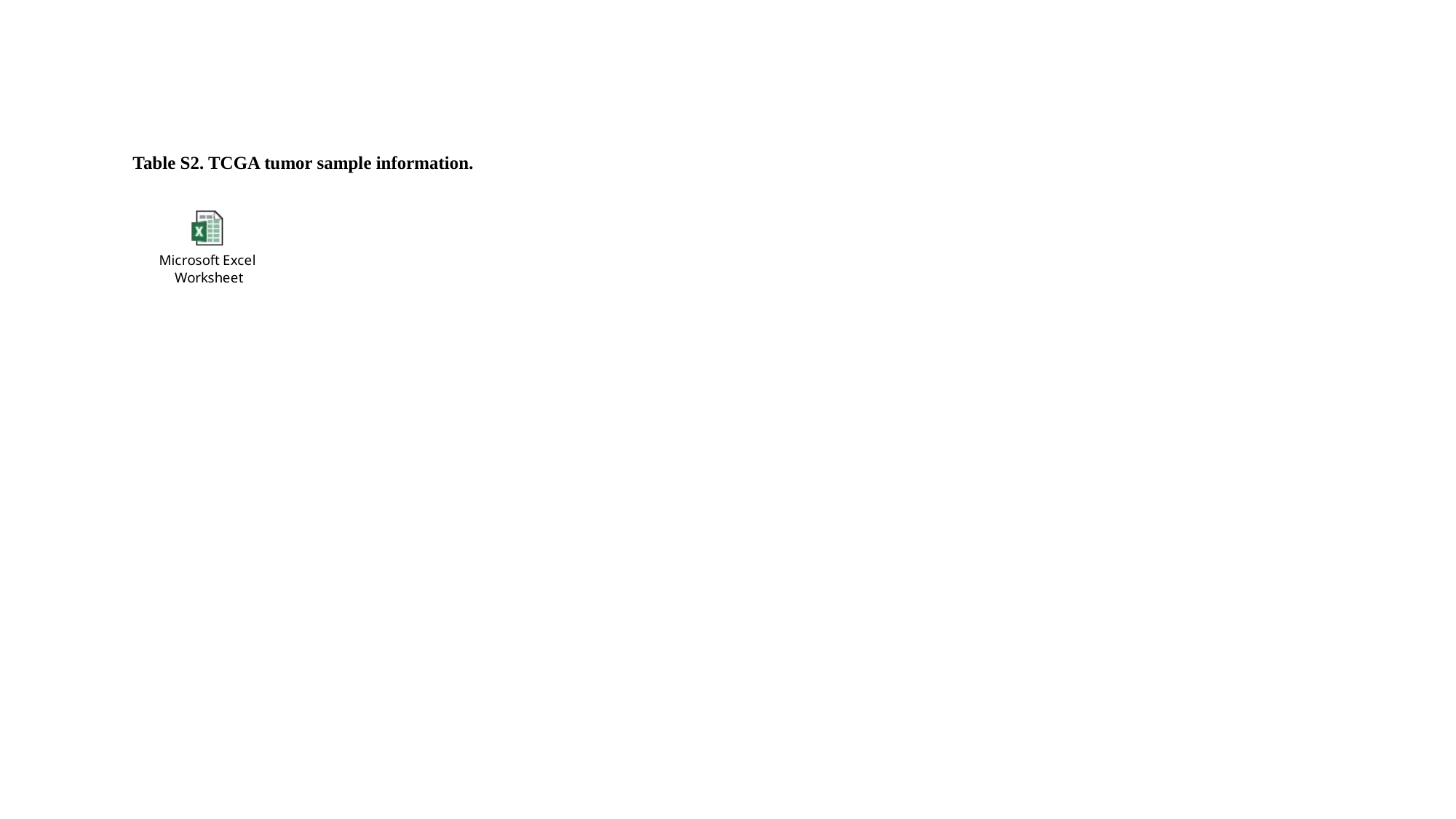

Table S2. TCGA tumor sample information.

## Slide 28
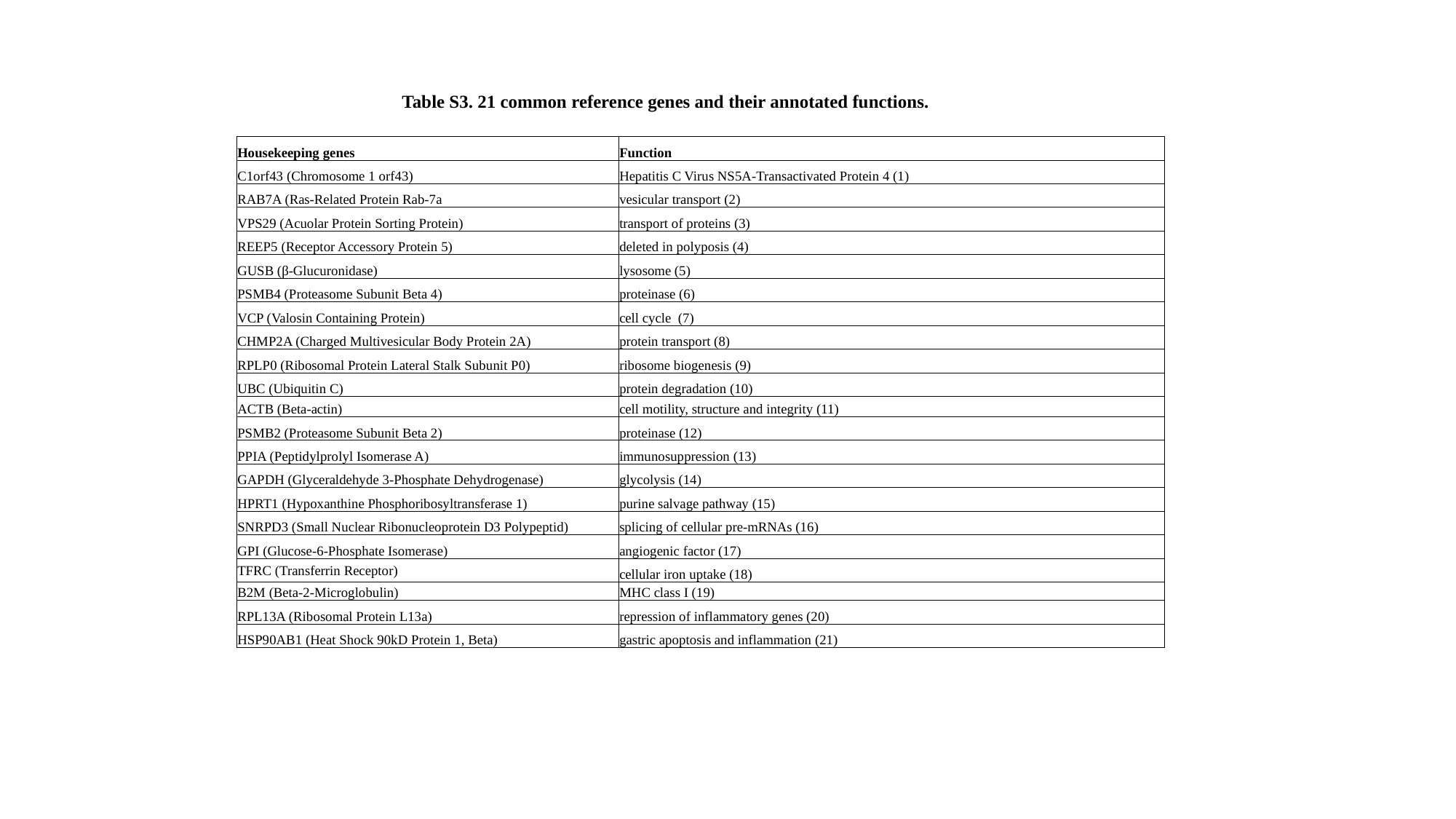

Table S3. 21 common reference genes and their annotated functions.
| Housekeeping genes | Function |
| --- | --- |
| C1orf43 (Chromosome 1 orf43) | Hepatitis C Virus NS5A-Transactivated Protein 4 (1) |
| RAB7A (Ras-Related Protein Rab-7a | vesicular transport (2) |
| VPS29 (Acuolar Protein Sorting Protein) | transport of proteins (3) |
| REEP5 (Receptor Accessory Protein 5) | deleted in polyposis (4) |
| GUSB (β-Glucuronidase) | lysosome (5) |
| PSMB4 (Proteasome Subunit Beta 4) | proteinase (6) |
| VCP (Valosin Containing Protein) | cell cycle (7) |
| CHMP2A (Charged Multivesicular Body Protein 2A) | protein transport (8) |
| RPLP0 (Ribosomal Protein Lateral Stalk Subunit P0) | ribosome biogenesis (9) |
| UBC (Ubiquitin C) | protein degradation (10) |
| ACTB (Beta-actin) | cell motility, structure and integrity (11) |
| PSMB2 (Proteasome Subunit Beta 2) | proteinase (12) |
| PPIA (Peptidylprolyl Isomerase A) | immunosuppression (13) |
| GAPDH (Glyceraldehyde 3-Phosphate Dehydrogenase) | glycolysis (14) |
| HPRT1 (Hypoxanthine Phosphoribosyltransferase 1) | purine salvage pathway (15) |
| SNRPD3 (Small Nuclear Ribonucleoprotein D3 Polypeptid) | splicing of cellular pre-mRNAs (16) |
| GPI (Glucose-6-Phosphate Isomerase) | angiogenic factor (17) |
| TFRC (Transferrin Receptor) | cellular iron uptake (18) |
| B2M (Beta-2-Microglobulin) | MHC class I (19) |
| RPL13A (Ribosomal Protein L13a) | repression of inflammatory genes (20) |
| HSP90AB1 (Heat Shock 90kD Protein 1, Beta) | gastric apoptosis and inflammation (21) |

## Slide 29
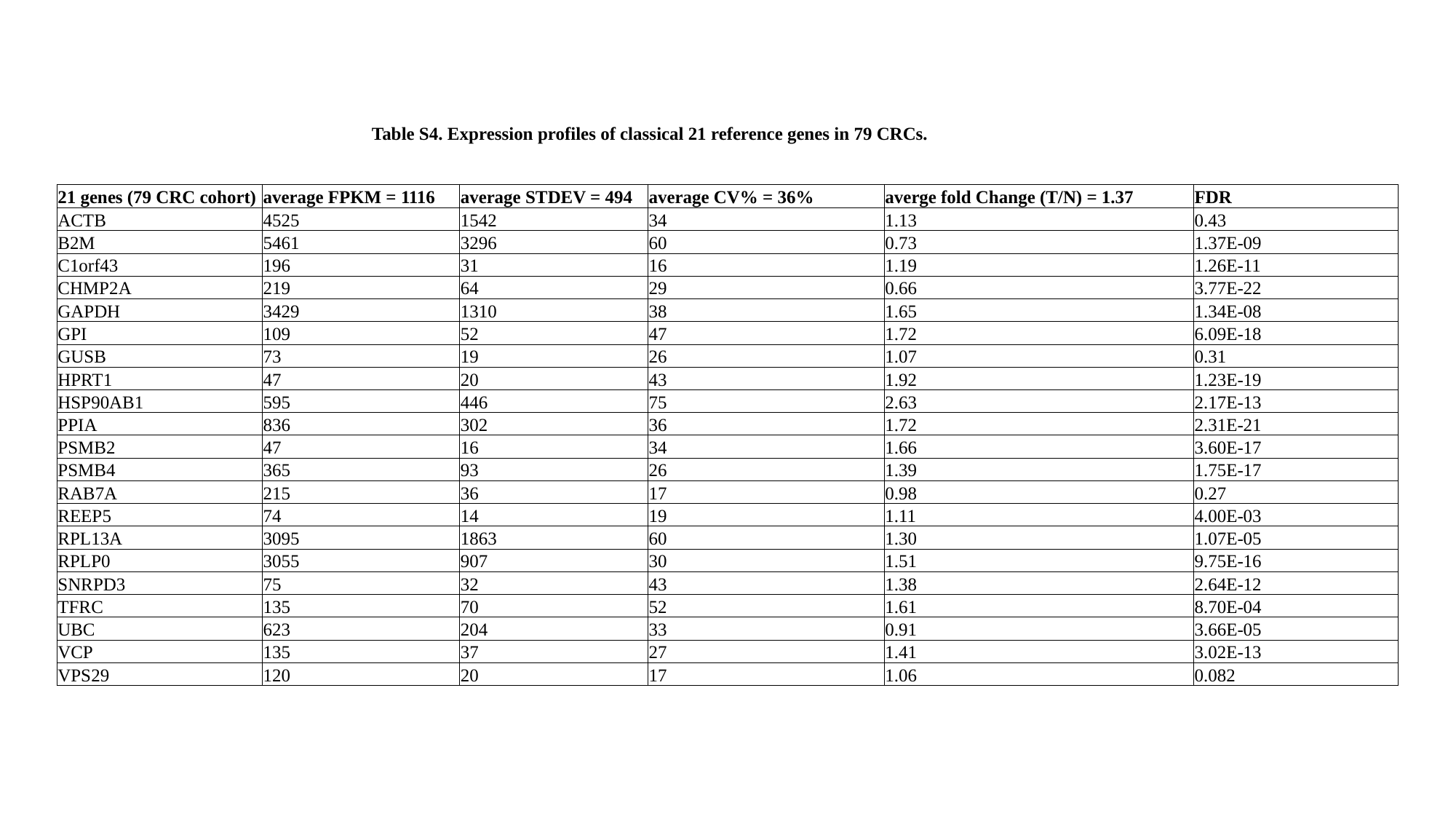

Table S4. Expression profiles of classical 21 reference genes in 79 CRCs.
| 21 genes (79 CRC cohort) | average FPKM = 1116 | average STDEV = 494 | average CV% = 36% | averge fold Change (T/N) = 1.37 | FDR |
| --- | --- | --- | --- | --- | --- |
| ACTB | 4525 | 1542 | 34 | 1.13 | 0.43 |
| B2M | 5461 | 3296 | 60 | 0.73 | 1.37E-09 |
| C1orf43 | 196 | 31 | 16 | 1.19 | 1.26E-11 |
| CHMP2A | 219 | 64 | 29 | 0.66 | 3.77E-22 |
| GAPDH | 3429 | 1310 | 38 | 1.65 | 1.34E-08 |
| GPI | 109 | 52 | 47 | 1.72 | 6.09E-18 |
| GUSB | 73 | 19 | 26 | 1.07 | 0.31 |
| HPRT1 | 47 | 20 | 43 | 1.92 | 1.23E-19 |
| HSP90AB1 | 595 | 446 | 75 | 2.63 | 2.17E-13 |
| PPIA | 836 | 302 | 36 | 1.72 | 2.31E-21 |
| PSMB2 | 47 | 16 | 34 | 1.66 | 3.60E-17 |
| PSMB4 | 365 | 93 | 26 | 1.39 | 1.75E-17 |
| RAB7A | 215 | 36 | 17 | 0.98 | 0.27 |
| REEP5 | 74 | 14 | 19 | 1.11 | 4.00E-03 |
| RPL13A | 3095 | 1863 | 60 | 1.30 | 1.07E-05 |
| RPLP0 | 3055 | 907 | 30 | 1.51 | 9.75E-16 |
| SNRPD3 | 75 | 32 | 43 | 1.38 | 2.64E-12 |
| TFRC | 135 | 70 | 52 | 1.61 | 8.70E-04 |
| UBC | 623 | 204 | 33 | 0.91 | 3.66E-05 |
| VCP | 135 | 37 | 27 | 1.41 | 3.02E-13 |
| VPS29 | 120 | 20 | 17 | 1.06 | 0.082 |

## Slide 30
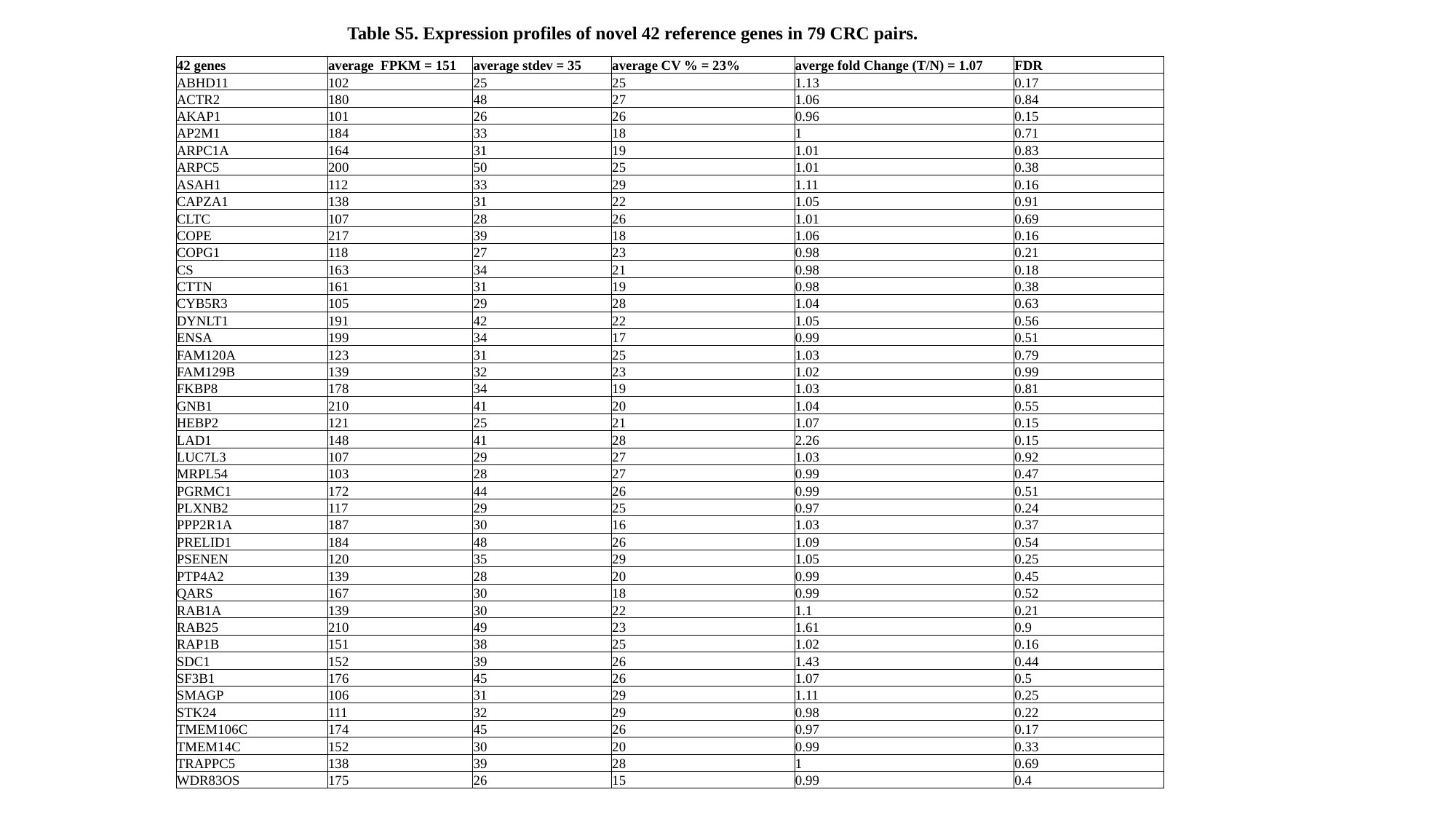

Table S5. Expression profiles of novel 42 reference genes in 79 CRC pairs.
| 42 genes | average FPKM = 151 | average stdev = 35 | average CV % = 23% | averge fold Change (T/N) = 1.07 | FDR |
| --- | --- | --- | --- | --- | --- |
| ABHD11 | 102 | 25 | 25 | 1.13 | 0.17 |
| ACTR2 | 180 | 48 | 27 | 1.06 | 0.84 |
| AKAP1 | 101 | 26 | 26 | 0.96 | 0.15 |
| AP2M1 | 184 | 33 | 18 | 1 | 0.71 |
| ARPC1A | 164 | 31 | 19 | 1.01 | 0.83 |
| ARPC5 | 200 | 50 | 25 | 1.01 | 0.38 |
| ASAH1 | 112 | 33 | 29 | 1.11 | 0.16 |
| CAPZA1 | 138 | 31 | 22 | 1.05 | 0.91 |
| CLTC | 107 | 28 | 26 | 1.01 | 0.69 |
| COPE | 217 | 39 | 18 | 1.06 | 0.16 |
| COPG1 | 118 | 27 | 23 | 0.98 | 0.21 |
| CS | 163 | 34 | 21 | 0.98 | 0.18 |
| CTTN | 161 | 31 | 19 | 0.98 | 0.38 |
| CYB5R3 | 105 | 29 | 28 | 1.04 | 0.63 |
| DYNLT1 | 191 | 42 | 22 | 1.05 | 0.56 |
| ENSA | 199 | 34 | 17 | 0.99 | 0.51 |
| FAM120A | 123 | 31 | 25 | 1.03 | 0.79 |
| FAM129B | 139 | 32 | 23 | 1.02 | 0.99 |
| FKBP8 | 178 | 34 | 19 | 1.03 | 0.81 |
| GNB1 | 210 | 41 | 20 | 1.04 | 0.55 |
| HEBP2 | 121 | 25 | 21 | 1.07 | 0.15 |
| LAD1 | 148 | 41 | 28 | 2.26 | 0.15 |
| LUC7L3 | 107 | 29 | 27 | 1.03 | 0.92 |
| MRPL54 | 103 | 28 | 27 | 0.99 | 0.47 |
| PGRMC1 | 172 | 44 | 26 | 0.99 | 0.51 |
| PLXNB2 | 117 | 29 | 25 | 0.97 | 0.24 |
| PPP2R1A | 187 | 30 | 16 | 1.03 | 0.37 |
| PRELID1 | 184 | 48 | 26 | 1.09 | 0.54 |
| PSENEN | 120 | 35 | 29 | 1.05 | 0.25 |
| PTP4A2 | 139 | 28 | 20 | 0.99 | 0.45 |
| QARS | 167 | 30 | 18 | 0.99 | 0.52 |
| RAB1A | 139 | 30 | 22 | 1.1 | 0.21 |
| RAB25 | 210 | 49 | 23 | 1.61 | 0.9 |
| RAP1B | 151 | 38 | 25 | 1.02 | 0.16 |
| SDC1 | 152 | 39 | 26 | 1.43 | 0.44 |
| SF3B1 | 176 | 45 | 26 | 1.07 | 0.5 |
| SMAGP | 106 | 31 | 29 | 1.11 | 0.25 |
| STK24 | 111 | 32 | 29 | 0.98 | 0.22 |
| TMEM106C | 174 | 45 | 26 | 0.97 | 0.17 |
| TMEM14C | 152 | 30 | 20 | 0.99 | 0.33 |
| TRAPPC5 | 138 | 39 | 28 | 1 | 0.69 |
| WDR83OS | 175 | 26 | 15 | 0.99 | 0.4 |

## Slide 31
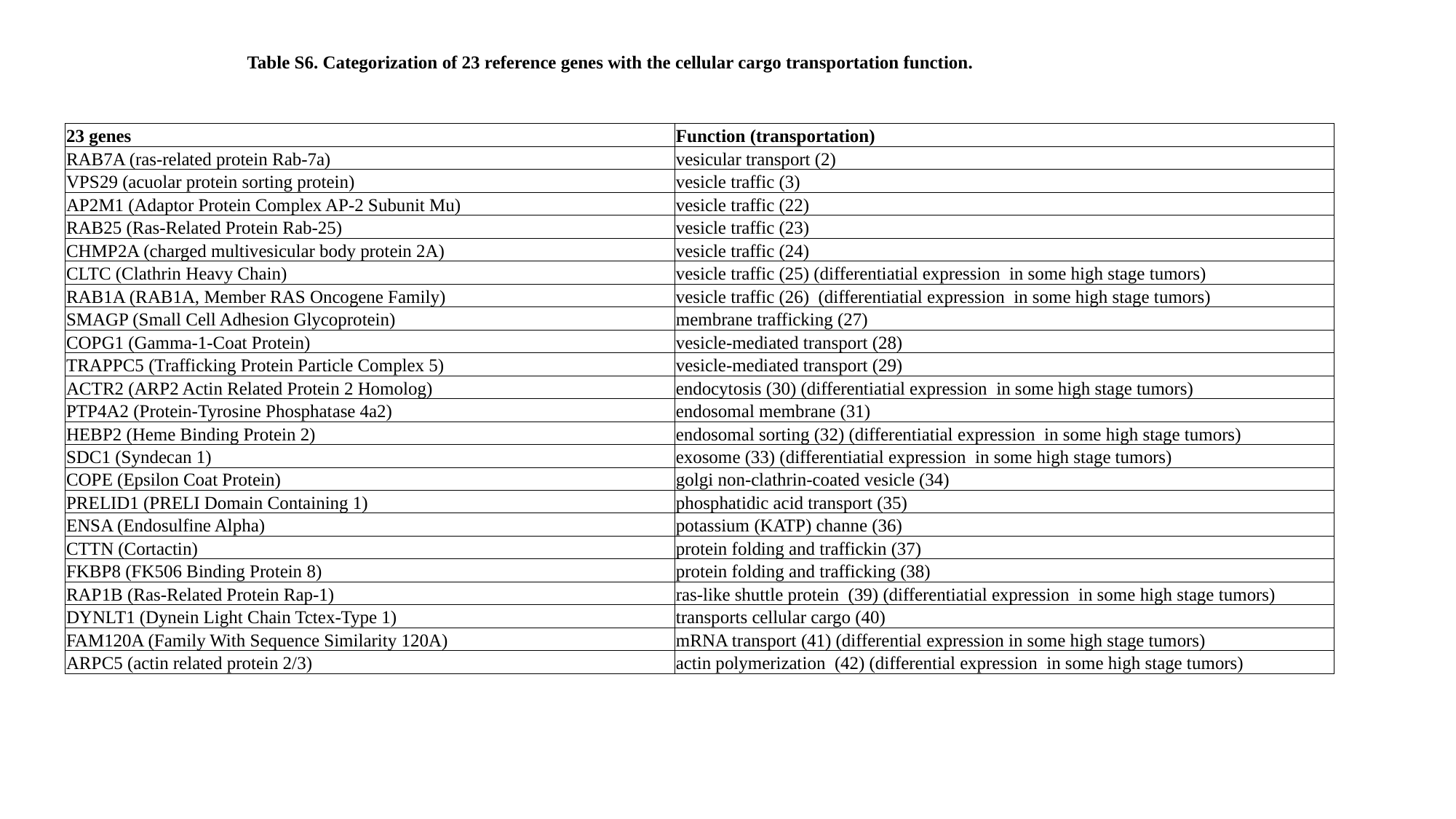

Table S6. Categorization of 23 reference genes with the cellular cargo transportation function.
| 23 genes | Function (transportation) |
| --- | --- |
| RAB7A (ras-related protein Rab-7a) | vesicular transport (2) |
| VPS29 (acuolar protein sorting protein) | vesicle traffic (3) |
| AP2M1 (Adaptor Protein Complex AP-2 Subunit Mu) | vesicle traffic (22) |
| RAB25 (Ras-Related Protein Rab-25) | vesicle traffic (23) |
| CHMP2A (charged multivesicular body protein 2A) | vesicle traffic (24) |
| CLTC (Clathrin Heavy Chain) | vesicle traffic (25) (differentiatial expression in some high stage tumors) |
| RAB1A (RAB1A, Member RAS Oncogene Family) | vesicle traffic (26) (differentiatial expression in some high stage tumors) |
| SMAGP (Small Cell Adhesion Glycoprotein) | membrane trafficking (27) |
| COPG1 (Gamma-1-Coat Protein) | vesicle-mediated transport (28) |
| TRAPPC5 (Trafficking Protein Particle Complex 5) | vesicle-mediated transport (29) |
| ACTR2 (ARP2 Actin Related Protein 2 Homolog) | endocytosis (30) (differentiatial expression in some high stage tumors) |
| PTP4A2 (Protein-Tyrosine Phosphatase 4a2) | endosomal membrane (31) |
| HEBP2 (Heme Binding Protein 2) | endosomal sorting (32) (differentiatial expression in some high stage tumors) |
| SDC1 (Syndecan 1) | exosome (33) (differentiatial expression in some high stage tumors) |
| COPE (Epsilon Coat Protein) | golgi non-clathrin-coated vesicle (34) |
| PRELID1 (PRELI Domain Containing 1) | phosphatidic acid transport (35) |
| ENSA (Endosulfine Alpha) | potassium (KATP) channe (36) |
| CTTN (Cortactin) | protein folding and traffickin (37) |
| FKBP8 (FK506 Binding Protein 8) | protein folding and trafficking (38) |
| RAP1B (Ras-Related Protein Rap-1) | ras-like shuttle protein (39) (differentiatial expression in some high stage tumors) |
| DYNLT1 (Dynein Light Chain Tctex-Type 1) | transports cellular cargo (40) |
| FAM120A (Family With Sequence Similarity 120A) | mRNA transport (41) (differential expression in some high stage tumors) |
| ARPC5 (actin related protein 2/3) | actin polymerization (42) (differential expression in some high stage tumors) |

## Slide 32
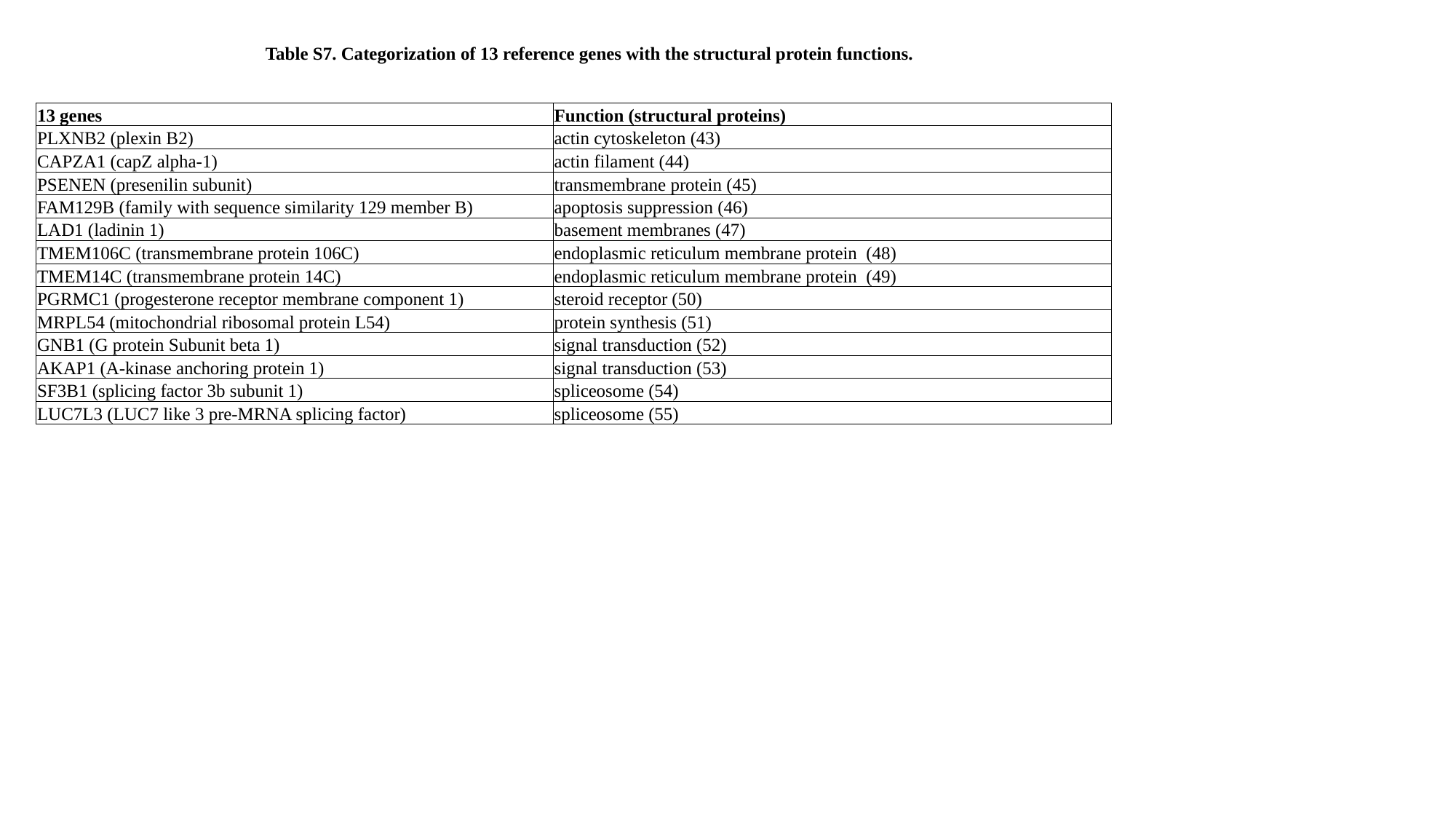

Table S7. Categorization of 13 reference genes with the structural protein functions.
| 13 genes | Function (structural proteins) |
| --- | --- |
| PLXNB2 (plexin B2) | actin cytoskeleton (43) |
| CAPZA1 (capZ alpha-1) | actin filament (44) |
| PSENEN (presenilin subunit) | transmembrane protein (45) |
| FAM129B (family with sequence similarity 129 member B) | apoptosis suppression (46) |
| LAD1 (ladinin 1) | basement membranes (47) |
| TMEM106C (transmembrane protein 106C) | endoplasmic reticulum membrane protein (48) |
| TMEM14C (transmembrane protein 14C) | endoplasmic reticulum membrane protein (49) |
| PGRMC1 (progesterone receptor membrane component 1) | steroid receptor (50) |
| MRPL54 (mitochondrial ribosomal protein L54) | protein synthesis (51) |
| GNB1 (G protein Subunit beta 1) | signal transduction (52) |
| AKAP1 (A-kinase anchoring protein 1) | signal transduction (53) |
| SF3B1 (splicing factor 3b subunit 1) | spliceosome (54) |
| LUC7L3 (LUC7 like 3 pre-MRNA splicing factor) | spliceosome (55) |

## Slide 33
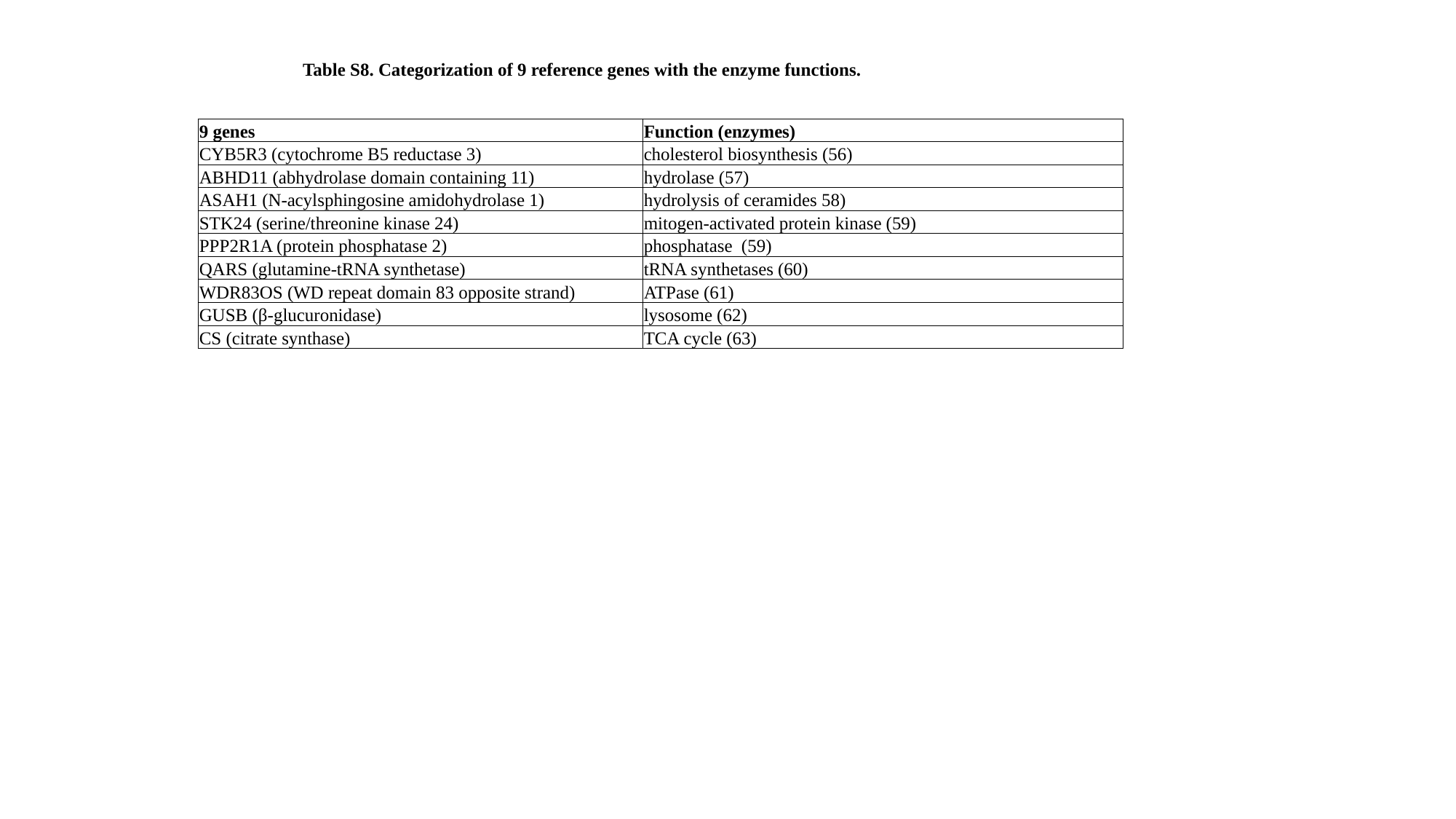

Table S8. Categorization of 9 reference genes with the enzyme functions.
| 9 genes | Function (enzymes) |
| --- | --- |
| CYB5R3 (cytochrome B5 reductase 3) | cholesterol biosynthesis (56) |
| ABHD11 (abhydrolase domain containing 11) | hydrolase (57) |
| ASAH1 (N-acylsphingosine amidohydrolase 1) | hydrolysis of ceramides 58) |
| STK24 (serine/threonine kinase 24) | mitogen-activated protein kinase (59) |
| PPP2R1A (protein phosphatase 2) | phosphatase (59) |
| QARS (glutamine-tRNA synthetase) | tRNA synthetases (60) |
| WDR83OS (WD repeat domain 83 opposite strand) | ATPase (61) |
| GUSB (β-glucuronidase) | lysosome (62) |
| CS (citrate synthase) | TCA cycle (63) |

## Slide 34
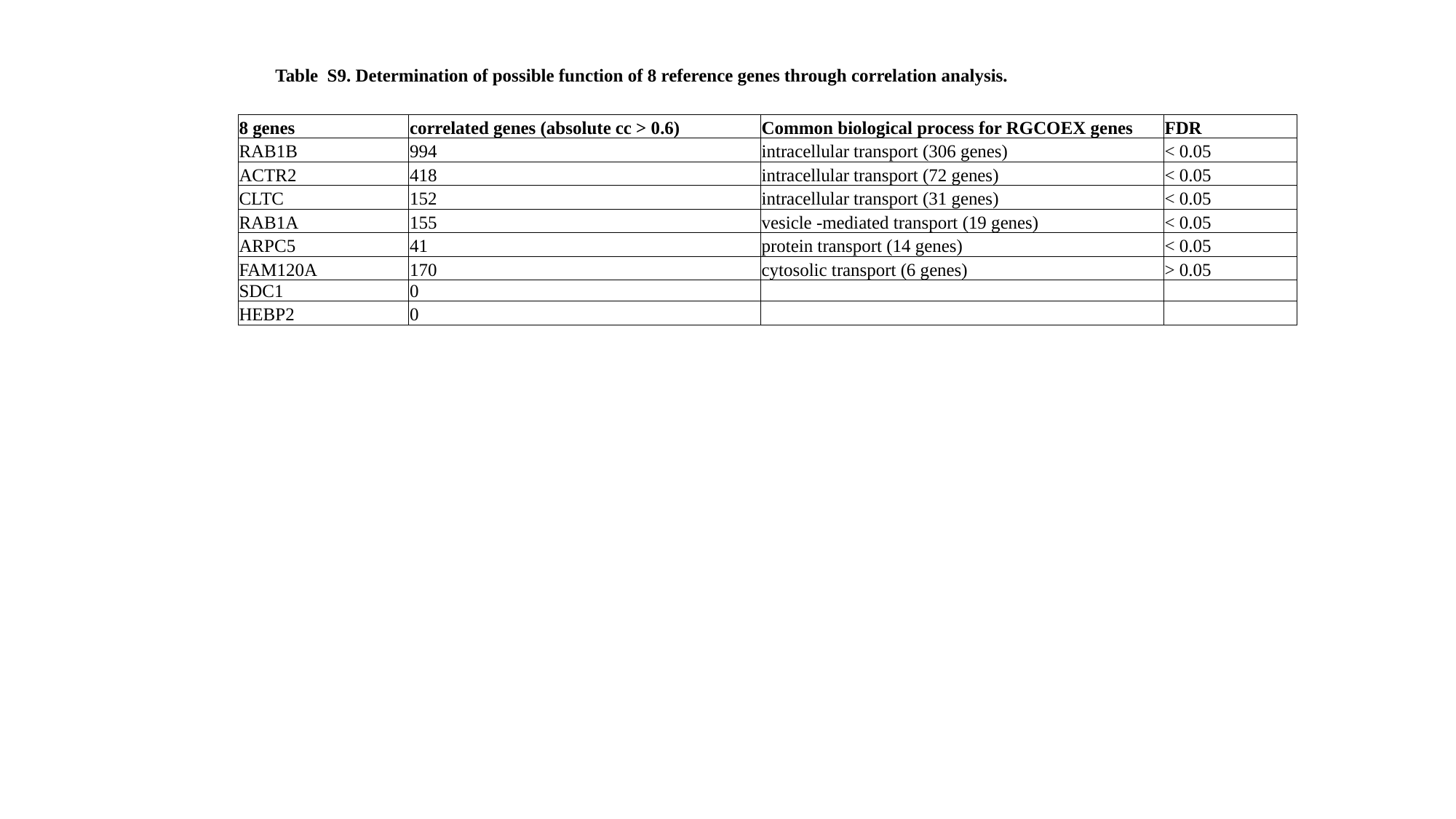

Table S9. Determination of possible function of 8 reference genes through correlation analysis.
| 8 genes | correlated genes (absolute cc > 0.6) | Common biological process for RGCOEX genes | FDR |
| --- | --- | --- | --- |
| RAB1B | 994 | intracellular transport (306 genes) | < 0.05 |
| ACTR2 | 418 | intracellular transport (72 genes) | < 0.05 |
| CLTC | 152 | intracellular transport (31 genes) | < 0.05 |
| RAB1A | 155 | vesicle -mediated transport (19 genes) | < 0.05 |
| ARPC5 | 41 | protein transport (14 genes) | < 0.05 |
| FAM120A | 170 | cytosolic transport (6 genes) | > 0.05 |
| SDC1 | 0 | | |
| HEBP2 | 0 | | |

## Slide 35
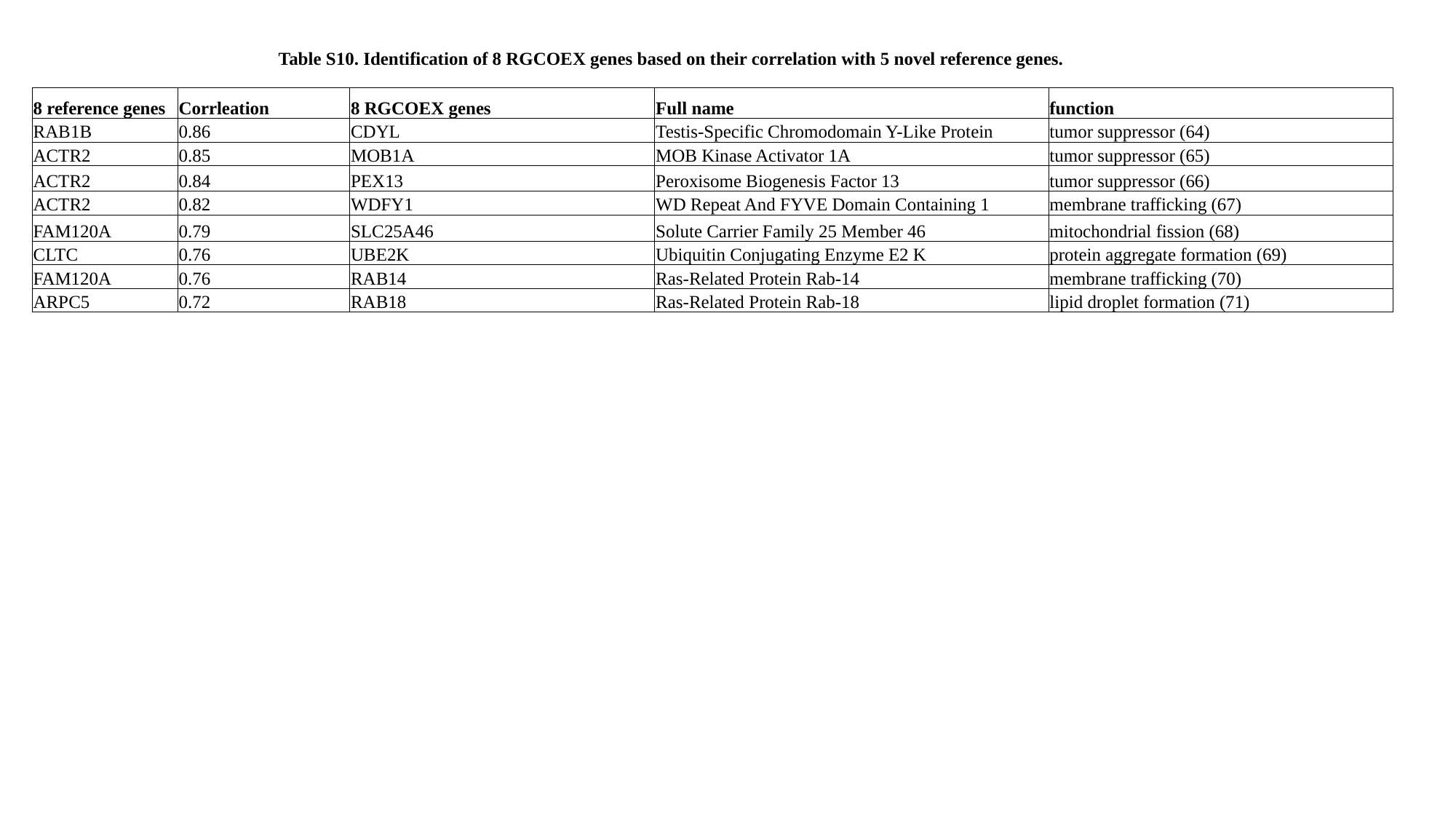

Table S10. Identification of 8 RGCOEX genes based on their correlation with 5 novel reference genes.
| 8 reference genes | Corrleation | 8 RGCOEX genes | Full name | function |
| --- | --- | --- | --- | --- |
| RAB1B | 0.86 | CDYL | Testis-Specific Chromodomain Y-Like Protein | tumor suppressor (64) |
| ACTR2 | 0.85 | MOB1A | MOB Kinase Activator 1A | tumor suppressor (65) |
| ACTR2 | 0.84 | PEX13 | Peroxisome Biogenesis Factor 13 | tumor suppressor (66) |
| ACTR2 | 0.82 | WDFY1 | WD Repeat And FYVE Domain Containing 1 | membrane trafficking (67) |
| FAM120A | 0.79 | SLC25A46 | Solute Carrier Family 25 Member 46 | mitochondrial fission (68) |
| CLTC | 0.76 | UBE2K | Ubiquitin Conjugating Enzyme E2 K | protein aggregate formation (69) |
| FAM120A | 0.76 | RAB14 | Ras-Related Protein Rab-14 | membrane trafficking (70) |
| ARPC5 | 0.72 | RAB18 | Ras-Related Protein Rab-18 | lipid droplet formation (71) |

## Slide 36
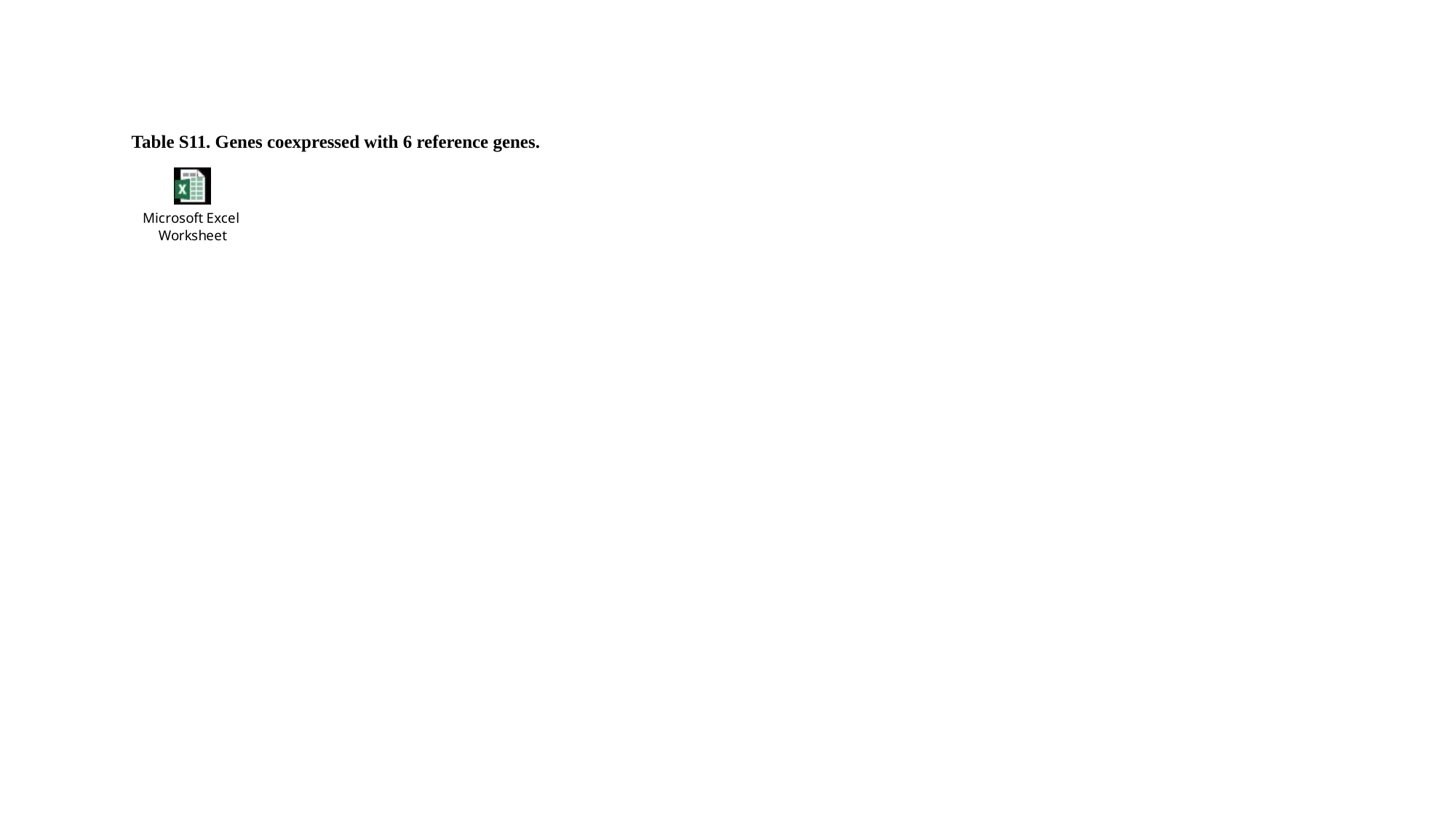

Table S11. Genes coexpressed with 6 reference genes.

## Slide 37
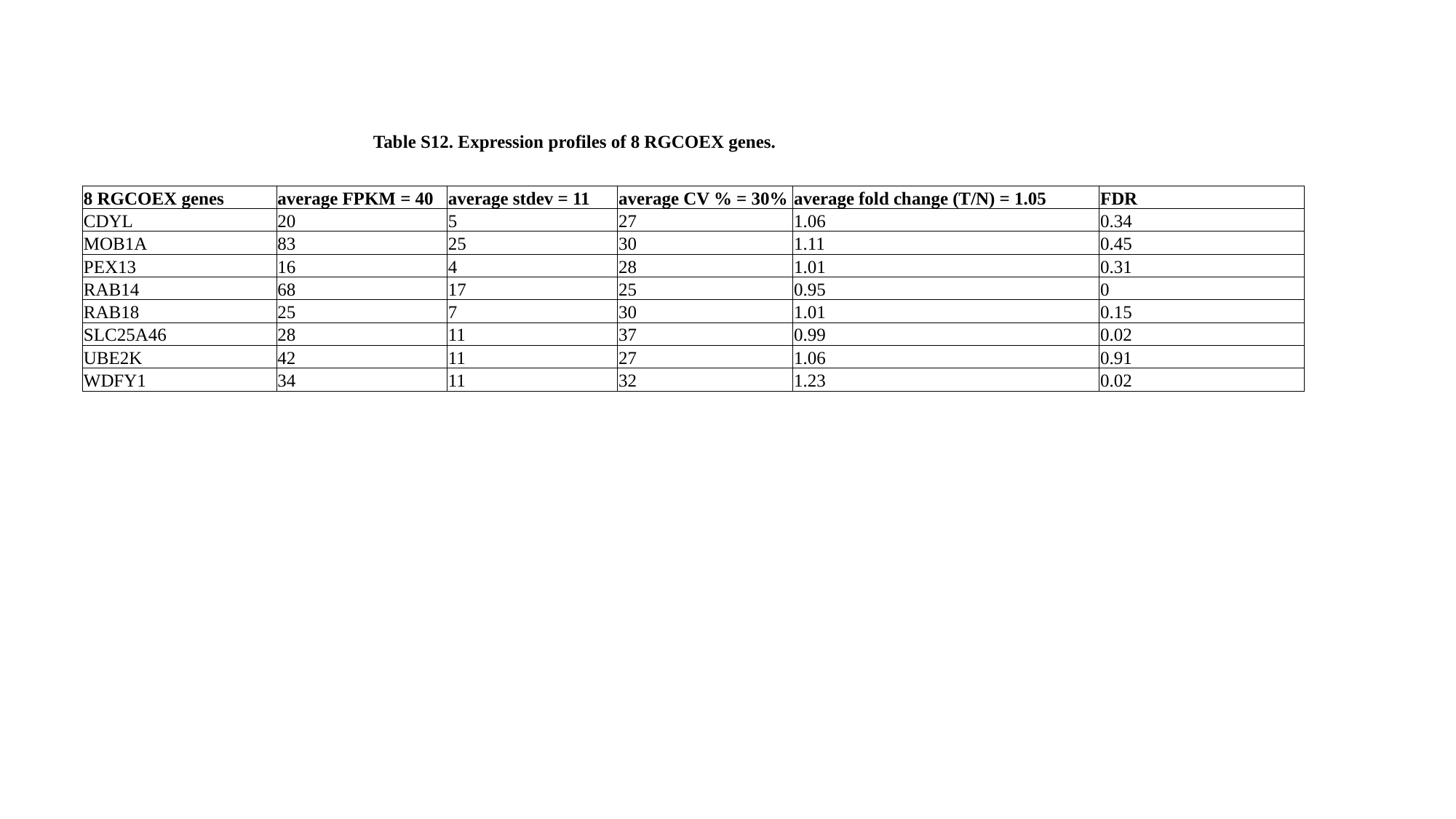

Table S12. Expression profiles of 8 RGCOEX genes.
| 8 RGCOEX genes | average FPKM = 40 | average stdev = 11 | average CV % = 30% | average fold change (T/N) = 1.05 | FDR |
| --- | --- | --- | --- | --- | --- |
| CDYL | 20 | 5 | 27 | 1.06 | 0.34 |
| MOB1A | 83 | 25 | 30 | 1.11 | 0.45 |
| PEX13 | 16 | 4 | 28 | 1.01 | 0.31 |
| RAB14 | 68 | 17 | 25 | 0.95 | 0 |
| RAB18 | 25 | 7 | 30 | 1.01 | 0.15 |
| SLC25A46 | 28 | 11 | 37 | 0.99 | 0.02 |
| UBE2K | 42 | 11 | 27 | 1.06 | 0.91 |
| WDFY1 | 34 | 11 | 32 | 1.23 | 0.02 |

## Slide 38
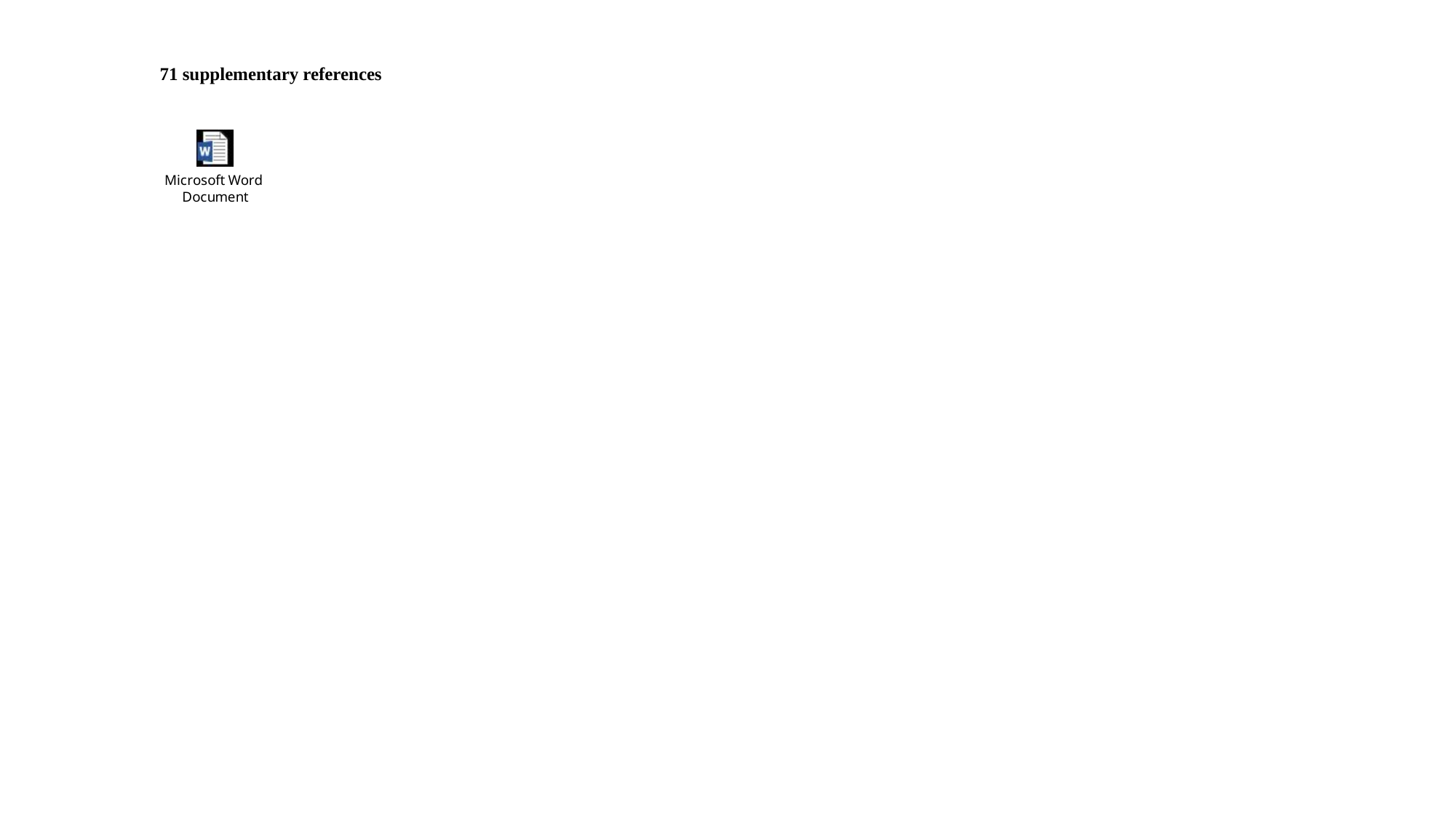

71 supplementary references

## Slide 39
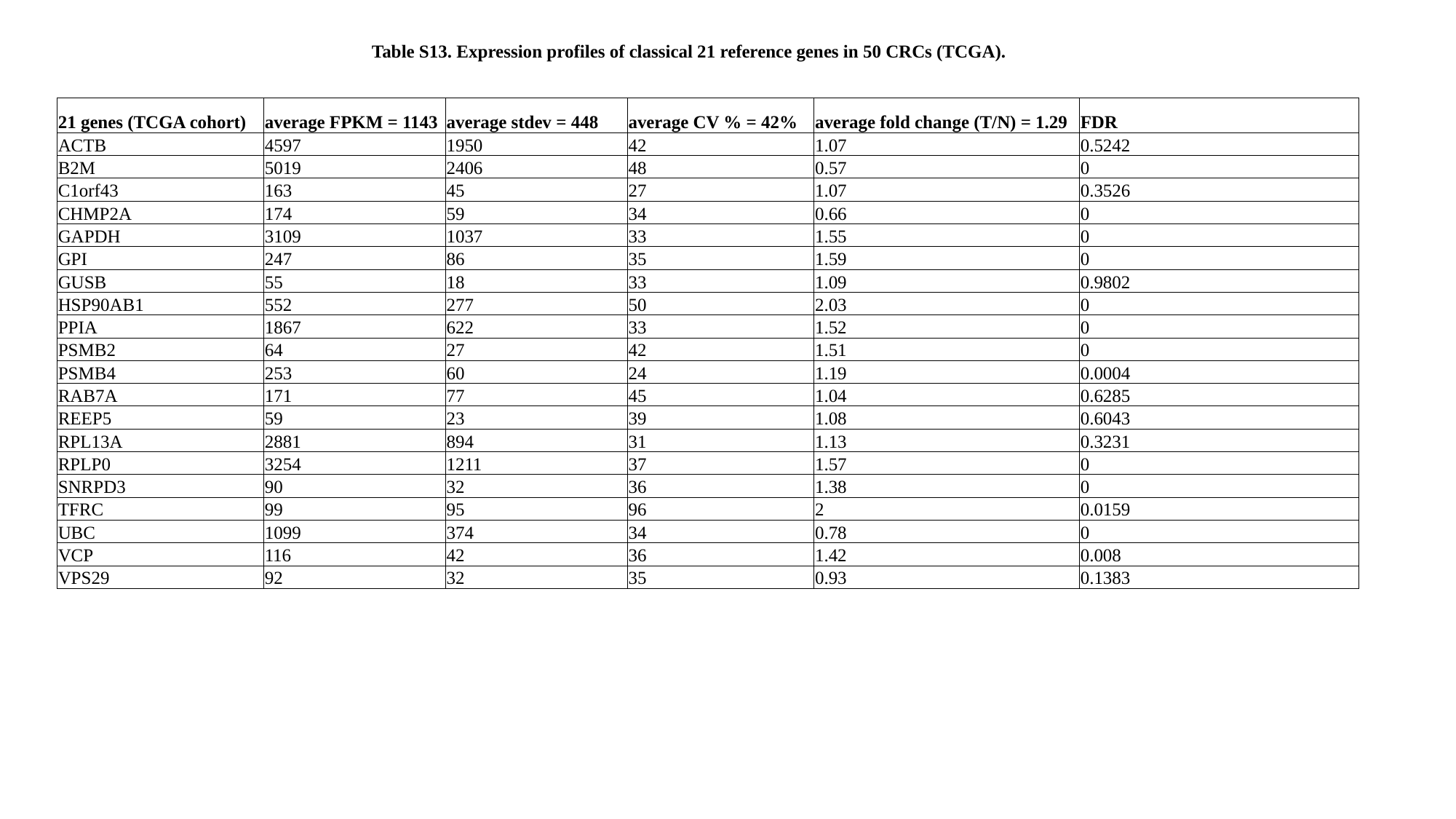

Table S13. Expression profiles of classical 21 reference genes in 50 CRCs (TCGA).
| 21 genes (TCGA cohort) | average FPKM = 1143 | average stdev = 448 | average CV % = 42% | average fold change (T/N) = 1.29 | FDR |
| --- | --- | --- | --- | --- | --- |
| ACTB | 4597 | 1950 | 42 | 1.07 | 0.5242 |
| B2M | 5019 | 2406 | 48 | 0.57 | 0 |
| C1orf43 | 163 | 45 | 27 | 1.07 | 0.3526 |
| CHMP2A | 174 | 59 | 34 | 0.66 | 0 |
| GAPDH | 3109 | 1037 | 33 | 1.55 | 0 |
| GPI | 247 | 86 | 35 | 1.59 | 0 |
| GUSB | 55 | 18 | 33 | 1.09 | 0.9802 |
| HSP90AB1 | 552 | 277 | 50 | 2.03 | 0 |
| PPIA | 1867 | 622 | 33 | 1.52 | 0 |
| PSMB2 | 64 | 27 | 42 | 1.51 | 0 |
| PSMB4 | 253 | 60 | 24 | 1.19 | 0.0004 |
| RAB7A | 171 | 77 | 45 | 1.04 | 0.6285 |
| REEP5 | 59 | 23 | 39 | 1.08 | 0.6043 |
| RPL13A | 2881 | 894 | 31 | 1.13 | 0.3231 |
| RPLP0 | 3254 | 1211 | 37 | 1.57 | 0 |
| SNRPD3 | 90 | 32 | 36 | 1.38 | 0 |
| TFRC | 99 | 95 | 96 | 2 | 0.0159 |
| UBC | 1099 | 374 | 34 | 0.78 | 0 |
| VCP | 116 | 42 | 36 | 1.42 | 0.008 |
| VPS29 | 92 | 32 | 35 | 0.93 | 0.1383 |

## Slide 40
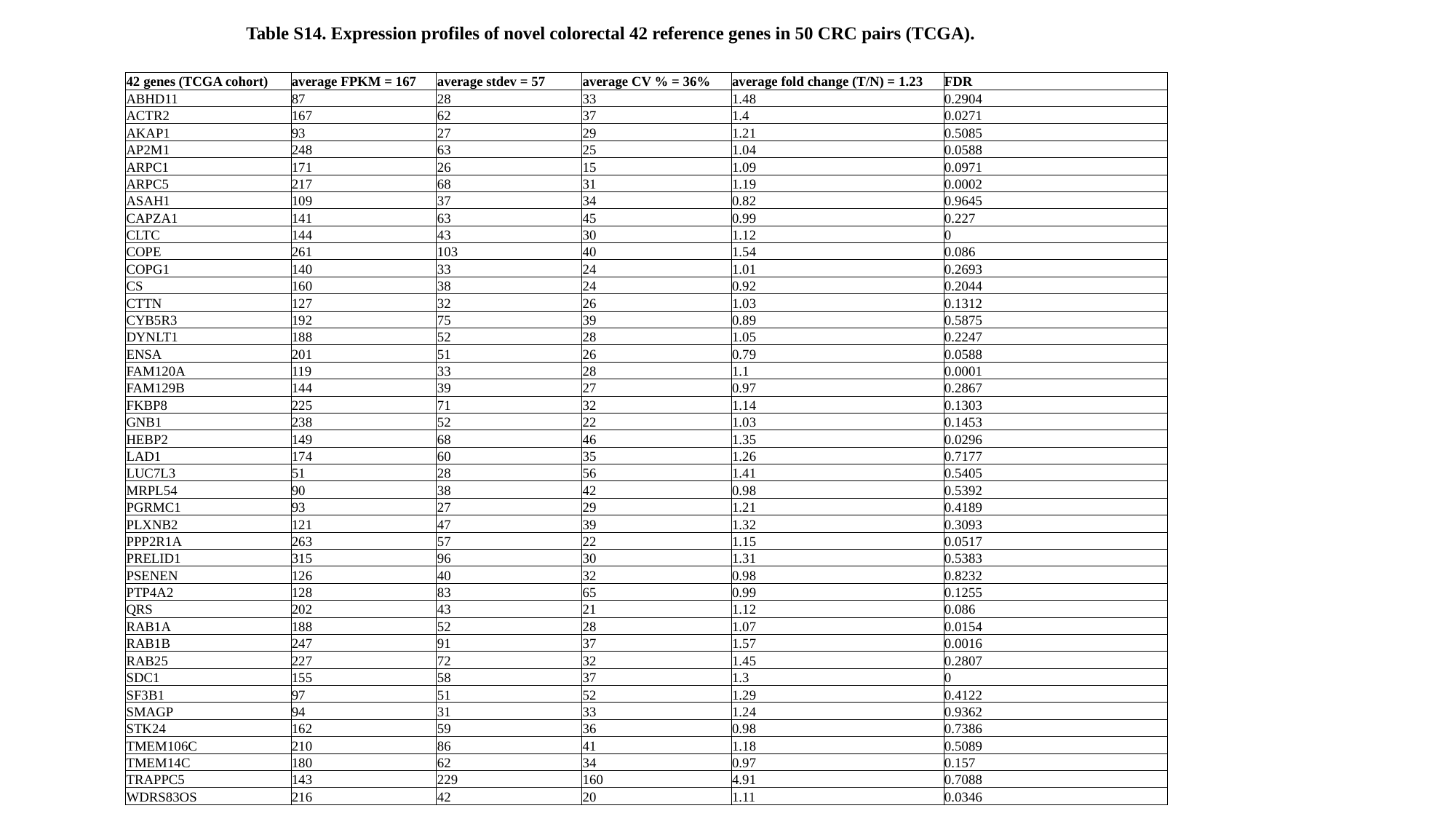

Table S14. Expression profiles of novel colorectal 42 reference genes in 50 CRC pairs (TCGA).
| 42 genes (TCGA cohort) | average FPKM = 167 | average stdev = 57 | average CV % = 36% | average fold change (T/N) = 1.23 | FDR |
| --- | --- | --- | --- | --- | --- |
| ABHD11 | 87 | 28 | 33 | 1.48 | 0.2904 |
| ACTR2 | 167 | 62 | 37 | 1.4 | 0.0271 |
| AKAP1 | 93 | 27 | 29 | 1.21 | 0.5085 |
| AP2M1 | 248 | 63 | 25 | 1.04 | 0.0588 |
| ARPC1 | 171 | 26 | 15 | 1.09 | 0.0971 |
| ARPC5 | 217 | 68 | 31 | 1.19 | 0.0002 |
| ASAH1 | 109 | 37 | 34 | 0.82 | 0.9645 |
| CAPZA1 | 141 | 63 | 45 | 0.99 | 0.227 |
| CLTC | 144 | 43 | 30 | 1.12 | 0 |
| COPE | 261 | 103 | 40 | 1.54 | 0.086 |
| COPG1 | 140 | 33 | 24 | 1.01 | 0.2693 |
| CS | 160 | 38 | 24 | 0.92 | 0.2044 |
| CTTN | 127 | 32 | 26 | 1.03 | 0.1312 |
| CYB5R3 | 192 | 75 | 39 | 0.89 | 0.5875 |
| DYNLT1 | 188 | 52 | 28 | 1.05 | 0.2247 |
| ENSA | 201 | 51 | 26 | 0.79 | 0.0588 |
| FAM120A | 119 | 33 | 28 | 1.1 | 0.0001 |
| FAM129B | 144 | 39 | 27 | 0.97 | 0.2867 |
| FKBP8 | 225 | 71 | 32 | 1.14 | 0.1303 |
| GNB1 | 238 | 52 | 22 | 1.03 | 0.1453 |
| HEBP2 | 149 | 68 | 46 | 1.35 | 0.0296 |
| LAD1 | 174 | 60 | 35 | 1.26 | 0.7177 |
| LUC7L3 | 51 | 28 | 56 | 1.41 | 0.5405 |
| MRPL54 | 90 | 38 | 42 | 0.98 | 0.5392 |
| PGRMC1 | 93 | 27 | 29 | 1.21 | 0.4189 |
| PLXNB2 | 121 | 47 | 39 | 1.32 | 0.3093 |
| PPP2R1A | 263 | 57 | 22 | 1.15 | 0.0517 |
| PRELID1 | 315 | 96 | 30 | 1.31 | 0.5383 |
| PSENEN | 126 | 40 | 32 | 0.98 | 0.8232 |
| PTP4A2 | 128 | 83 | 65 | 0.99 | 0.1255 |
| QRS | 202 | 43 | 21 | 1.12 | 0.086 |
| RAB1A | 188 | 52 | 28 | 1.07 | 0.0154 |
| RAB1B | 247 | 91 | 37 | 1.57 | 0.0016 |
| RAB25 | 227 | 72 | 32 | 1.45 | 0.2807 |
| SDC1 | 155 | 58 | 37 | 1.3 | 0 |
| SF3B1 | 97 | 51 | 52 | 1.29 | 0.4122 |
| SMAGP | 94 | 31 | 33 | 1.24 | 0.9362 |
| STK24 | 162 | 59 | 36 | 0.98 | 0.7386 |
| TMEM106C | 210 | 86 | 41 | 1.18 | 0.5089 |
| TMEM14C | 180 | 62 | 34 | 0.97 | 0.157 |
| TRAPPC5 | 143 | 229 | 160 | 4.91 | 0.7088 |
| WDRS83OS | 216 | 42 | 20 | 1.11 | 0.0346 |

## Slide 41
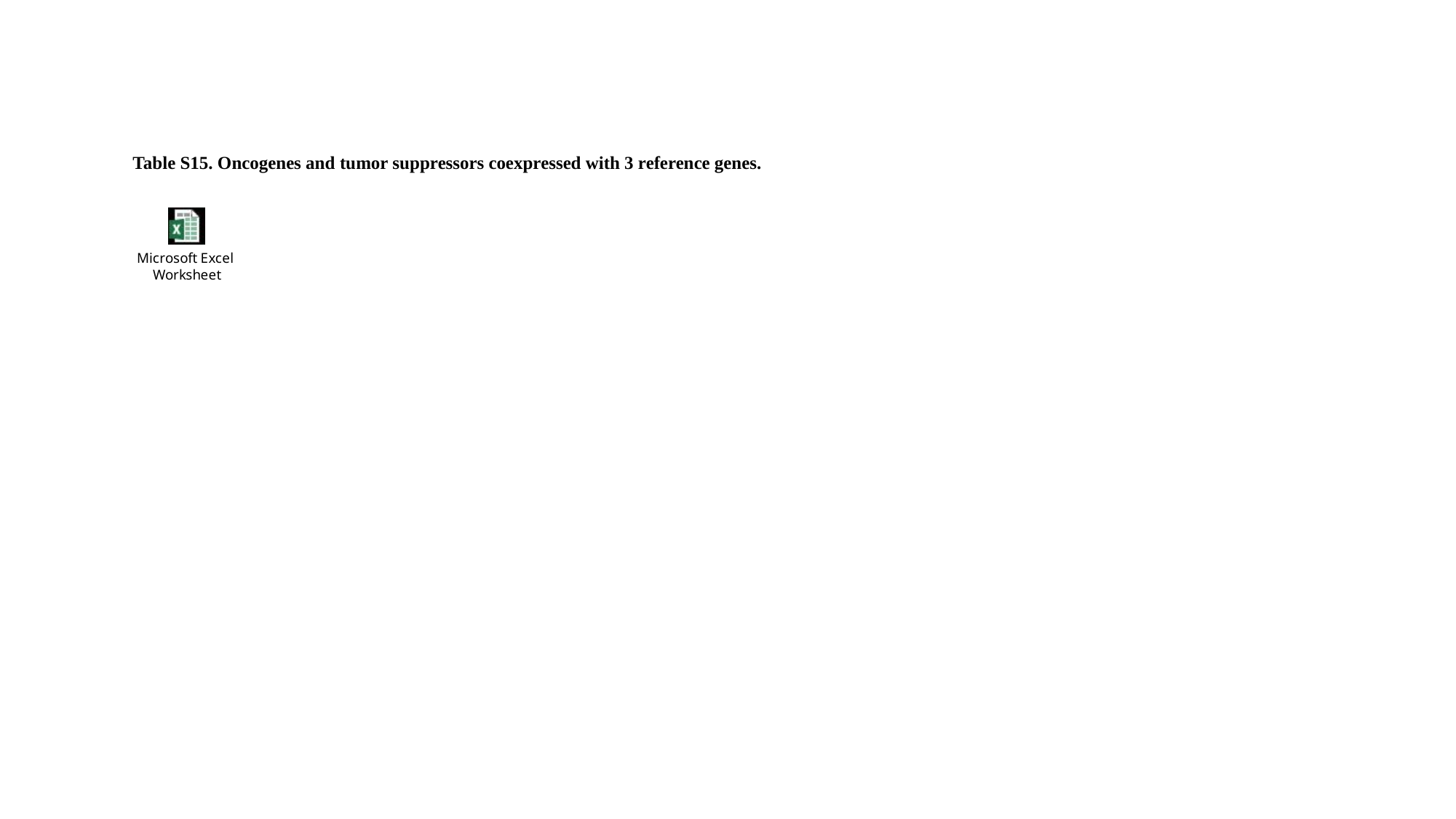

Table S15. Oncogenes and tumor suppressors coexpressed with 3 reference genes.

## Slide 42
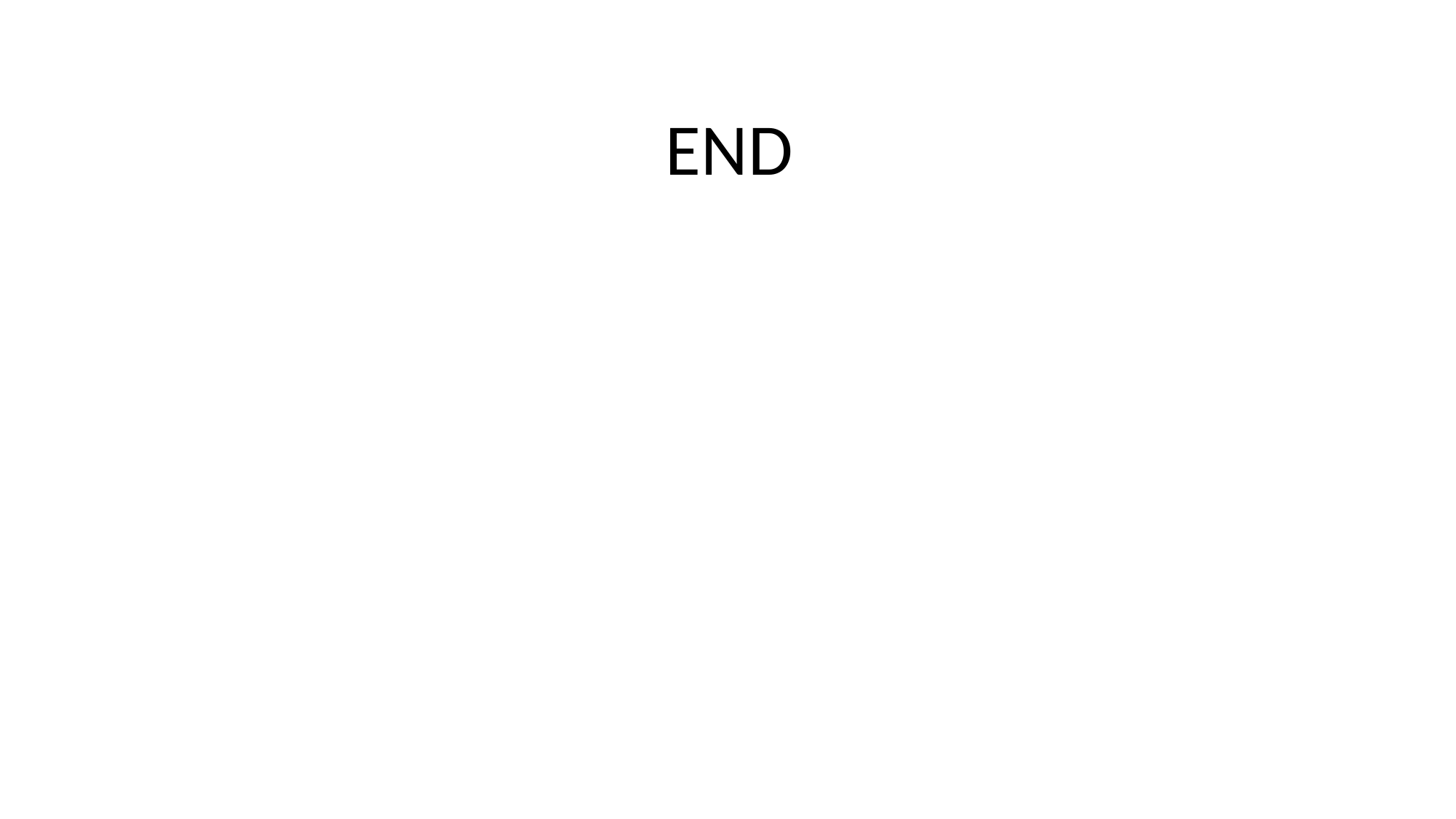

END
